# Shared pathogenic genes and therapeutic targets in periodontitis and systemic juvenile idiopathic arthritis: An integrative bioinformatics and machine learning study

**DOI:** 10.1371/journal.pcbi.1014669

**Published:** 2026-09-23

**Authors:** Qingao Deng, Junjie Wang, Xing Wang, Lu Qi

**Affiliations:** 1 The First Affiliated Hospital of Xinjiang Medical University, Urumqi, Xinjiang Uygur Autonomous Region, China; 2 The Second Affiliated Hospital of Xinjiang Medical University, Urumqi, Xinjiang Uygur Autonomous Region, China; University of Würzburg: Julius-Maximilians-Universitat Wurzburg, GERMANY

## Abstract

Periodontitis (PD) and systemic juvenile idiopathic arthritis (sJIA) are chronic inflammatory diseases with potential clinical links, yet their shared molecular mechanisms remain unclear—patients with JIA face elevated periodontal risk, the temporomandibular joint affected in JIA shares inflammatory pathways with PD, Th17 cells drive both conditions, and glucocorticoid therapy may exacerbate periodontal vulnerability. This study aimed to explore the shared mechanisms and potential therapeutic targets of PD and sJIA using genetic expression data from the GEO database and performed comprehensive bioinformatics analyses, including differential expression gene analysis, weighted gene co‑expression network analysis (WGCNA), functional enrichment analysis, protein‑protein interaction network construction, and machine learning across 175 predictive models with the Ridge + AdaBoost ensemble identified as the best‑performing combination, followed by SHapley Additive exPlanations (SHAP) for model interpretability, CIBERSORT for immune infiltration assessment, and molecular docking with 100‑ns molecular dynamics simulations for therapeutic target validation. We identified 37 shared candidate genes between PD and sJIA, which were significantly enriched in IL‑17, NF‑κB, rheumatoid arthritis, and lipid atherosclerosis pathways, and machine learning screening further selected six core diagnostic genes (*FAM46C*, *CXCL1*, *SELP*, *LGALSL*, *ELOVL4*, *VCAN*), with *SELP* demonstrating the most robust cross‑model, cross‑dataset diagnostic value (AUC > 0.8); SHAP analysis confirmed *SELP* as a stable risk‑driving predictor across all models, while *CXCL1* consistently showed protective effects. Immune infiltration revealed shared neutrophil elevation and CD8 ⁺ T‑cell reduction, with conserved *CXCL1* and *SELP* correlations with neutrophils and resting mast cells, and *FAM46C* with plasma cells.Drug‑target network analysis identified *IL1B, MMP*1, and *ITGAM* as core targets, and molecular docking yielded strong binding affinities for deoxycholic acid–*MMP1* (−7.6 kcal/mol), kaempferol–*IL1B* (−7.2 kcal/mol), kaempferol–*ITGAM* (−6.9 kcal/mol), with MD simulations confirming stable and specific binding. Collectively, this study explores shared genetic and immunological characteristics between PD and sJIA, suggests that *SELP* may serve as a critical cross‑disease diagnostic biomarker, and offers novel insights into their pathogenesis and potential therapeutic targets that warrant further experimental validation.

## Author summary

Periodontitis, a common oral disease, and systemic juvenile idiopathic arthritis, a rare autoimmune condition in children, have been clinically linked, yet their shared molecular basis remains unclear. By analyzing gene expression data from patients with each disease using network-based and machine learning approaches, we identified 37 genes commonly altered in both conditions. Among these, we pinpointed *SELP* as the strongest candidate for a shared diagnostic marker. We also found that both diseases involve elevated levels of neutrophils and reduction in CD8 ⁺ T cell, and identified several natural compounds that may bind to key proteins driving these diseases. Our findings provide a foundation for understanding how these two seemingly distinct conditions may be connected at the molecular level and highlight *SELP* and other genes as potential targets for future diagnostics or therapies. However, these are computational predictions that require experimental and clinical validation before they can inform patient care.

## 1. Introduction

Periodontitis(PD) is a prevalent condition that manifests in the global population. Its prevalence is estimated to be 11% [1]. The clinical manifestations of PD primarily manifest as a deterioration of the gingiva, periodontal tissue, alveolar bone, and dentin due to bacterial infection [2]. The aetiology of the condition is the result of an interaction between oral bacteria and the host’s immune system, leading to inflammation and immune responses mediated by the host’s own immune response [3]. The extant evidence suggests a link between PD and a variety of systemic diseases, including autoimmune disorders [4].

Systemic Juvenile Idiopathic Arthritis(sJIA) is a rare form of juvenile idiopathic arthritis(JIA) characterised by fever, skin eruptions, and systemic inflammation, whose prevalence is estimated to range between 10% and 20% of all cases of JIA [5].

Periodontitis, a prevalent chronic infectious inflammatory disease of the oral cavity, has garnered considerable attention in interdisciplinary research due to its association with autoimmune diseases. Compared to other types of JIA, sJIA’s systemic inflammation storm makes it more unique in how it interacts with other types of JIA. Studies have shown that JIA patients are more likely to develop severe gum disease and periodontal disease [6]. In JIA cases, the temporomandibular joint (TMJ) is often affected, and there is a link between JIA and periodontal disease, based on the similarity of the two conditions’ clinical inflammatory processes [7]. Th17 cells play a crucial role in many self-immune diseases, and their release contributes to PD, while cells associated with the Th1/Th17/Th22 axis are involved in temporomandibular joint osteoarthritis [8,9]. Additionally, the treatment for sJIA may affect oral health by regulating the immune system or altering the oral microenvironment. For example, glucocorticoids (such as prednisone) are commonly used to treat JIA, but long-term use can lead to gum overgrowth and disrupt the balance of oral bacteria, increasing the risk of periodontal disease. Research has shown that patients who use glucocorticoids for a long time are 20% to 30% more likely to develop gum disease [10].

**Fig 1:**
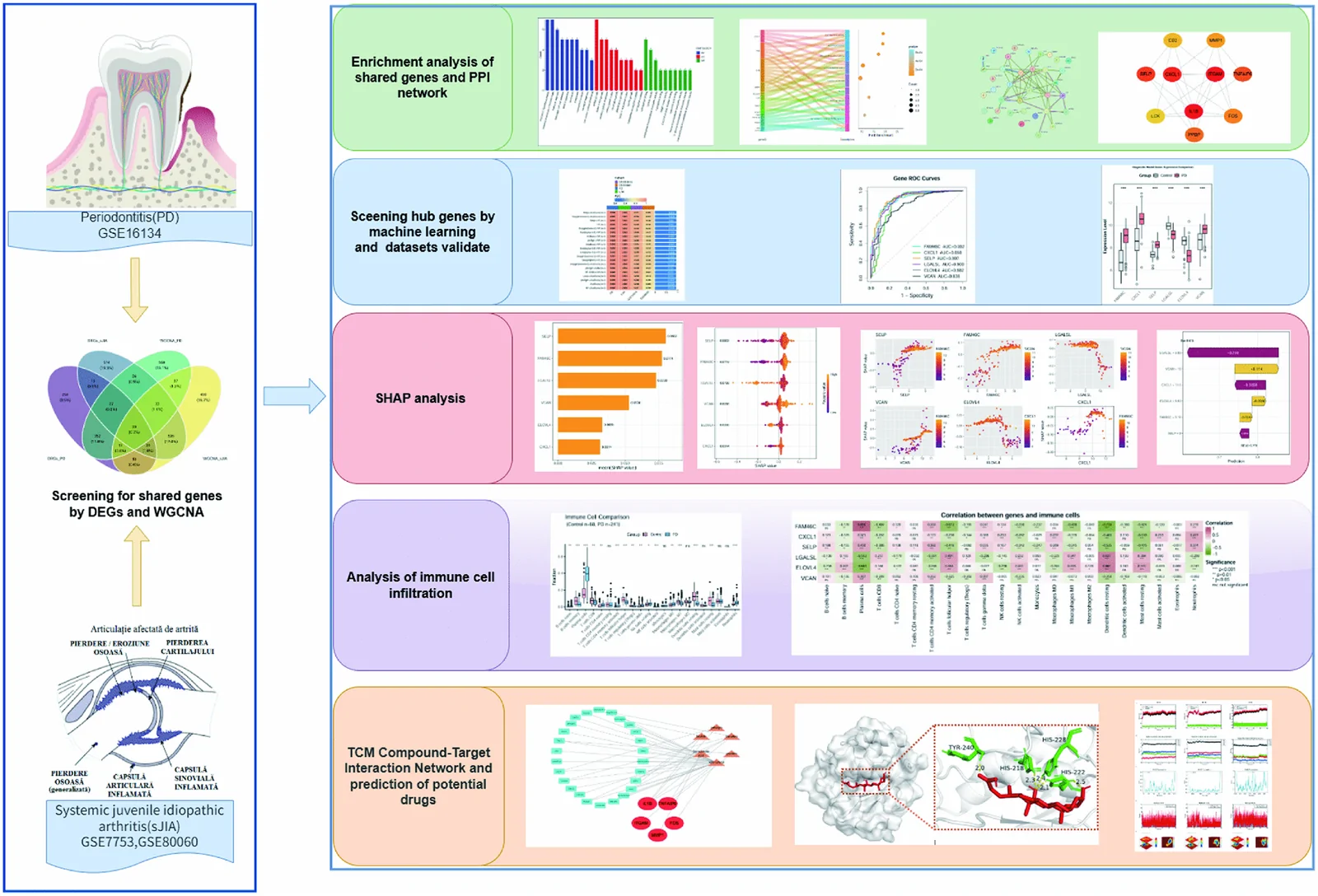
Flowchart of the study.

However, there is insufficient evidence to date linking PD and sJIA. This study employed bioinformatics to analyze data from GEO database to identify shared PD and sJIA genetic factors. The objective was to identify the genes that cause these two diseases, their immune characteristics, and the mechanisms that underlie them. In addition, we sought to identify potential therapeutic targets for both diseases. The overall workflow is as follows (Fig 1).

## 2. Result

### 2.1. Identification of Differentially Expressed Genes between PD and sJIA

**Fig 2:**
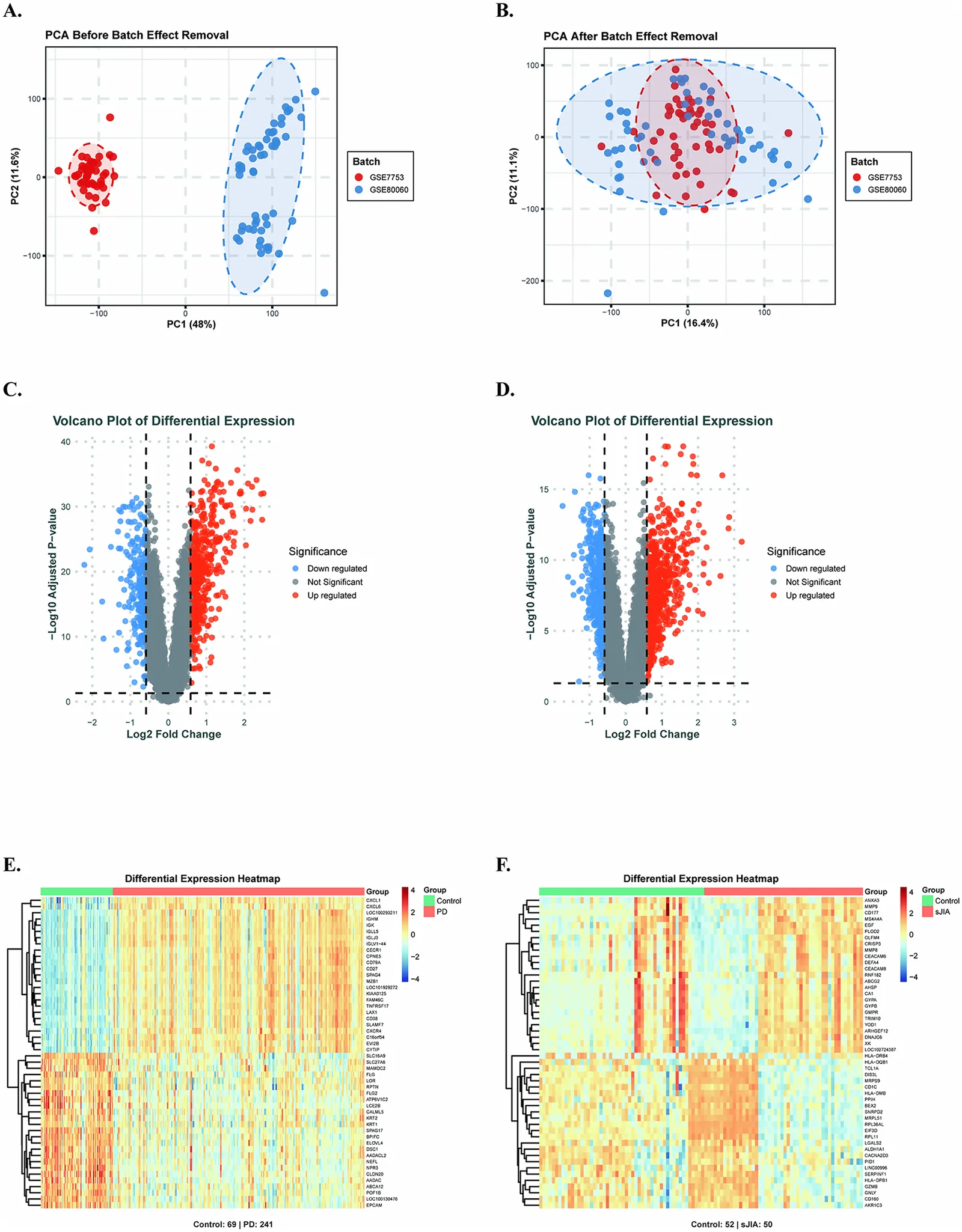
Identification of differentially expressed genes(DEGs). (A, B) PCA scatter plot shows distinct separation between GSE7753 and GSE80060 datasets before batch correction and after batch correction, indicating reduced batch effects. **(C, D)**Volcano plots depicting DEGs for datasets PD(GSE16134) and sJIA (GSE7753, GSE80060), respectively. Significantly upregulated genes were shown in red, significantly downregulated genes in blue, and non-significant genes in grey. **(E, F)** Heatmaps illustrating the expression patterns of representative significantly 25 upregulated and downregulated DEGs identified in PD (GSE16134) and sJIA (GSE7753, GSE80060) datasets.

In order to mitigate batch effects, we have consolidated the data sets GSE80060 and GSE7753, and we have also standardized the gene expression matrix resulting from the consolidation. PCA analysis of the constituent elements reveals that data with homogeneity demonstrate a greater degree of aggregation (Fig 2A and 2B**)**. As indicated by the analysis of differences in gene expression, 734 DEGs that are associated with PD were identified, with 491 up-regulated genes and 243 down-regulated genes demonstrated by the volcano **(**Fig 2C). The heatmap highlights 25 of the most significant DEGs, demonstrating increased as well as decreased (Fig 2E). In addition, the comprehensive screening for sJIA identified 1231 DEGs. The heatmap demonstrates the presence of 685 up-regulated genes and 546 down-regulated genes (Fig 2D). Moreover, the heatmap highlights 25 of the most significant DEGs, demonstrating increased as well as decreased (Fig 2F).

### 2.2. Weighted gene coexpression network analysis of PD and sJIA

**Fig 3:**
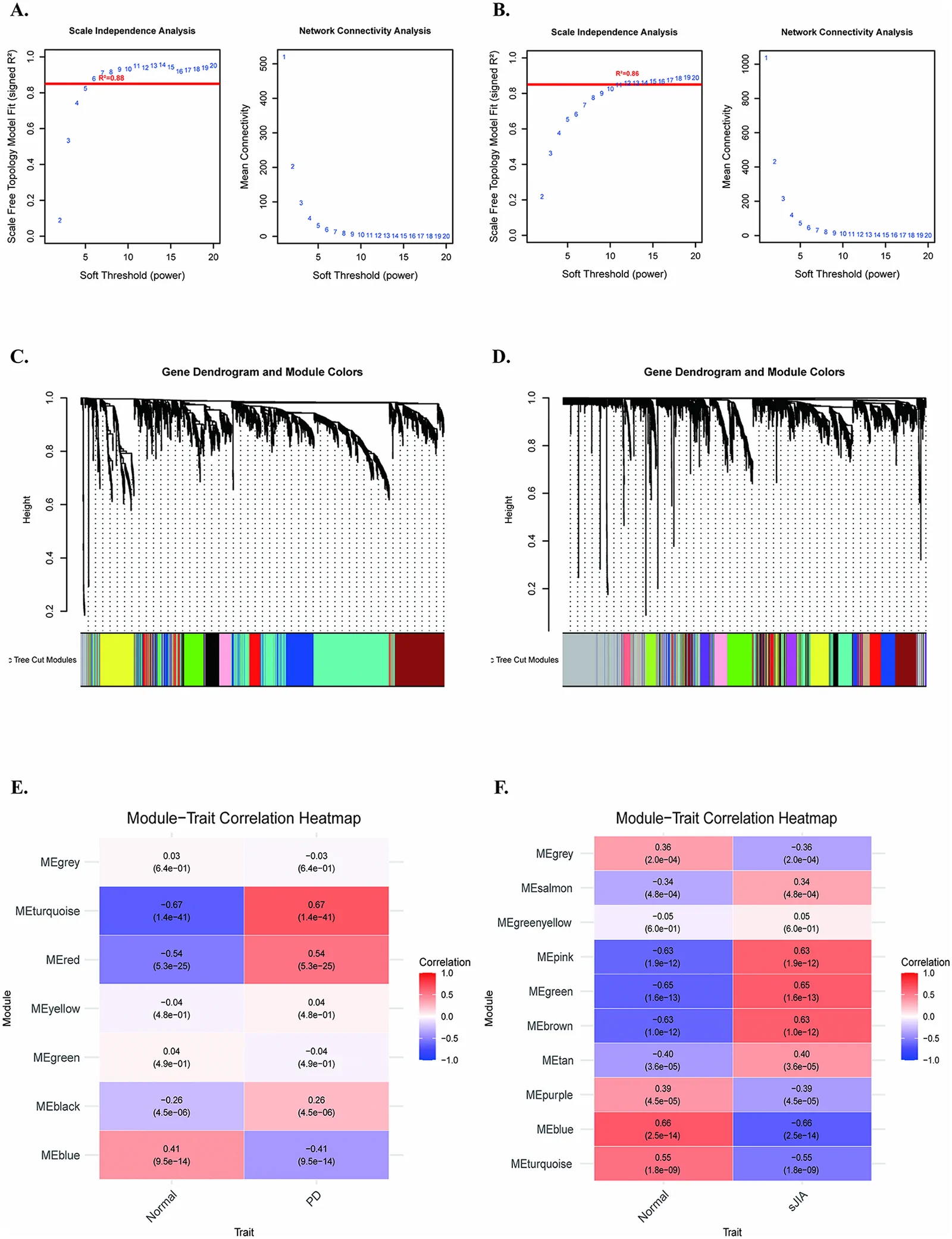
Weighted gene coexpression network analysis of PD and sJIA. (A, B) Analysis of the scale-free fit index and mean connectivity to determine the best soft-thresholding power in PD and sJIA. **(C, D)** Clustering dendrograms of genes in PD and sJIA. **(E, F)** Heatmap of the correlation analysis of module eigengenes with clinical phenotypes in PD and sJIA. Red color represents positive correlation and blue color represents negative correlation.

WGCNA was performed on the integrated dataset comprising both PD and sJIA datasets. To ensure robust module detection, we performed a systematic sensitivity analysis across independent datasets to determine the optimal gene filtering criterion. Based on this analysis, genes with a standard deviation(SD) > 0.5 across samples were retained for network construction, as this threshold achieved the best balance between retaining sufficient genes and maintaining stable module structures, whereas lower thresholds (SD = 0.3) introduced excessive noise and higher thresholds (SD ≥ 0.7) led to substantial loss of biological information (module counts sensitivity are shown in **Figure A in** S1 Fig **for PD, and Figure B in** S1 Fig **for sJIA**; the corresponding Jaccard similarity heatmaps are shown in **Figure C in** S1 Fig **for PD** and **Figure D in** S1 Fig **for sJIA**).Next, the optimal soft-thresholding powers were determined as 6 for PD and 12 for sJIA (Fig 3A and 3B). Subsequently, highly similar modules were merged based on eigengene network similarity, and the unassigned gray module (representing genes without clear co‑expression patterns) was excluded from further analysis (Fig 3C and 3D). This process yielded 6 effective co‑expression modules for the PD dataset and 9 for the sJIA dataset, respectively. Finally, the correlation between the modules and the traits must be determined. Within the PD framework, the turquoise modules demonstrate the strongest correlation with the PD traits (r = 0.67) (Fig 3E). In the case of sJIA, the pink, green and brown modules exhibited the highest positive correlations with the sJIA trait (r > 0.6) **(Fig 3F)**. The gene significance (GS) and module membership (MM) showed strong positive correlations, with correlation coefficients of r = 0.986 for the turquoise module (PD) (**Figure A in** S2 Fig), r = 0.607 for the pink module (**Figure B in** S2 Fig), r = 0.86 for the green module (**Figure C in** S2 Fig), and r = 0.965 for the brown module (sJIA) (**Figure D in** S2 Fig).

### 2.3. Enrichment analysis of PD and sJIA shared genes

**Fig 4:**
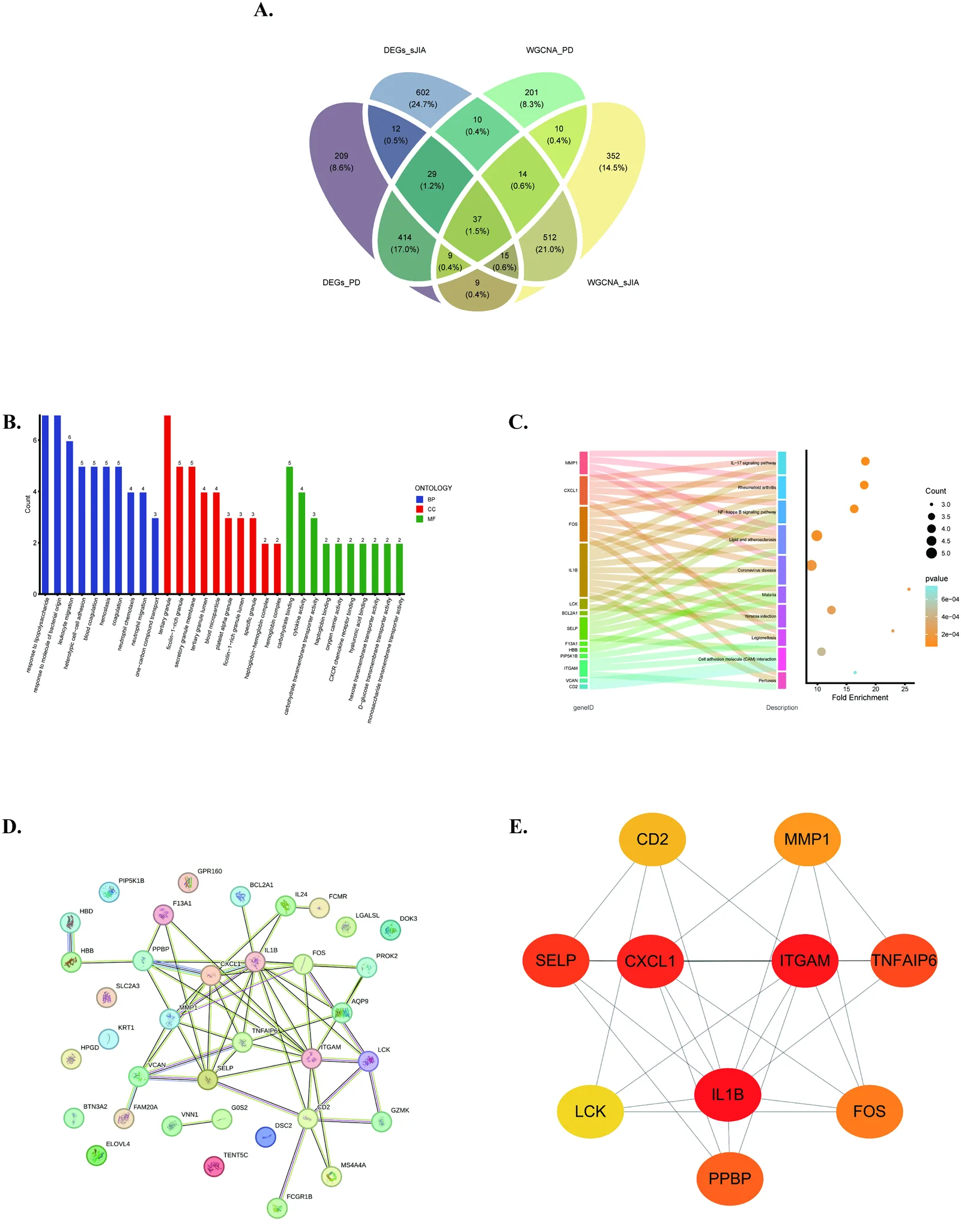
Functional enrichment and pathway enrichment of 37 shared genes. (A) Venn diagram of overlapping DEGs and gene module intersection between PD and sJIA. **(B)** Graph showing GO analysis of shared genes. **(C)** sankey+dot plot showing KEGG pathway analysis of shared genes. **(D)** PPI network of overlapping genes. **(E)** CytoHubba extracted the top 10 hub genes, ranked by MCC score, and these genes are highlighted in red with the MCC values indicated.

By intersecting the DEGs from PD and sJIA with the genes that were highly connected within each disease’s WGCNA modules, we identified 37 overlapping genes as initial candidates (Fig 4A)*.*Consensus WGCNA validation (Consensus TOM = min(TOM_PD, TOM_sJIA)) further demonstrated that these candidates possess significantly elevated consensus connectivity relative to random background genes (permutation test, n = 1000, p = 0.008**)**, supporting their status as conserved (S3 Fig).These genes are likely to play a pivotal role in the molecular mechanisms of PD and sJIA. Consequently, we initially conducted GO and KEGG enrichment analysis on these genes. The outcomes of the study indicate that a significant proportion of the genes are concentrated in pathways related to IL‑17 signaling pathway, Rheumatoid arthritis, NF‑kappa B signaling pathway and Lipid and atherosclerosis (Fig 4B and 4C). Subsequently, in order to identify additional genes with similar functions, 37 overlapping genes were entered into the String database (Fig 4D). Following the removal of independent genes, the CytoHubba MCC algorithm (Cytoscape software) was employed to identify the top 10 hub genes (Fig 4E).

### 2.4. Machine learning-based screening and validation of core diagnostic genes

**Fig 5:**
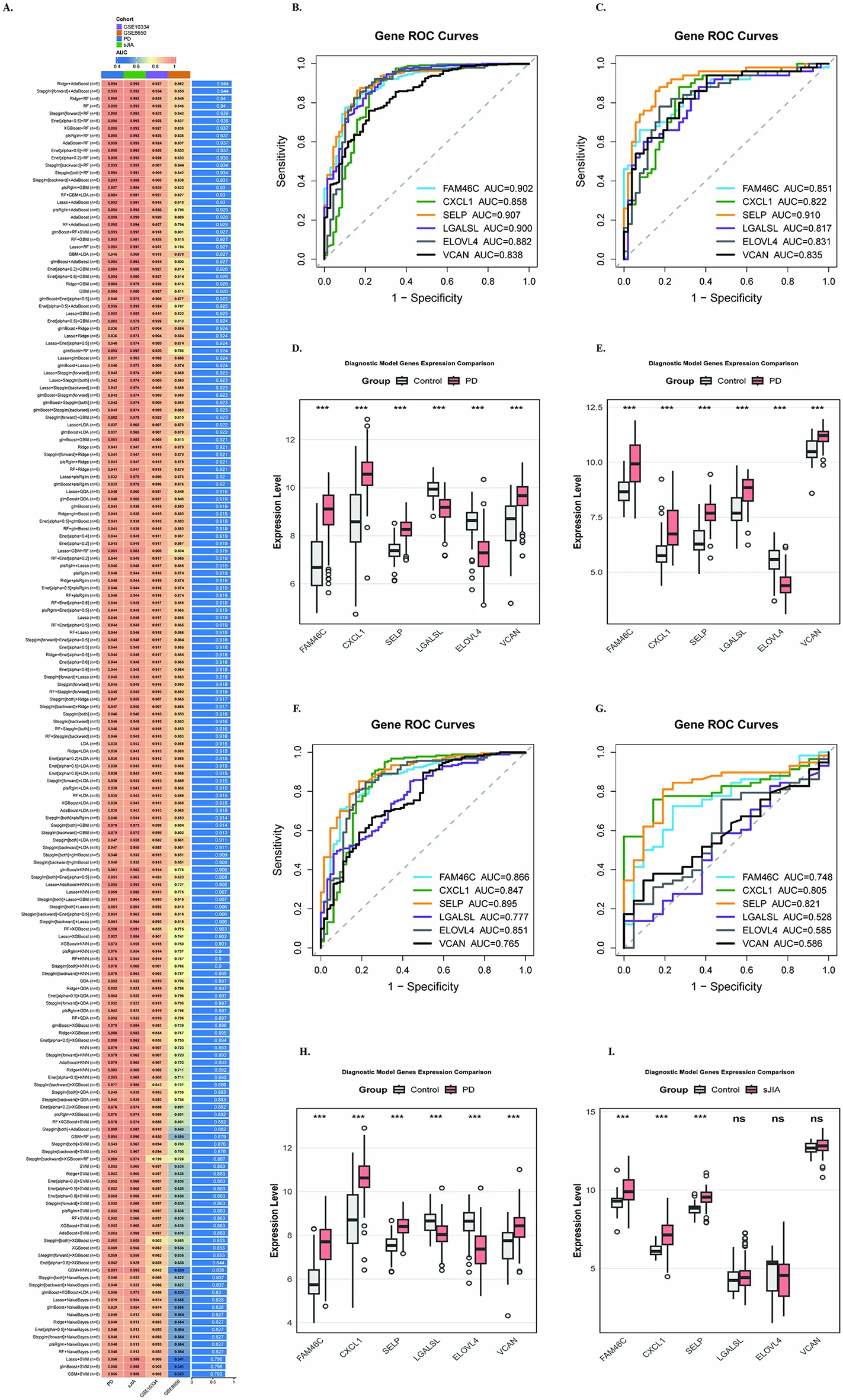
Machine learning-based screening and validation of core diagnostic genes. (**A)** A comparison of model performance is illustrated by the heatmap, which displays the AUC values for a variety of models across different cohorts. The left column of the figure corresponds to the models, while the right column indicates the AUC, with higher values indicating better performance. The utilization of colors serves to denote the provenance of each cohort. **(B, C)** ROC curve of 6 core diagnostic genes in the PD training dataset and the sJIA training dataset**. (D, E)** Expression of 6 core diagnostic genes in the PD training dataset and the sJIA training dataset. **(F, G)** ROC curve of 6 core diagnostic genes in the PD validation set and the sJIA validation set.**(H, I)** Expression of 6 core diagnostic genes in the PD validation set and the sJIA validation set.

Through comprehensive machine learning analysis of the 37 candidate targets, we constructed 175 predictive models to identify core shared genes involved in PD and sJIA. The Ridge + AdaBoost ensemble model demonstrated superior performance with the highest accuracy in both training and validation phases (Fig 5A),leading to the identification of six pivotal genes(*FAM46C, CXCL1, SELP, LGALSL, ELOVL4,* and *VCAN*). The diagnostic potential of these core genes in PD training dataset and sJIA training dataswt was evidenced by ROC curve analysis (AUC > 0.8, Fig 5B and 5C). A subsequent box-plot analysis revealed that, in the disease groups of the PD and sJIA training datasets, the expression levels of several genes, including *CXCL1, FAM46C, SELP* and *VCAN*, were consistently and significantly elevated, while the expression level of *ELOVL4* was consistently and significantly reduced. Additionally, *LGALSL* levels exhibited a downward trend in the PD disease group, while they exhibited an upward trend in the sJIA disease group (Fig 5D and 5E). In the validation set, AUC analysis of the ROC curve confirmed that these core genes exhibited good diagnostic performance in the PD validation set (AUC > 0.75, Fig 5F). Conversely, in the sJIA validation set, only *FAM46C, CXCL1,* and *SELP* exhibited good diagnostic performance, while the other genes exhibited poor diagnostic performance (AUC < 0.6, Fig 5G). In a similar vein, an examination of core gene expression levels in the validation set reveals that, in the PD validation set, the expression levels of the six core genes in the disease group exhibited concordance with those in the training set (Fig 5H). Conversely, in the sJIA validation set, no significant differences in the expression levels of *LGALSL, ELOVL4*, and *VCAN* were observed between the disease group and the control group (Fig 5I).

### 2.5. Model performance and SHAP-based feature interpretation

**Fig 6:**
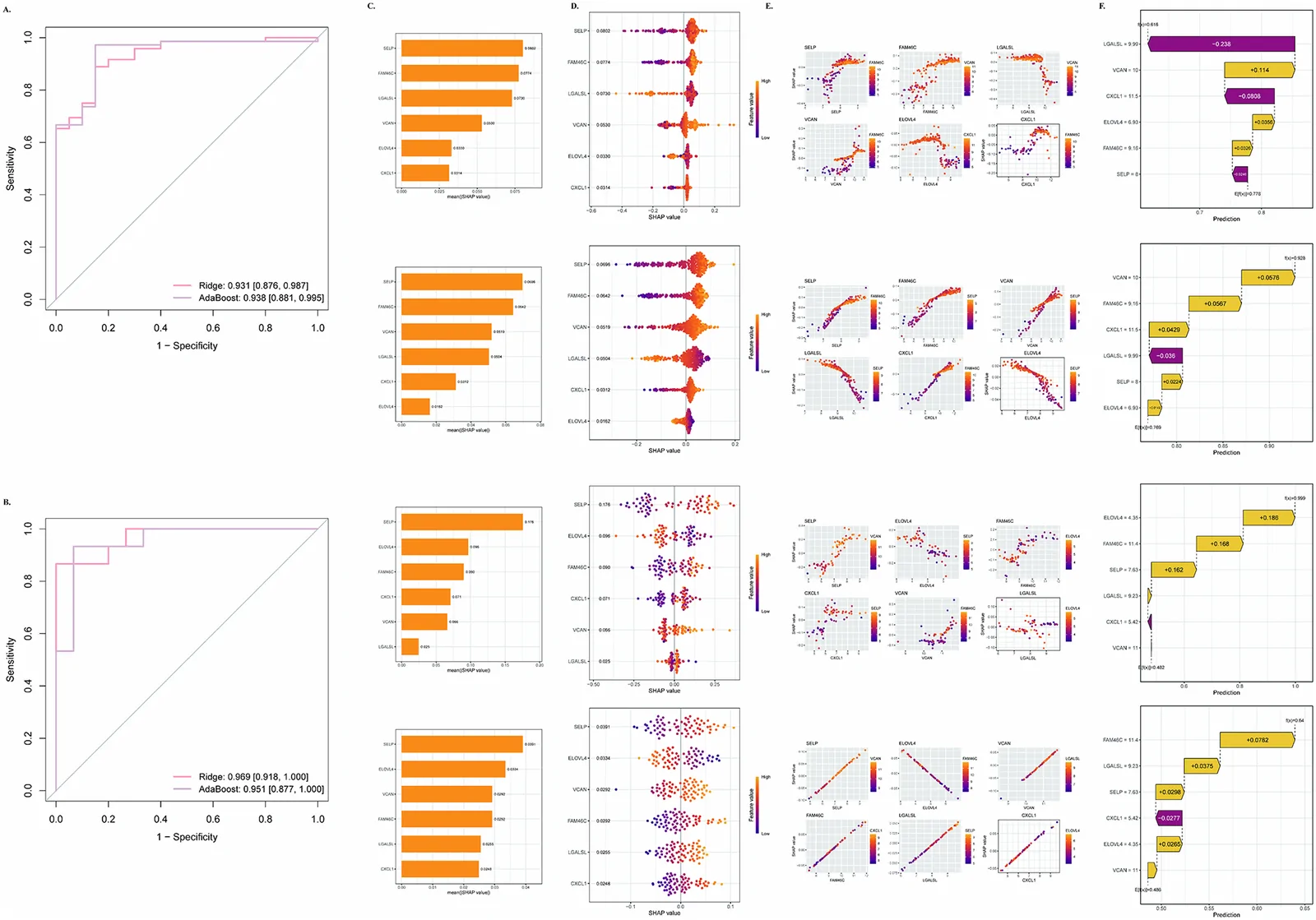
Model performance and SHAP-based feature interpretation. (**A, B)** Predicted area under the curve (AUC) values for each model in the PD dataset and the sJIA dataset.**(C)** Feature Importance Rankings in the Dataset-Model PD AdaBoost, PD Ridge, sJIA AdaBoost, and sJIA Ridge. **(D)** Violin plots for the datasets-models PD AdaBoost, PD Ridge, sJIA AdaBoost, and sJIA Ridge: The violin plots illustrate the distribution of gene expression under different conditions (width = data density, color = expression level)**. (E)** The dependence plots for the datasets-models PD AdaBoost, PD Ridge, sJIA AdaBoost, and sJIA Ridge: Scatter plots show SHAP values for key genes, indicating their impact on predictions**. (F)** The waterfall plots for the datasets-models PD AdaBoost, PD Ridge, sJIA AdaBoost, and sJIA Ridge:: SHAP summary plot shows gene contributions to predictions(Negative SHAP = lowering effect, positive = increasing effect).

We evaluated the predictive performance of Ridge and AdaBoost models on the PD and sJIA datasets using five-fold repeated cross‑validation with optimised hyperparameters (**see in** S1 Table). The ROC curveshows that both models demonstrated strong discriminatory power on both the PD and sJIA datasets (Fig 6A and 6B). To dissect the decision logic behind these models, we performed SHAP analysis across all four model–dataset combinations, examining global feature importance, directional consistency, and local prediction decomposition. The barplots of mean absolute SHAP values revealed that *SELP* ranked first in all four analyses, with values of 0.0802 (PD AdaBoost), 0.0696 (PD Ridge), 0.162 (sJIA AdaBoost), and 0.0391 (sJIA Ridge) – the only gene to maintain a consistent top‑position across models and datasets (Fig 6C). In PD AdaBoost, *SELP* (0.0802) and *CXCL1* (0.0774) were nearly tied (difference 0.0028), whereas in sJIA AdaBoost, *SELP* far exceeded the second‑ranked *ELOVL4*(0.112), indicating a disease‑context‑dependent amplification. *FAM46C* ranked second in PD Ridge (0.0642), *ELOVL4* ranked second in both sJIA models, while *VCAN* and *LGALSL* occupied intermediate positions across analyses. The beeswarm plots showed that *SELP* and *CXCL1* exhibited perfectly consistent and opposite directional patterns across all four combinations: higher *SELP* expression uniformly associated with positive SHAP values (risk‑driving), whereas higher *CXCL1* expression consistently linked to negative SHAP values (protective) – a reproducibility across algorithms and diseases that strongly supports their biological robustness (Fig 6D). In contrast, *FAM46C, ELOVL4, VCAN* and *LGALSL* showed mixed or model‑dependent directions. The waterfall plots illustrated local prediction decomposition for representative samples; in the sJIA Ridge example, the baseline probability E[f(x)] = 0.486 was raised to f(x) = 0.64, with *FAM46C* contributing the largest positive increment (+0.0782), followed by *LGALSL* (+0.0375), *SELP* (+0.0298) and *ELOVL4* (+0.0265), while *CXCL1* exerted a negative effect (−0.0277) – demonstrating that globally top‑ranked genes are not necessarily the dominant drivers for every individual patient (Fig 6F). The dependence plots further visualised SHAP‑expression relationships and suggested potential non‑linear interaction effects, particularly the amplified *SELP* contribution in sJIA AdaBoost (Fig 6E).

Collectively, these results identify *SELP* as the most robust cross‑model, cross‑dataset predictor with a stable risk‑driving direction, *CXCL1* as a universally protective factor, and *FAM46C, ELOVL4, VCAN* and *LGALSL* as context‑dependent contributors whose relative importance shifts with disease type and modelling framework.

### 2.6. Immune cell infiltration and its correlation with shared core genes

**Fig 7:**
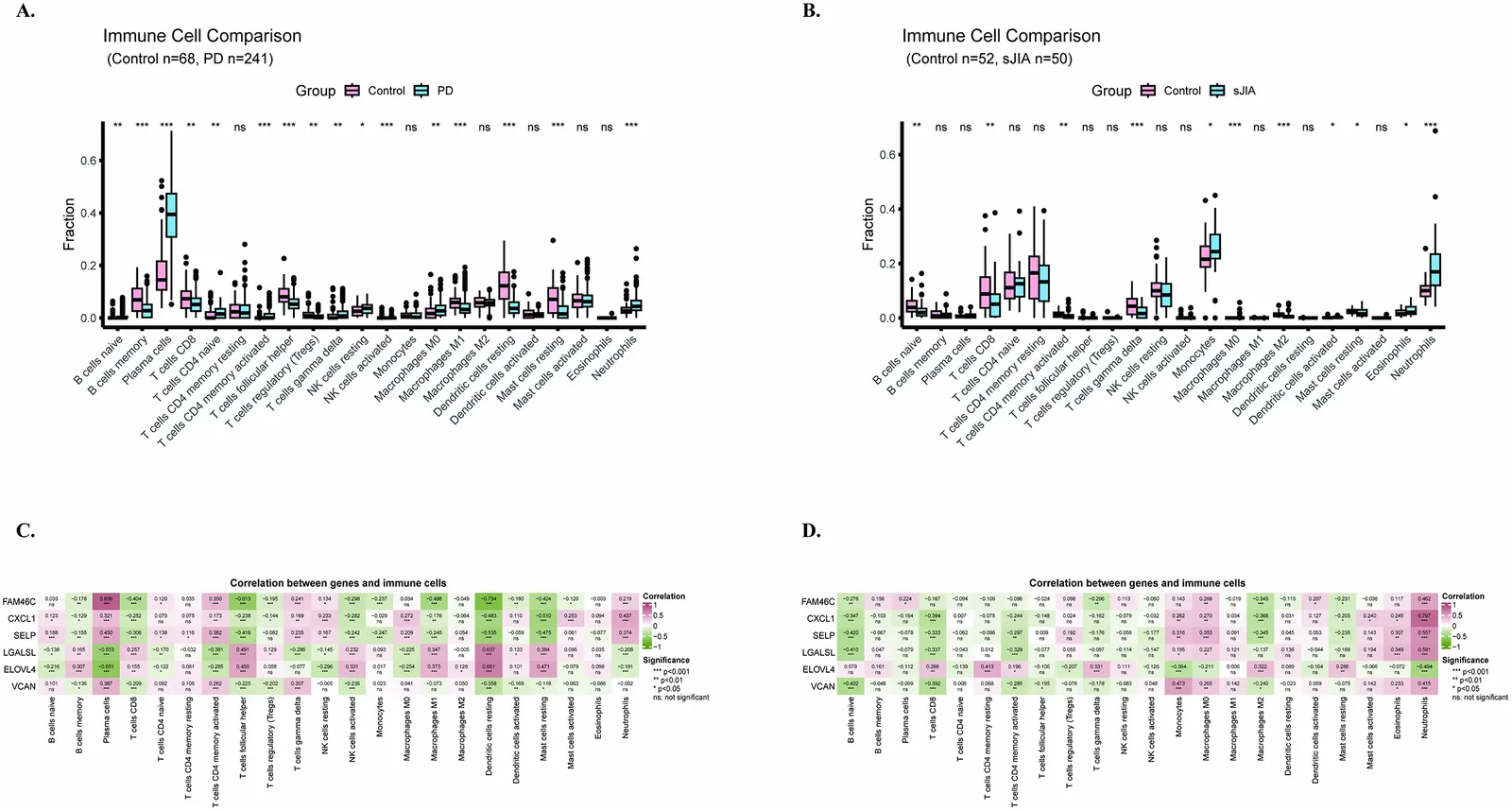
Immune cell infiltration and its correlation with shared core genes. (**A)** showing the distribution of 22 immune cells in the PD training sample. **(B)** showing the distribution of 22 immune cells in the sJIA training sample. **(C)** Heatmap showing the correlation between six core genes and immune cell infiltration in PD training samples. **(D)** Heatmap showing the correlation between six core genes and immune cell infiltration in sJIA training samples.

Our CIBERSORT analyses were performed independently for each disease against its own tissue‑matched control, without any direct cross‑tissue statistical comparison. In PD gingiva, we observed a significant elevation of neutrophils and a relative reduction of CD8 ⁺ T cells, while in sJIA peripheral blood, neutrophils were also elevated and CD8 ⁺ T cells were similarly reduced (Fig 7A and 7B). Correlation analysis of six core genes (*FAM46C, CXCL1, SELP, LGALSL, ELOVL4, VCAN*) with immune cell fractions revealed that *CXCL1* and *SELP* consistently correlated positively with neutrophils and negatively with resting mast cells in both datasets, whereas *FAM46C* showed positive association with plasma cells. Notably, *LGALSL* exhibited opposite correlations with neutrophils between the two tissues (negative in PD, positive in sJIA), while *ELOVL4* positively correlated with M2 macrophages in both diseases, with a stronger correlation in sJIA. Additionally, *VCAN* showed a significant positive correlation with neutrophils only in sJIA, whereas no such relationship was observed in PD (Fig 7C and 7D). These patterns underscore both shared immune–gene interactions and tissue-specific regulatory differences across the two inflammatory diseases.

The observed overlaps and differences in gene–cell correlations may partly reflect tissue‑specific signals rather than a genuine shared pathogenic mechanism. Moreover, the lack of publicly available same‑tissue‑type datasets (PD blood or sJIA synovium) precludes direct matched validation. Therefore, all cross‑disease inferences presented here are hypothesis‑generating, not confirmatory, and require future validation in appropriately matched tissue cohorts.

### 2.7. Identification of candidate drugs based on hub genes and molecular docking analysis

**Fig 8:**
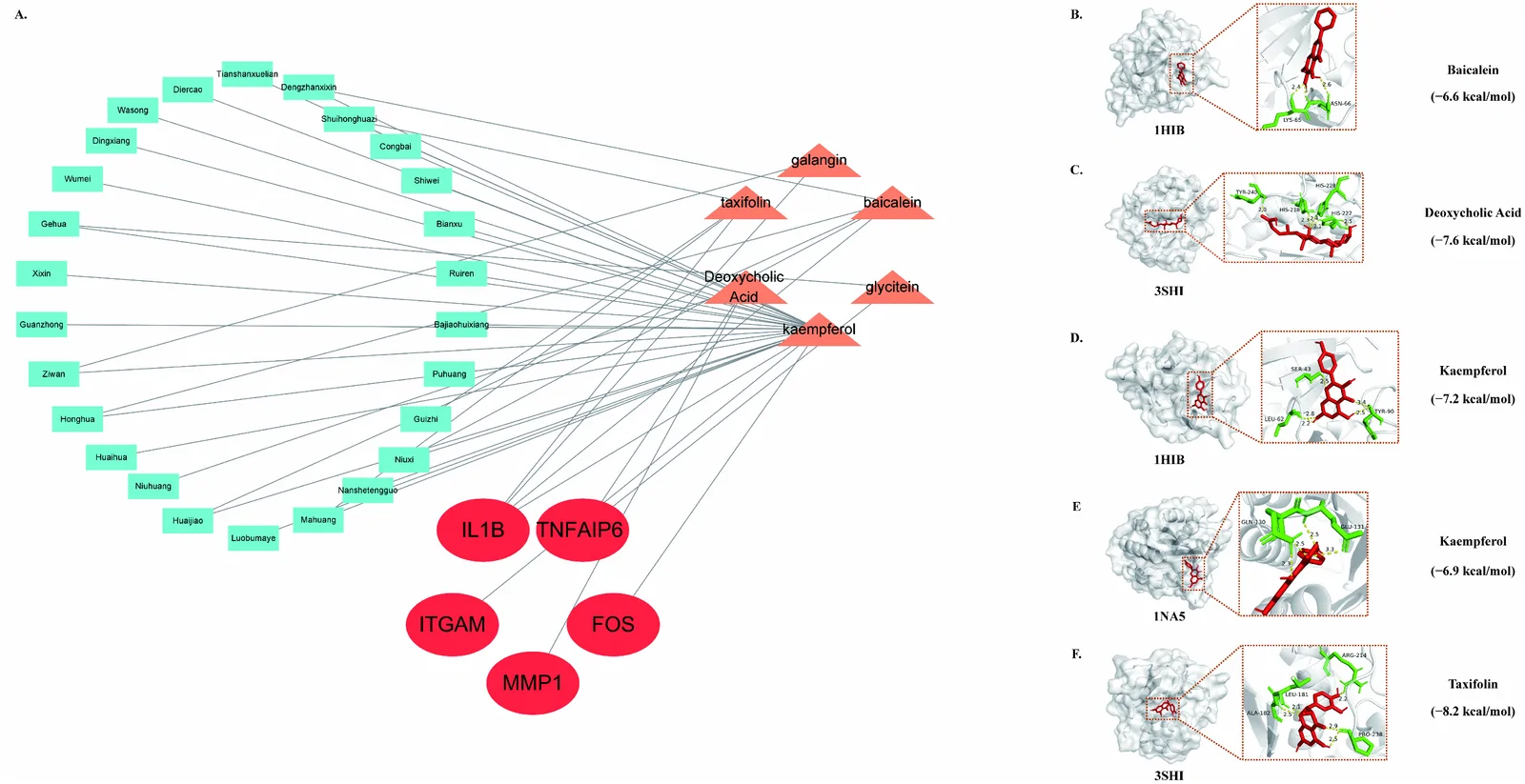
Hub Gene-Based Reverse Prediction of Traditional Chinese Medicines. (**A)** Traditional Chinese Medicine-Compound-Target PPI Network Diagram. **(B)** Molecular Docking Diagram of 1HIB(*IL1B*) and baicalein. **(C)** Molecular Docking Diagram of 3SHI(*MMP1*) and deoxycholic acid. **(D)** Molecular Docking Diagram of 1HIB(*IL1B*) and kaempferol. (**E)** Molecular Docking Diagram 1NA5 (*ITGAM*) and kaempferol. (**F)** Molecular Docking Diagram 3SHI(*MMP1*) and taxifolin.

We have developed a network of crucial target genes for traditional Chinese medicine (TCM) compounds by integrating the CTD and TCMSP databases (Fig 8A). This network highlights the central role of the target genes *IL1B, MMP1*, and *ITGAM* in the regulation of this network. The molecular interactions between the selected compounds and their respective target proteins (PDB IDs: 1HIB for *IL1B*, 3SHI for *MMP1*, and 1NA5 for *ITGAM*) were determined by molecular docking using AutoDock Vina [11]. The binding affinities were calculated as −6.6 kcal/mol for baicalein–*IL1B,* −7.6 kcal/mol for deoxycholic acid–MMP1, −6.9 kcal/mol for kaempferol–*ITGAM*, −7.2 kcal/mol for kaempferol–IL1B, and −8.2 kcal/mol for taxifolin–MMP1 (Fig 8B-8F). Previous studies have established reference thresholds for interpreting AutoDock binding energies: a binding energy “significance” threshold of −7.0 kcal/mol has been identified to distinguish strongly binding ligands from weak or nonspecific interactions, and a binding energy below −6.0 kcal/mol has been proposed as the minimum threshold for drug development candidates [12,13]. Based on these criteria, deoxycholic acid–*MMP1* (−7.6 kcal/mol), kaempferol–*IL1B* (−7.2 kcal/mol), and taxifolin–*MMP1* (−8.2 kcal/mol) all fall below the −7.0 kcal/mol significance threshold, indicating strong predicted binding affinity for their respective targets, while baicalein–*IL1B* (−6.6 kcal/mol) and kaempferol–ITGAM (−6.9 kcal/mol) fall below the −6.0 kcal/mol threshold, suggesting favourable binding potential as well.

### 2.8. Molecular dynamics simulation and binding stability assessment

Although semi‑flexible docking provides initial binding poses, it cannot account for protein flexibility, temperature, pressure, or solvent effects. To further investigate the dynamic stability of the protein–ligand interactions, we performed 100‑ns MD simulations on three key complexes: Kaempferol–*IL1B* (PDB: 1HIB), Kaempferol–*ITGAM* (PDB: 1NA5), and Deoxycholic Acid–*MMP1* (PDB: 3SHI). Multiple parameters were analysed to evaluate the reliability of each binding mode.

**Fig 9:**
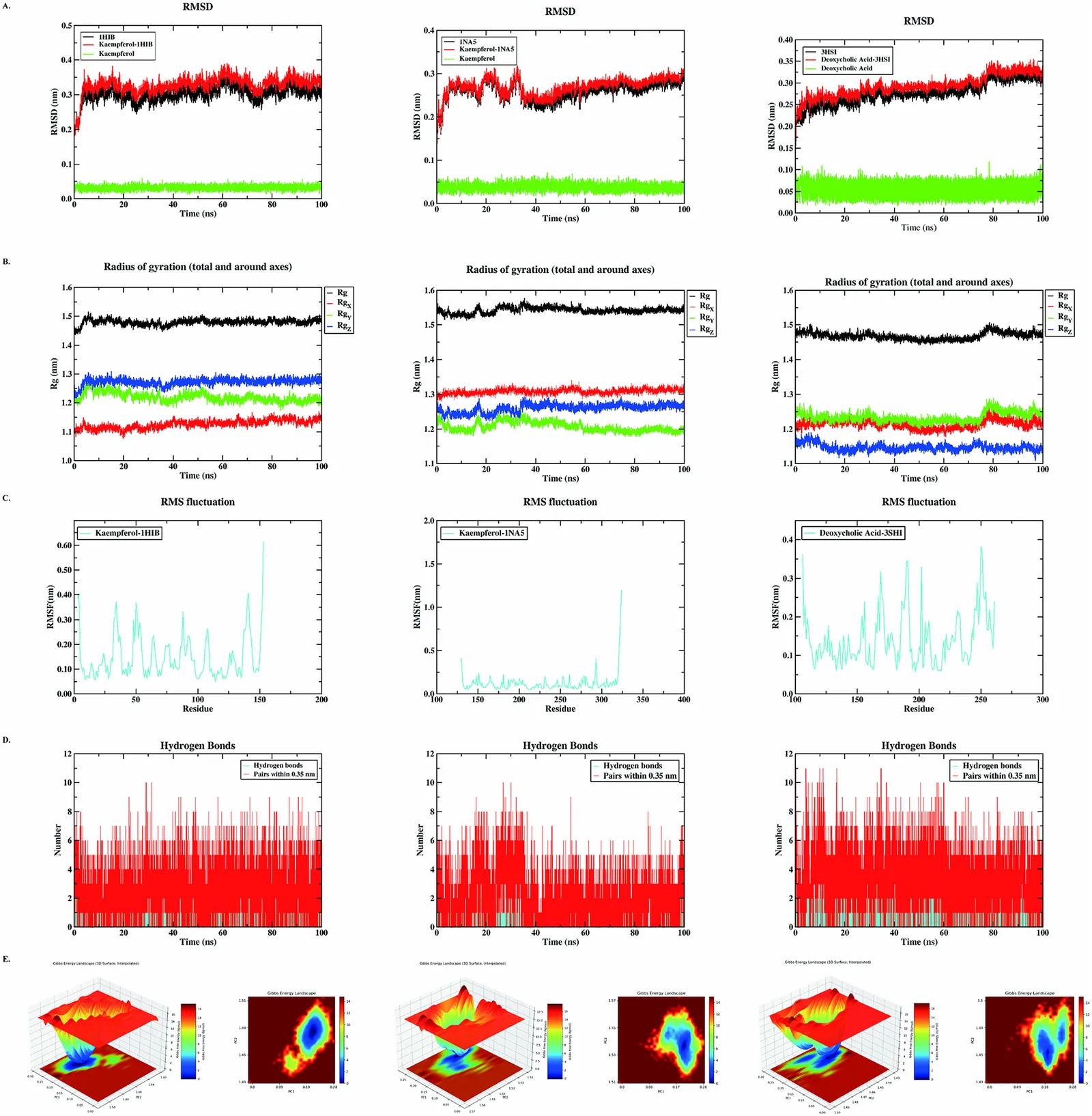
Molecular dynamics (MD) simulation analyses of three ligand–protein complexes (Kaempferol–*IL1B*, Kaempferol–*ITGAM*, and Deoxycholic Acid–*MMP1*) over 100 ns. (**A)** Root-mean-square deviation (RMSD) of the protein backbone. **(B)** Radius of gyration (Rg). **(C)** Root-mean-square fluctuation (RMSF) per residue. (**D)** Hydrogen bonds within 0.35 nm. **(E)** Gibbs free energy landscapes (3D surface and 2D contour).

The root-mean-square deviation (RMSD) of the protein backbone was monitored to assess equilibration (Fig 9A). Kaempferol–1HIB (red line) reached equilibrium and stabilised at approximately 0.30–0.35 nm, which is slightly higher than that of its apo-protein (1HIB, black line). Kaempferol–1NA5 (red line) stabilised at approximately 0.25–0.30 nm, also slightly higher than its apo-protein (1NA5, black line). Deoxycholic Acid–3SHI (red line) stabilised at approximately 0.25–0.30 nm, again slightly higher than its apo-protein (3SHI, black line). Although the RMSD values of all three complexes slightly exceed the conventional 0.25 nm threshold, they remain stable with no upward drift throughout the simulation, indicating that ligand binding preserves the overall structural integrity, despite a marginal increase in backbone RMSD relative to the unbound proteins.

The radius of gyration reflects overall complex compactness (Fig 9B). Kaempferol–1HIB maintained a stable Rg at approximately 1.48 nm; Kaempferol–1NA5 at approximately 1.54 nm; and Deoxycholic Acid–3SHI at approximately 1.48 nm, all with minimal fluctuations throughout the simulation. The absence of abrupt increases in Rg confirms that ligand binding did not induce protein unfolding or significant expansion.

Per‑residue flexibility was evaluated via RMSF (Fig 9C). For Kaempferol–1HIB, most residues fluctuated below 0.12 nm, with only residue 150 (C‑terminus) showing higher flexibility (~0.62 nm). For Kaempferol–1NA5, the vast majority of residues remained below 0.12 nm, with only residue 1 (N‑terminus) showing higher fluctuation (~0.42 nm). For Deoxycholic Acid–3SHI, most residues fluctuated below 0.20 nm, with residues 100, 190, and 210 showing slightly higher values (~0.35–0.38 nm). Binding pocket residues in all complexes showed consistently low fluctuations, confirming rigid and specific binding sites.

All complexes maintained stable hydrogen bonds within 0.35 nm throughout the simulation (Fig 9D). Kaempferol–1HIB maintained 0–3 hydrogen bonds with 0–11 stable pairs. Kaempferol–1NA5 maintained 0–2 hydrogen bonds with 0–10 stable pairs. Deoxycholic Acid–3SHI exhibited 0–6 hydrogen bonds and 0–11 stable pairs; however, no continuous increasing trend was observed, indicating that polar interactions remained dynamic rather than progressively strengthened over time.

Gibbs free energy landscapes based on principal components (PC1/PC2) revealed a single, narrow, deep energy basin for all three complexes (Fig 9E), with minimum free energy approaching 0 kJ/mol. This indicates that each system fluctuated within a single thermodynamically favourable conformational state without transitioning to other metastable states, confirming the stability and selectivity of the binding modes.

Multi‑parameter MD analyses consistently demonstrate that all three complexes exhibit good conformational stability and specific binding under dynamic physiological conditions, providing robust computational validation for the docking predictions.

## 3. Discussion

The precise mechanism underlying sJIA remains to be elucidated; it does not conform to the typical characteristics of an autoimmune disease and is frequently associated with systemic inflammatory manifestations [5]. Periodontal disease is a highly prevalent, multifactorial, chronic condition, and recent reports have indicated its association with a range of other autoimmune diseases [14–16]. The latent mechanisms underlying these diseases remain to be fully elucidated. In recent years, bioinformatics has emerged as a novel method to analyze and disclose the physiological mechanisms of various diseases. In this study, bioinformatics was used to discern the shared diagnostic biomarkers of PD and sJIA, offering novel approaches to prevent and treat these diseases.

In this study, we integrated GEO datasets for PD and sJIA and performed differential expression analysis and WGCNA, identifying 734 DEGs in PD (491 up, 243 down) and 1,231 in sJIA (685 up, 546 down), with the turquoise module (PD, r = 0.67) and pink, green and brown modules (sJIA, r > 0.6) showing the strongest trait correlations. Intersection of DEGs and WGCNA hub genes yielded 37 shared candidates, validated by consensus WGCNA (p = 0.008), which were significantly enriched in IL‑17, NF‑κB, rheumatoid arthritis, and lipid atherosclerosis pathways. PPI network analysis identified top‑ten hub genes, and subsequent machine learning analysis of 175 predictive models identified the Ridge + AdaBoost ensemble as the best‑performing combination, which selected six core diagnostic genes (*FAM46C, CXCL1, SELP, LGALSL, ELOVL4,* and *VCAN*), with ROC analysis confirming good diagnostic performance (AUC > 0.8 in training, > 0.75 in PD validation). SHAP interpretation highlighted *SELP* as the most robust cross‑model, cross‑dataset risk predictor, while *CXCL1* consistently showed protective effects. Both diseases share neutrophil elevation, CD8 ⁺ T reduction, and conserved correlations, including *CXCL1* and *SELP* positively with neutrophils and negatively with resting mast cells, and *FAM46C* positively with plasma cells, while *LGALSL* displays opposite neutrophil associations between the two conditions. Drug‑target network analysis pinpointed *IL1B, MMP1*, and *ITGAM* as core targets. Molecular docking of five compound–target pairs yielded binding affinities of −6.6 kcal/mol (baicalein–*IL1B*), −7.6 kcal/mol (deoxycholic acid–*MMP1*), −6.9 kcal/mol (kaempferol–ITGAM), −7.2 kcal/mol (kaempferol–*IL1B*), and −8.2 kcal/mol (taxifolin–*MMP1*); among these, the kaempferol–*IL1B*, kaempferol–*ITGAM*, and deoxycholic acid–*MMP1* complexes were selected for 100‑ns MD simulations, which confirmed stable and specific binding through RMSD, Rg, RMSF, hydrogen bond, and free energy landscape analyses.

It is important to acknowledge that all data used in this study derive from bulk transcriptomic profiling. Such datasets are inherently affected by both technical and biological biases [17]. First, bulk tumour samples represent mixed transcriptional signals from multiple cell types (malignant cells, immune cells, stromal cells, etc.), which may obscure cell type specific gene activity [17]. Second, the differentially expressed genes identified here reflect only transcriptional correlations; they cannot be interpreted as causal or functionally driving events—a highly expressed gene is not necessarily a driver of tumour progression, and conversely, moderately or lowly expressed genes may still play critical roles [18]. Third, mRNA expression does not always correlate with protein abundance; thus, our transcriptome level findings require further validation at the protein level and through functional experiments [18]. Consequently, our conclusions should be viewed as exploratory candidate screening results, and their translational potential awaits verification in prospective cohort studies and experimental assays.

All *SELP*‑related findings—including its top‑ranked SHAP importance across models and datasets, consistent risk‑driving direction, good diagnostic performance (AUC > 0.8), positive correlations with neutrophils and M0 macrophages, and enrichment in lipid atherosclerosis pathways—are derived solely from computational re‑analysis of public GEO datasets and lack any experimental validation (qPCR, Western blot, gene perturbation, animal models, or clinical samples). Although these statistical associations are consistent with the known role of P‑selectin in leukocyte rolling and thrombo‑inflammation [19,20] and with reports linking periodontal pathogens to the P‑selectin/PSGL‑1 axis [21], our study provides no direct evidence of upstream regulators, downstream effectors, or causal involvement in specific signalling cascades; pathway enrichment only indicates statistical over‑representation and cannot establish mechanistic hierarchy. Establishing causality would require systematic functional experiments (e.g., knockout/overexpression, inhibitor rescue, animal models), as exemplified by studies that validated NF‑κB pathway engagement through gene perturbation and molecular assays rather than inference alone [22,23]. Likewise, we make no claim that *SELP* is a therapeutic target, as any speculation on treatment efficacy would be premature without target engagement, cellular, and in vivo validation.

*CXCL1* (C‑X‑C motif chemokine ligand 1), also known as GRO‑α, is a CXC chemokine that functions as a chemotactic factor for neutrophils via its receptor *CXCR2* [24]. Our computational findings—consistent upregulation in both PD and sJIA, good diagnostic performance (AUC > 0.75), and robust SHAP importance (ranked second in PD AdaBoost with a consistently negative/protective direction)—are derived entirely from bioinformatic analyses and lack experimental corroboration. The observed pathway enrichment (IL‑17, NF‑κB, Rheumatoid arthritis, lipid atherosclerosis) and positive correlations with M0 macrophages and neutrophils are consistent with established roles of *CXCL1* in inflammatory diseases. In periodontitis, *CXCL1* is a key mediator of neutrophil recruitment and alveolar bone destruction. Mechanistically, periodontal pathogens like *P. gingivalis* trigger the integrated stress response (ISR) in gingival keratinocytes, upregulating *CXCL1* and promoting neutrophil infiltration [25]. Inflammation‑challenged Gli1 ⁺ mesenchymal stem cells release extracellular vesicles bearing *CXCL1* on their surface, which primes aberrant neutrophils via the *CXCL1–CXCR2* axis [26]. Insulin enhances lipopolysaccharide‑induced *CXCL1* production in gingival fibroblasts through Akt and NF‑κB activation, and targeted overexpression of *CXCL1* in the gingiva normalizes neutrophil recruitment and prevents bone loss in mouse models [27]. In rheumatoid arthritis—a disease immunologically related to sJIA—*CXCL1* is upregulated in the blood and synovial fluid of patients [28,29]. *CXCL1* promotes cyclooxygenase‑2 (COX‑II) expression in rheumatoid arthritis synovial fibroblasts via *CXCR2*, PLC, PKC, and NF‑κB signalling, and contributes to IL‑6 expression through *CXCR2*, c‑Raf, MAPK, and AP‑1 pathways [28,29]. Furthermore, mast cells and macrophages both produce *CXCL1/CXCL2* to control the early stage of neutrophil recruitment during tissue inflammation [30]. However, these associations do not establish a causal link or define the hierarchical position of *CXCL1* within these signalling cascades; pathway over‑representation only reflects statistical co‑occurrence and cannot be interpreted as mechanistic evidence. Establishing any functional role for *CXCL1* in the shared pathology of PD and sJIA would require systematic gene perturbation, rescue assays, and in vivo models. We make no claim that *CXCL1* constitutes a therapeutic target, as any such speculation would be premature without target validation and efficacy studies.

In summary, our computational results prioritise *SELP* and *CXCL1* as candidate genes that may jointly participate in the shared inflammatory–vascular pathology between PD and sJIA. However, all findings are purely exploratory and lack experimental validation; they do not establish mechanism or therapeutic potential. Rigorous functional studies are required to test these hypotheses. While our cross cohort computational validation demonstrates generalizability across independent transcriptomic datasets, future studies are warranted to validate the 2 gene signature using orthogonal methods (e.g., qRT PCR, protein level assays) and in prospective clinical cohorts

The immune microenvironment of gingival mucosa differs profoundly from that of peripheral blood. Gingival tissue is continuously exposed to oral microbiota and physical barriers, while peripheral blood reflects systemic immune status. Thus, the observed overlap in DEGs between these two datasets may conflate true disease‑relevant signals with tissue‑specific transcriptional programmes. With the current design, we cannot disentangle these two sources of variation. Consequently, our ‘shared gene’ findings are hypothesis‑generating and should not be interpreted as confirmatory evidence of a unified molecular pathway.

Our immune infiltration analyses reveal a convergent inflammatory landscape in both PD gingiva and sJIA peripheral blood, marked by elevated neutrophils and reduced CD8 ⁺ T cells. The neutrophil accumulation aligns with the established role of neutrophils as first-line effectors in both local mucosal infection (PD) and systemic autoinflammation (sJIA), while the concurrent CD8 ⁺ T‑cell decrease suggests a shared defect in adaptive cytotoxic surveillance, which may facilitate chronicity by impairing clearance of pathogenic stimuli [31–33]. The conserved positive correlations of CXCL1 and SELP with neutrophils, together with their negative associations with resting mast cells, point to a coordinated chemotactic–adhesive axis that not only recruits neutrophils but also modulates mast cell homeostasis, potentially linking innate activation with allergic-type pathways [21,34]. Simultaneously, the consistent FAM46C–plasma cell correlation implies that humoral immunity is engaged in both diseases, possibly reflecting a common B‑cell–driven component that sustains local and systemic inflammation via autoantibody or immune-complex formation [35]. Beyond these shared features, the disease‑dependent divergence of LGALSL–neutrophil correlations (negative in PD, positive in sJIA) highlights that even the same gene can exert opposing effects on neutrophil trafficking depending on tissue context—a phenomenon that may be explained by differential post-translational modifications or competing ligand-receptor interactions within the local microenvironment [36]. The stronger ELOVL4–M2 macrophage association in sJIA suggests that systemic lipid metabolic signals preferentially drive alternative macrophage polarization in peripheral blood, whereas in the gingival mucosa, this axis is partially overridden by microbial products and barrier-derived cytokines that favor M1-dominant responses [37]. The VCAN–neutrophil correlation uniquely observed in sJIA further supports a systemic adhesion–migration network that may be less relevant in oral tissues where neutrophil trafficking is dominated by integrins rather than versican-mediated pathways [38]. Collectively, these patterns are consistent with a dual-layered immunological framework: a core shared inflammatory backbone involving neutrophil–mast cell–plasma cell circuits, overlaid with tissue‑specific regulatory modules that rewire gene–immune cell interactions according to local architecture (mucosal vs. vascular) and systemic inflammatory tone (acute vs. chronic). This framework aligns with the concept of “inflammatory continuum” across barrier and systemic diseases, where common effector pathways are fine-tuned by tissue-resident stromal and immune niches [39]. However, these computational inferences remain correlative and hypothesis‑generating; they do not establish causal hierarchy. Functional validation—through cell-type-specific knockouts, neutralizing antibodies, or tissue-engineered models—is urgently needed to dissect whether the observed commonalities and divergences represent true pathogenic drivers or merely secondary epiphenomena of inflammation.

From the five docking pairs, we selected Kaempferol–*IL1B*, Deoxycholic Acid–*MMP1*, and Kaempferol–*ITGAM* for 100‑ns MD simulations based on four considerations: representative binding affinities covering moderate to strong ranges; functional diversity of targets—a pro‑inflammatory cytokine *(IL1B*), a matrix‑degrading enzyme (*MMP1*), and an immune adhesion molecule *(ITGAM*)—covering key nodes in the inflammatory cascade; compound structural variety (flavonoid vs. bile acid) to evaluate generalisability; and computational resource allocation to balance validation depth and coverage breadth. MD multi‑parameter analyses (RMSD, Rg, RMSF, hydrogen bonding networks, and free energy landscapes) confirmed good conformational stability and specific binding for all three complexes, providing robust computational support beyond binding energy alone.

Kaempferol is a natural flavonoid with well‑documented anti‑inflammatory properties [40], while IL‑1β is a master pro‑inflammatory cytokine central to both periodontitis and sJIA pathogenesis, driving neutrophil recruitment, osteoclastogenesis, and systemic inflammation [41,42]. MD confirmed stable binding, suggesting that kaempferol may inhibit IL‑1β signalling by locking the protein in a rigid conformation—a hypothesis requiring experimental validation [43].

Deoxycholic acid, a secondary bile acid, has been associated with inflammatory processes, while *MMP1* degrades interstitial collagens and is upregulated in both periodontitis and arthritis, contributing to tissue destruction [44,45]. This complex exhibited strong binding affinity and MD confirmed highly stable binding, suggesting that deoxycholic acid may act as a potent MMP1 inhibitor. However, bile acids also activate inflammatory signalling via TGR5 and FXR, complicating therapeutic interpretation [46].

*ITGAM* encodes the αM subunit of integrin Mac‑1 (CD11b/CD18), essential for leukocyte adhesion and migration, and implicated in both periodontitis and arthritis [47]. Despite a moderate docking score, we included this complex to ensure coverage of *ITGAM*, as kaempferol has known integrin‑modulating activity [40]. MD confirmed specific and stable binding. Given that Mac‑1 mediates neutrophil firm adhesion to activated endothelium—dependent on prior P‑selectin/PSGL‑1 rolling—kaempferol may disrupt this adhesion cascade.

In line with integrative in silico drug screening studies such as Thankachan et al. on PTPN3 in breast cancer—which combined clinicopathological correlation, ROC analysis, molecular docking, and MD simulations to identify high‑affinity inhibitors—our multi‑layered computational pipeline strengthens the credibility of these predictions [48]. Nevertheless, we must emphasise that docking and MD simulations have inherent limitations: docking methodologies are hypothesis generators, not precise predictors; binding energy alone is insufficient to infer biological relevance, and no consistent linear correlation exists between docking scores and experimental activity due to factors such as target flexibility, compound permeability, and scoring function limitations [49,50]. Furthermore, computational predictions cannot replace surface plasmon resonance for affinity measurement, cellular adhesion assays for functional assessment, or animal models for pharmacokinetic evaluation. Therefore, we make no therapeutic claims based on these results. The identified compounds represent prioritised candidates for future experimental investigation, rather than validated interventions.

In summary, this study identifies *SELP* as a robust cross-model predictor and *CXCL1* as a protective factor potentially linking PD and sJIA through shared inflammatory–vascular pathways. However, all findings are purely computational and derived from cross‑tissue data (gingiva vs. blood) without experimental validation. Therefore, we make no therapeutic claims; these results are exploratory and hypothesis‑generating, requiring future validation in matched tissue cohorts and functional studies.

The clinical utility of this signature – including its performance in prospective cohorts, its cost effectiveness, and its added value over existing clinical parameters – remains to be established. This study should be interpreted as a hypothesis generating discovery phase, not as a clinically ready diagnostic tool.

## 4. Materials and methods

### 4.1. Data sets

The periodontitis(PD) and systemic juvenile idiopathic arthritis(sJIA) gene expression data were obtained from the Gene Expression Omnibus(GEO) database(https://www.ncbi.nlm.nih.gov/geo/). For PD, the GSE16134 and GSE10334 datasets were derived from gingival tissue. GSE16134 served as the training set and contained 310 gingival tissue samples (241 diseased and 69 non‑diseased) from 120 patients (65 chronic, 55 aggressive periodontitis). Clinical details available for GSE16134 include: mean age 39.9 years (range13–76), 50.8% male, all non‑smokers, non‑diabetic, and no use of systemic antibiotics/anti‑inflammatories within 6 months; periodontitis was defined by proximal pocket depth(PD) >4 mm, clinical attachment loss(CAL) ≥3 mm, and BoP(+), while healthy controls had PD ≤ 4 mm, CAL ≤ 2 mm, and BoP(-). GSE10334 was used as the validation set and included gene expression data from 90 patients (63 chronic, 27 aggressive) with 183 diseased and 64 healthy tissue samples. For GSE10334, the same disease criteria (PD > 4 mm, CAL ≥ 3 mm, BoP(+) for cases; PD ≤ 4 mm, CAL ≤ 2 mm, BoP(-) for controls) and exclusion of smokers and systemic medication users were confirmed, but age and sex distributions were not publicly available.

For sJIA, three datasets derived from blood samples were used. GSE80060 (33 patients, 22 normal controls) and GSE7753 (17 patients, 30 normal controls) served as the training sets, counting 50 sJIA patients and 52 normal controls. GSE8650 (27 patients, 9 normal controls) was used as the validation set. To minimise batch effects across the training sets, we applied multi‑stage normalisation: first, Surrogate Variable Analysis (SVA) was used to model and adjust for potential confounding factors in the discovery cohort; then, ComBat was applied to further correct residual batch variations within a parametric empirical Bayes framework (with par.prior = TRUE). Corrected principal component analysis (PCA) confirmed successful data integration, as evidenced by clustering of batch samples in the reduced‑dimensionality space. Complete R code for the normalisation and diagnostic procedures is provided (S1 Code). A comprehensive summary of all dataset characteristics is provided (Table 1).

**Table 1:** A detailed summary of the GEO gene expression datasets.

GEO Accession	Platform	Case (Disease)	Control (Healthy)	Tissue Type	Organism
GSE16134	GPL570	241 PD	69	Gingival tissue	Homo sapiens
GSE10334	GPL570	183 PD	64	Gingival tissue	Homo sapiens
GSE80060	GPL570	33 sJIA	22	Blood tissue	Homo sapiens
GSE7753	GPL570	17 sJIA	30	Blood tissue	Homo sapiens
GSE8650	GPL96	27 sJIA	9	Blood tissue	Homo sapiens

The following information: accession number, platform, case versus control distribution, tissue type and organism.

### 4.2. Identification of DEGs

The R programme (version 4.4.3) is employed to standardise and process the original genetic expression matrix. The R package “limma” was utilized to identify differentially expressed genes(DEGs) from the GSE16134 and batch-merged sJIA dataset(GSE80060, GSE7753). The selection criterion employed was that of an adjusted *p-value* < 0.05 and an |log fold change (FC)| > 0.585. Utilising the R software, the differential gene clusters’ histograms and volcano plots were created.

### 4.3. WGCNA network construction and module identification

WGCNA is a popular algorithm employed to identify modules that are significantly associated with genes responsible for disease development [51]. The WGCNA R package was utilized to construct a co-expression network. The ‘hclust’ function was first applied to cluster samples to identify potential outliers. Subsequently, the ‘pickSoftThreshold’ function was used to determine the optimal soft-thresholding power β for scale-free topology (R² ≥ 0.85). The dynamic tree-cutting algorithm was then employed for module detection with a minimum module size of 50 and a module merging cut height of 0.25. Finally, gene significance (GS) and module membership (MM) were calculated to evaluate the relationship between genes and traits of interest. The analysis code for WGCNA is available (S2 Code).

### 4.4. Identification of shared genes and pathway enrichment

Overlapping genes between DEGs and WGCNA modules from both diseases were defined as preliminary candidates. These were further validated by consensus WGCNA, where Consensus TOM = min(TOM_PD, TOM_sJIA). Consensus connectivity was calculated and tested against random background via permutation tests. If the candidate genes we have identified maintain a high and stable degree of connectivity in the co-expression networks of both diseases, they are not differentially expressed genes shared by chance, but rather conserved network hub nodes common to both PD and sJIA. These candidate genes are designated as core genes and will be utilized in subsequent functional enrichment analyses. The use of R packages “enrichplot” and “ggplot2” is employed for the analysis of core genomic elements, including GO function, KEGG pathway enrichment analysis. The statistical analyses yield *p-value* < 0.05, and the results are rendered visually.

### 4.5. PPI network construction and hub genes selection

A comprehensive investigation was conducted using the STRING database (https://string-db.org/) to establish a core shared PPI network analysis, integrating PPI into the Cytoscape (version 3.8.2) software to enable visual representation. The MCC algorithm of the cytoHubba plugin was then employed to assess the genetic relatedness.

### 4.6. Computational screening and prioritisation of candidate genes using machine learning and assessing diagnostic accuracy

The objective of this computational step was not to provide biological discovery per se, but to perform unbiased, systematic feature reduction to generate a shortlist of high confidence candidates for subsequent biological interrogation.To systematically identify shared core diagnostic markers for PD and sJIA, we established a comprehensive machine learning prediction framework integrating multiple algorithms. Utilizing expression profile data from the training set, we employed fifteen classical machine learning algorithms(Lasso, SVM, RF, glmBoost, plsRglm, Stepglm, Ridge, Enet, GBM, LDA, QDA, XGBoost, AdaBoost, KNN and NaiveBayes) to develop 175 predictive models. The hyperparameters were optimized through five-fold cross-validation. The GSE16134 dataset of PD and the merged dataset(GSE80060, GSE7753) of sJIA were used as the training set, while GSE10334 and GSE8650 were used as the external validation set.Model performance was rigorously evaluated based on the area under the ROC curve (AUC), accuracy, and F1-score. The optimal single-model predictions were subsequently integrated using a stacking ensemble learning strategy. High-confidence models (AUC > 0.9) were selected, and their feature genes were ranked by frequency to identify candidate core genes. Finally, gene expression patterns were visualized using the pheatmap package. The machine learning code is provided (S3 Code).

In order to verify the expression levels and diagnostic efficacy of the core genes selected by the machine learning model in the training set and the validation set.The utilization of the “ggpubr” software facilitated the generation of box-and-violin plots, thereby enabling the visualization of the expression levels of core genes in the context of both PD and sJIA datasets. To assess the predictive and discriminatory capabilities of these core genes, receiver operating characteristic (ROC) curves were plotted, and the area under the curve (AUC) values were calculated using the “pROC” software.

### 4.7. Model Interpretation

In this study, we employed the SHAP (SHapley Additive exPlanations) algorithm to quantify each feature’s contribution to the model’s predictions. This approach enhances the interpretability of the internal decision-making process by assigning importance values to individual features, thereby facilitating a transparent assessment of their predictive impact. The SHAP interpretation code is provided (S4 Code).

### 4.8. Immune infiltration analysis and the association between biomarkers and immune cells

The CIBERSORT algorithm is a computational method used to calculate the percentage of different immune cell types [52]. We employed the method of immune cell infiltration analysis using the LM22 genetic set. In order to calculate the correlation between the core biological markers and the immune cells, we employed the spearman method. We also evaluated the percentage of cells in the test and control groups. We calculated the association between the genes selected by the computer program and the immune infiltration. We employed the Benjamini-Hochberg procedure for adjusting the p-value.

### 4.9. Identification of drug candidates

We obtained the file containing the association between compounds and genes from CTD Database (http://ctdbase.org/). This file revealed the essential hubs of the PPI networks for PD and sJIA, as well as the key genes associated with machine learning selection. TCMSP(https://www.tcmsp-e.com/) is a unique system pharmacology platform for traditional Chinese medicine that captures the relationships among drugs, targets, and diseases [53]. We utilized the TCMSP database to compile information regarding the target compounds (S1 Data), while concurrently employing the R package “clusterProfiler” to conduct a significance-based investigation of the molecular complexes. Our selection criterion entailed the adjusted *p-value* < 0.05 and the subsequent identification of the corresponding molecular complexes and significant targets (S2 Data). Concurrently, the relationship between the genes that are important targets of the compounds is imported into Cytoscape, thereby rendering the network of genes that are important targets of the compounds visible.

### 4.10. Molecular docking

The potential compounds are selected using a combination of network-scale gene control and relevant literature. This selection is then compared with the corresponding target genes to establish a molecular connection. The three-dimensional structure of the central protein was retrieved from the PDB database (http://www.rcsb.org/pdb/). The molecular structure of the pharmaceutical compound of interest was retrieved from the PubChem database (http://pubchem.ncbi.nlm.nih.gov/) and converted into the Mol2 format using the Open Babel software. The proteins were subjected to a process involving desiccation, hydroxylation and other preparatory steps. The input files for docking were prepared using AutoDock Tools (version 1.5.7), and the molecular docking calculations were subsequently performed with AutoDock Vina (version 1.2.7) [11]. The resulting complexes were saved as PDBQT files, which were then rendered using PyMOL (version 3.2) to visualise the most significant binding interactions [54].

### 4.11. Molecular dynamics simulation

Molecular dynamics (MD) simulations were performed using GROMACS 2022.3 for 100 ns, with the initial conformation derived from the optimal binding pose of the small‑molecule ligand with the target protein as determined by AutoDock Vina [55–57]. The AMBER14SB force field and the TIP3P water model were utilized for proteins, while the GAFF force field was employed for small molecules. Subsequently, the protein and small molecule ligands were merged to construct the simulation system of the complexes. The simulations were executed under constant temperature and pressure conditions, in conjunction with periodic boundary conditions. During MD simulations, all constraints involving hydrogen bonding were executed using the LINCS algorithm with an integration step of 2 fs. The cutoff value for non-bonding interactions was set to 10 Å and updated at 10-step intervals. The V-rescale temperature coupling method was employed to regulate the simulation temperature to 298 K, and the Berendsen method was utilized to control the pressure to 1 bar. To this end, NVT and NPT equilibrium simulations were carried out at 298 K for 100 ps, and MD simulations were performed for 100 ns for the complex system, with the conformation saved every 10 ps. Subsequent to the completion of the simulations, the simulation trajectories were subjected to analysis using VMD and PyMOL. Furthermore, the molecular mechanics Poisson-Boltzmann surface area (MMPBSA) binding free energy analysis between the protein and the small-molecule ligand was performed using the g_mmpbsa program. The resulting trajectories were analyzed, and the relevant data plots were generated using QtGrace(version 0.2.7**)** and custom Python scripts for visualization.

### 4.12. Limitations

The present study is not without its limitations. Firstly, there was an absence of prospective patient cohorts for the independent validation of our findings. Secondly, the study was hindered by the absence of same-tissue-type datasets, specifically PD blood or sJIA synovium, which were necessary to facilitate matched-tissue cross-disease comparisons. This limitation arose due to the utilization of data derived from gingiva (PD) and peripheral blood (sJIA) in the study. Thirdly, experimental validation in vitro or in vivo was lacking for the identified genes, immune infiltration patterns, and drug-target interactions, as all results are computational predictions. Fourthly, we lacked direct functional assays to confirm the binding and activity of the docked compounds. The fifth issue pertains to the methodology employed in determining the immune cell fractions. Rather than relying on experimental measurements, these fractions were inferred through the application of an algorithm. Ultimately, the sample size for specific analyses was constrained by the availability of public data.

## Supporting information

S1 Fig This figure presents a sensitivity analysis of WGCNA module counts under various gene-filtering thresholds for the PD (left) and sJIA (right) datasets. (TIF)

S2 Fig This figure is a scatter plot of module membership(MM) versus gene significance(GS) in WGCNA analysis, with the turquoise module strongly associated with PD and the pink, green, and brown modules strongly associated with sJIA. (TIF)

S3 Fig This figure is designed to assess the consensus connectivity of candidate genes relative to the background distribution, in order to test whether they act as conserved hub nodes in the co‑expression networks of both PD and sJIA. (TIF)

S1 Table This table presents the learned parameter values of the Ridge and AdaBoost models from the SHAP‑based interpretation analyses on the PD and sJIA datasets, respectively. (XLSX)

S1 Code R script for batch merging of the sJIA dataset. (R)

S2 Code R script for Weighted Gene Co‑expression Network Analysis(WGCNA). (R)

S3 Code R script for training and validation of 175 machine learning models. (R)

S4 Code R script for SHAP-based interpretation analysis. (R)

S1 Data This dataset contains the reference traditional Chinese medicine names and their corresponding active constituents. (XLSX)

S2 Data This dataset contains filtered information on traditional Chinese medicine–ingredient–target associations. (XLSX)
