# Supplementary material for: Shared pathogenic genes and therapeutic targets in periodontitis and systemic juvenile idiopathic arthritis: An integrative bioinformatics and machine learning study
Source: PLoS Comput Biol. 2026 Sep 23;22(9):e1014669. doi: 10.1371/journal.pcbi.1014669 (PMC13600523; doi:10.1371/journal.pcbi.1014669)
Supplement: Supplementary file 1 [file pcbi.1014669.s011.pdf]

PLOS Computational Biology

The integration of bioinformatics with machine learning and molecular docking has been demonstrated to identify shared pathogenic genes and therapeutic targets between periodontitis and systemic juvenile idiopathic arthritis

--Manuscript Draft--

**Manuscript Number:**	PCOMPBIOL-D-26-00673
**Full Title:**	The integration of bioinformatics with machine learning and molecular docking has been demonstrated to identify shared pathogenic genes and therapeutic targets between periodontitis and systemic juvenile idiopathic arthritis
**Short Title:**	shared genes between periodontitis and systemic juvenile idiopathic arthritis
**Article Type:**	Research Article
**Keywords:**	Periodontitis; Systemic juvenile idiopathic arthritis; Common genes; Shap; Chinese medicine,Macromolecular docking
**Abstract:**	Periodontitis (PD) is associated with systemic diseases, and systemic juvenile idiopathic arthritis (sJIA) is an autoimmune disease. The two diseases have a certain correlation, but their underlying mechanisms remain unclear. This study aimed to explore the shared mechanisms and potential therapeutic targets of PD and sJIA using genetic expression data from the GEO database, combined with differential expression gene (DEG) analysis, weighted gene co-expression network analysis (WGCNA), Gene Ontology (GO), Kyoto Encyclopedia of Genes and Genomes (KEGG), machine learning algorithms (LASSO, SVM-RFE), SHapley Additive exPlanations (SHAP), CIBERSORT immune infiltration analysis and molecular docking. The results identified 20 shared key genes. SELP, MS4A4A, MMP1, BCL2A1 and FAM46C were core diagnostic genes, among which SELP was the most important gene. SHAP analysis showed it had good explanatory power in two datasets, and it exhibited optimal diagnostic efficacy (AUC > 0.8) in both training and testing sets. Immune infiltration analysis revealed significant differences in neutrophils between PD and sJIA patients. IL1B, TNFAIP6, BCL2A1 and MMP1 were potential drug targets, with strong binding affinity between MMP1 and senkirkine, as well as BCL2A1 and eriodictyol, kaempferol, naringenin. This study reveals the shared genetic and immunological characteristics of PD and sJIA, highlighting SELP as the most critical diagnostic gene and providing new insights for their pathogenesis and potential therapeutic targets.Periodontitis (PD) is associated with systemic diseases, and systemic juvenile idiopathic arthritis (sJIA) is an autoimmune disease. The two diseases have a certain correlation, but their underlying mechanisms remain unclear. This study aimed to explore the shared mechanisms and potential therapeutic targets of PD and sJIA using genetic expression data from the GEO database, combined with differential expression gene (DEG) analysis, weighted gene co-expression network analysis (WGCNA), Gene Ontology (GO), Kyoto Encyclopedia of Genes and Genomes (KEGG), machine learning algorithms (LASSO, SVM-RFE), SHapley Additive exPlanations (SHAP), CIBERSORT immune infiltration analysis and molecular docking. The results identified 20 shared key genes. SELP, MS4A4A, MMP1, BCL2A1 and FAM46C were core diagnostic genes, among which SELP was the most important gene. SHAP analysis showed it had good explanatory power in two datasets, and it exhibited optimal diagnostic efficacy (AUC > 0.8) in both training and testing sets. Immune infiltration analysis revealed significant differences in neutrophils between PD and sJIA patients. IL1B, TNFAIP6, BCL2A1 and MMP1 were potential drug targets, with strong binding affinity between MMP1 and senkirkine, as well as BCL2A1 and eriodictyol, kaempferol, naringenin. This study reveals the shared genetic and immunological characteristics of PD and sJIA, highlighting SELP as the most critical diagnostic gene and providing new insights for their pathogenesis and potential therapeutic targets.
**Additional Information:**	
**Question**	**Response**
**Government Employee**	No - No authors are employees of the U.S. government.

Powered by Editorial Manager® and ProduXion Manager® from Aries Systems Corporation

Are you or any of the contributing authors an employee of the United States government?<br><br>Manuscripts authored by one or more US Government employees are not copyrighted, but are licensed under a CC0 Public Domain Dedication, which allows unlimited distribution and reuse of the article for any lawful purpose. This is a legal requirement for US Government employees.<br><br>This will be typeset if the manuscript is accepted for publication.	
**Financial Disclosure**<br><br>Enter a financial disclosure statement that describes the sources of funding for the work included in this submission. Review the submission guidelines for detailed requirements. View published research articles from *PLOS Computational Biology* for specific examples.<br><br>This statement is required for submission and **will appear in the published article** if the submission is accepted. Please make sure it is accurate.<br><br>**Funded studies**<br>Enter a statement with the following details:<br>• Initials of the authors who received each award<br>• Grant numbers awarded to each author<br>• The full name of each funder<br>• URL of each funder website<br>• Did the sponsors or funders play any role in the study design, data collection and analysis, decision to publish, or preparation of the manuscript?<br><br>Did you receive funding for this work?	The author(s) received no specific funding for this work.
**Competing Interests**	The author have declared that no competing interests exist.

On behalf of all authors, disclose any competing interests that could be perceived to bias this work.<br><br>This statement will be typeset if the manuscript is accepted for publication.<br><br>Review the instructions link below and PLOS Computational Biology's competing interests policy to determine what information must be disclosed at submission.	
**Data and Code Availability**<br><br>From the time of publication, Authors are required to make fully available and without restriction all data and computational code underlying their findings. Please see our PLOS Data Policy page for detailed policy information, and our Code Sharing page for specific information on code sharing.<br><br>A **Data Availability Statement**, detailing where the data (and code, if applicable) can be accessed, is required at first submission. Insert your Data Availability Statement in the box below. The statement you provide **will be published in the article**, if accepted.<br><br>Please see the Data Reporting section of our submission guidelines for instructions on what you need to include in your Data Availability Statement.<br><br>If the data and code are all contained in your submission files, please state: *All relevant data are within the manuscript and its Supporting Information files.*<br><br>**PLOS allows rare exemptions to address legal and ethical concerns.** If you have legal or ethical restrictions, please detail these in your Data Availability Statement below for the Journal team to consider.	All relevant data are within the manuscript and its Supporting Information files. The gene expression datasets analyzed in this study are publicly available from the Gene Expression Omnibus (GEO) database under accession numbers: [GSE16134, GSE10334,GSE80060,GSE7753,GSE8650].

Powered by Editorial Manager® and ProduXion Manager® from Aries Systems Corporation

Click here to access/download;Manuscript;manuscript plos.docx

The integration of bioinformatics with machine learning and molecular docking has been demonstrated to identify shared pathogenic genes and therapeutic targets between periodontitis and systemic juvenile idiopathic arthritis

Qingao Deng[1], Junjie Wang[2], Xing Wang[1]*, Lu Qi[1*]

[1] The First Affiliated Hospital of Xinjiang Medical University, Urumqi, Xinjiang Uygur Autonomous Region, China

[2]The Second Affiliated Hospital of Xinjiang Medical University, Urumqi, Xinjiang Uygur Autonomous Region, China

Corresponding Authors

*Xing Wang: Department of Prosthodontics, The First Affiliated Hospital of Xinjiang Medical University, No.137, South Liyu Road, New District, Urumqi, Xinjiang Uygur Autonomous Region, China, 830013

*Lu Qi: Department of Endodontics, The Second Affiliated Hospital of Xinjiang Medical University, No. 38, North Second Lane, Nanhu East Road, Urumqi, Xinjiang Uygur Autonomous Region, China, 830000

E-mail: xydefyql@xjmu.edu.cn(LQ)

Abstract

Periodontitis (PD) is associated with systemic diseases, and systemic juvenile idiopathic arthritis (sJIA) is an autoimmune disease. The two diseases have a certain correlation, but their underlying mechanisms remain unclear. This study aimed to explore the shared mechanisms and potential therapeutic targets of PD

and sJIA using genetic expression data from the GEO database, combined with differential expression gene (DEG) analysis, weighted gene co-expression network analysis (WGCNA), Gene Ontology (GO), Kyoto Encyclopedia of Genes and Genomes (KEGG), machine learning algorithms (LASSO, SVM-RFE), SHapley Additive exPlanations (SHAP), CIBERSORT immune infiltration analysis and molecular docking. The results identified 20 shared key genes. *SELP, MS4A4A, MMP1, BCL2A1* and *FAM46C* were core diagnostic genes, among which *SELP* was the most important gene. SHAP analysis showed it had good explanatory power in two datasets, and it exhibited optimal diagnostic efficacy (AUC $>$ 0.8) in both training and testing sets. Immune infiltration analysis revealed significant differences in neutrophils between PD and sJIA patients. *IL1B, TNFAIP6, BCL2A1* and *MMP1* were potential drug targets, with strong binding affinity between *MMP1* and senkirkine, as well as *BCL2A1* and eriodictyol, kaempferol, naringenin. This study reveals the shared genetic and immunological characteristics of PD and sJIA, highlighting *SELP* as the most critical diagnostic gene and providing new insights for their pathogenesis and potential therapeutic targets.

Keywords: Periodontitis, Systemic juvenile idiopathic arthritis, Common genes, Shap, Chinese medicine，Macromolecular docking

1. introduction

Periodontitis (PD) is a prevalent condition that manifests in the global population. Its prevalence is estimated to be 11% [1]. The clinical manifestations of PD primarily manifest as a deterioration of the gingiva, periodontal tissue, alveolar bone, and dentin due to bacterial infection [2]. The aetiology of the condition is the result of an interaction between oral bacteria and the host's immune system, leading to inflammation and immune responses mediated by the host's own immune response [3]. The extant evidence suggests a link between PD and a variety of systemic diseases, including autoimmune disorders [4].

Systemic Juvenile Idiopathic Arthritis (sJIA) is a rare form of juvenile idiopathic arthritis (JIA) characterised by fever, skin eruptions, and systemic inflammation, whose prevalence is estimated to range

between 10% and 20% of all cases of JIA[5].

Periodontitis, a prevalent chronic infectious inflammatory disease of the oral cavity, has garnered considerable attention in interdisciplinary research due to its association with autoimmune diseases. Compared to other types of JIA, sJIA's systemic inflammation storm makes it more unique in how it interacts with other types of JIA. Studies have shown that JIA patients are more likely to develop severe gum disease and periodontal disease [6]. In JIA cases, the temporomandibular joint (TMJ) is often affected, and there is a link between JIA and periodontal disease, based on the similarity of the two conditions' clinical inflammatory processes [7]. Th17 cells play a crucial role in many self-immune diseases, and their release contributes to PD, while cells associated with the Th1/Th17/Th22 axis are involved in temporomandibular joint osteoarthritis [8,9]. Additionally, the treatment for sJIA may affect oral health by regulating the immune system or altering the oral microenvironment. For example, glucocorticoids (such as prednisone) are commonly used to treat JIA, but long-term use can lead to gum overgrowth and disrupt the balance of oral bacteria, increasing the risk of periodontal disease. Research has shown that patients who use glucocorticoids for a long time are 20% to 30% more likely to develop gum disease [10].

However, there is insufficient evidence to date linking PD and sJIA. This study employed bioinformatics to analyze data from GEO database to identify shared PD and sJIA genetic factors. The objective was to identify the genes that cause these two diseases, their immune characteristics, and the mechanisms that underlie them. In addition, we sought to identify potential therapeutic targets for both diseases.

2. Materials and methods

2.1. Data Sets

The data on periodontitis and systemic juvenile idiopathic arthritis come from the GEO database(https://www.ncbi.nlm.nih.gov/geo/).The PD datasets for the gingivitis study GSE16134 and

GSE10334 come from gingival tissue, with GSE16134 being a training set with 310 samples of gingival tissue, 241 cases of diseased tissue, and 69 cases of non-diseased tissue, and GSE10334 being a validation set with 90 patients' gingival tissue gene expression data, 183 cases of diseased tissue, and 64 cases of healthy tissue. The GSE80060, GSE7753, and GSE8650 data sets of sJIA are derived from blood samples. GSE80060 and GSE7753 were used as training sets and contained 55 and 47 sJIA patients, respectively. GSE80060 contained 33 patients and 22 normal controls, and GSE7753 contained 17 patients and 30 normal controls. GSE8650 was used as a validation set and contained 36 patients, including 27 patients and 9 normal controls. The GSE80060 and GSE7753 data sets used multi-stage normalisation processing to reduce batch effects: Surrogate Variable Analysis (SVA) software was used to model and adjust for potential confounding factors in the discovery cohort. Combat coordinates the further correction of residual batch changes through the parameter-based Bayesian framework. Corrected Principal Component Analysis (PCA) showed that batch samples were clustered in the reduced-dimensionality space, confirming successful data coordination. The following is a complete breakdown of the work process (**Fig 1**) and a comprehensive summary of the characteristics of the datasets included in this study (**Table 1**).

Fig 1. Flowchart of the study.

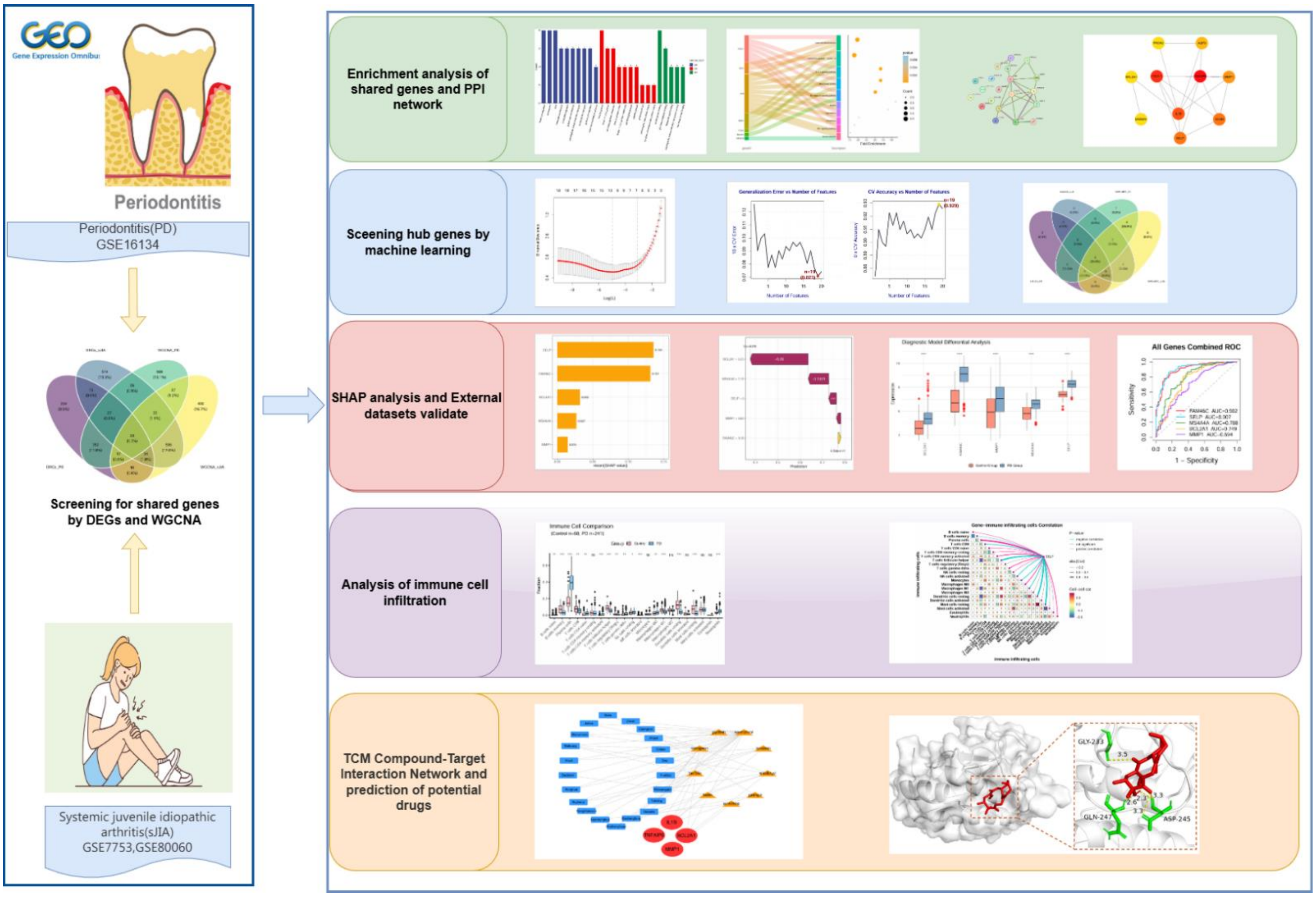

Table 1. A detailed summary of the GEO gene expression datasets.

GEO Accession	Platform	Case (Disease)	Control (Healthy)	Tissue Type	Organism
GSE16134	GPL570	241 PD	69	Gingival tissue	Homo sapiens
GSE10334	GPL570	183 PD	64	Gingival tissue	Homo sapiens
GSE80060	GPL570	33 sJIA	22	Blood tissue	Homo sapiens
GSE7753	GPL570	17 sJIA	30	Blood tissue	Homo sapiens
GSE8650	GPL96	27 sJIA	9	Blood tissue	Homo sapiens

The following information: accession number, platform, case versus control distribution, tissue type and organism.

2.2. Identification of DEGs

The R programme (4.4.2) is employed to standardise and process the original genetic expression matrix. The R package "limma" (version 3.62.1) was utilized to identify differentially expressed genes (DEGs) from the GSE16134 and Batch-merged sJIA dataset. The selection criterion employed was that of an adjusted *p-value* < 0.05 and an |log fold change (FC)| > 0.585. Utilising the R software, the differential gene clusters' histograms and volcano plots were created.

2.3. WGCNA network construction and module identification

WGCNA is a popular algorithm that is employed to identify modules that are significantly associated with the genes that are responsible for the development of the disease[11]. The WGCNA R software was employed to construct a network that facilitated data exchange. The initial step involved the incorporation of the top 25% of genes from the sample into the network, utilising the 'Hclust' function to perform clustering analysis. This process was undertaken to identify any potential anomalous values. Subsequently, the R software's pickSoftThreshold function was utilised to determine the soft-thresholding parameter β, which was then employed to conduct clustering analysis. The minimum module size employed was 50, and the maximum value was set at 0.25. Finally, the genetic significance and the relationship between the module components were calculated.

2.4. Identification of shared genes and pathway enrichment

A joint analysis was conducted of DEGs and WGCNA-selected genes, with the results then visualized in a Venn diagram. Overlapping genes are designated as core genes and will be utilized in subsequent functional enrichment analyses. The use of R packages "enrichplot" and "ggplot2" is employed for the analysis of core genomic elements, including GO function, KEGG pathway enrichment analysis. The statistical analyses yield

p-value < 0.05, and the results are rendered visually.

2.5. PPI network construction and hub genes selection

A comprehensive investigation was conducted using the STRING database (https://string-db.org/) to establish a core shared PPI network analysis, integrating PPI into the Cytoscape (3.8.2) software to enable visual representation. The MCC algorithm of the cytoHubba plugin was then employed to assess the genetic relatedness.

2.6. Core Feature selection by machine learning algorithm

Lasso is a regression analysis algorithm that can select variables and regularization, thereby minimizing overfitting to enhance the prediction's accuracy [12,13]. Support Vector Machines (SVM) represent a supervised machine learning method for classification and regression tasks. The Recursive Feature Elimination (RFE) algorithm is employed to optimise feature selection and enhance model performance [14]. Two machine learning algorithms, LASSO regression and SVM-RFE, were employed to identify core genes associated with PD and sJIA.The definition of 'core genes' was derived from the identification of those genes that were found to overlap across both of the aforementioned algorithms. The LASSO model was implemented using the 'glmnet' package (version 4.1-9), with the following parameter settings: 'standardize = True', 'alpha = 1', 'family = "binomial"', and 'nfolds = 10'. The SVM-RFE model was executed using the 'e1071' package (version 1.7-16).

2.7. Model Interpretation

in this study, we employed the SHAP (SHapley Additive exPlanations) algorithm to quantify the impact of each feature on the prediction outcome. This method effectively elucidates the internal decision-making process of a machine learning model. The SHAP algorithm assigns a value to each feature, enabling the assessment of the model's prediction impact with greater explicability.

2.8. Differential expression of core genes and assessing diagnostic accuracy

The GEO database was downloaded, including the PD training set GSE16334 and the validation set GSE10334, which contain the transcriptomic data of normal tissue and patients with gingival disease. The training set GSE80060 and validation set GSE7753 for sJIA, as well as the validation set GSE8650, which contains patients and controls for sJIA, were also downloaded. These were utilized for the analysis and visual representation of core genes. The utilization of the "ggpubr" software facilitated the generation of box-and-violin plots, thereby enabling the visualization of the expression levels of core genes in the context of both PD and sJIA datasets. To assess the predictive and discriminatory capabilities of these core genes, receiver operating characteristic (ROC) curves were plotted, and the area under the curve (AUC) values were calculated using the "pROC" software.

2.9. Immune infiltration analysis and the association between biomarkers and immune cells

The CIBERSORT algorithm is a computational method used to calculate the percentage of different immune cell types [15]. We employed the method of immune cell infiltration analysis using the LM22 genetic set. In order to calculate the correlation between the core biological markers and the immune cells, we employed the spearman method. We also evaluated the percentage of cells in the test and control groups. We calculated the association between the genes selected by the computer program and the immune infiltration. We employed the Benjamini-Hochberg procedure for adjusting the p-value.

2.10. Identification of drug candidates

We obtained the file containing the association between compounds and genes from CTD Database (http://ctdbase.org/). This file revealed the essential hubs of the PPI networks for PD and sJIA, as well as the key genes associated with machine learning selection. TCMSP(www.tcmsp-e.com) is a unique system

pharmacology platform for traditional Chinese medicine that captures the relationships among drugs, targets, and diseases[16]. We utilized the TCMSP database to compile information regarding the target compounds(**S1 file**), while concurrently employing the R package "clusterProfiler" to conduct a significance-based investigation of the molecular complexes. Our selection criterion entailed the adjusted *p-value* < 0.05 and the subsequent identification of the corresponding molecular complexes and significant targets(**S2 file**). Concurrently, the relationship between the genes that are important targets of the compounds is imported into Cytoscape, thereby rendering the network of genes that are important targets of the compounds visible.

2.11. Molecular docking

The potential compounds are selected using a combination of network-scale gene control and relevant literature. This selection is then compared with the corresponding target genes to establish a molecular connection. The three-dimensional structure of the central protein was retrieved from a database called PDB (http://www.rcsb.org/pdb/). The molecular structure of the pharmaceutical compound of interest was retrieved from the PubChem database (http://pubchem.ncbi.nlm.nih.gov/) and converted into the Mol2 format using the Open Babel software. The proteins were subjected to a process involving desiccation, hydroxylation and other preparatory steps. The molecular docking process was then initiated using AutoDock Tools (version 1.5.7). The resultant complexes were saved as PDBQT files, which were subsequently rendered using PyMOL (version 3.2) to visualise the most significant binding interactions [17].

3. Result

3.1. Identification of PD-Related and sJIA-Related Target Genes

In order to mitigate batch effects, we have consolidated the data sets GSE80060 and GSE7753, and we have also standardized the gene expression matrix resulting from the consolidation. PCA analysis of the constituent elements reveals that data with homogeneity demonstrate a greater degree of aggregation **(Fig 2**

a,b). As indicated by the analysis of differences in gene expression, 734 DEGs that are associated with PD were identified, with 491 up-regulated genes and 243 down-regulated genes demonstrated by the volcano**(Fig 2 c).** The heatmap highlights 25 of the most significant DEGs, demonstrating increased as well as decreased**(Fig 2 e)**. In addition, the comprehensive screening for sJIA identified 1231 DEGs. The heatmap demonstrates the presence of 685 up-regulated genes and 546 up-regulated genes**(Fig 2 d)**. Moreover, the heatmap highlights 25 of the most significant DEGs, demonstrating increased as well as decreased **(Fig 2 f)**.

Fig 2. Identification of differentially expressed genes(DEGs).

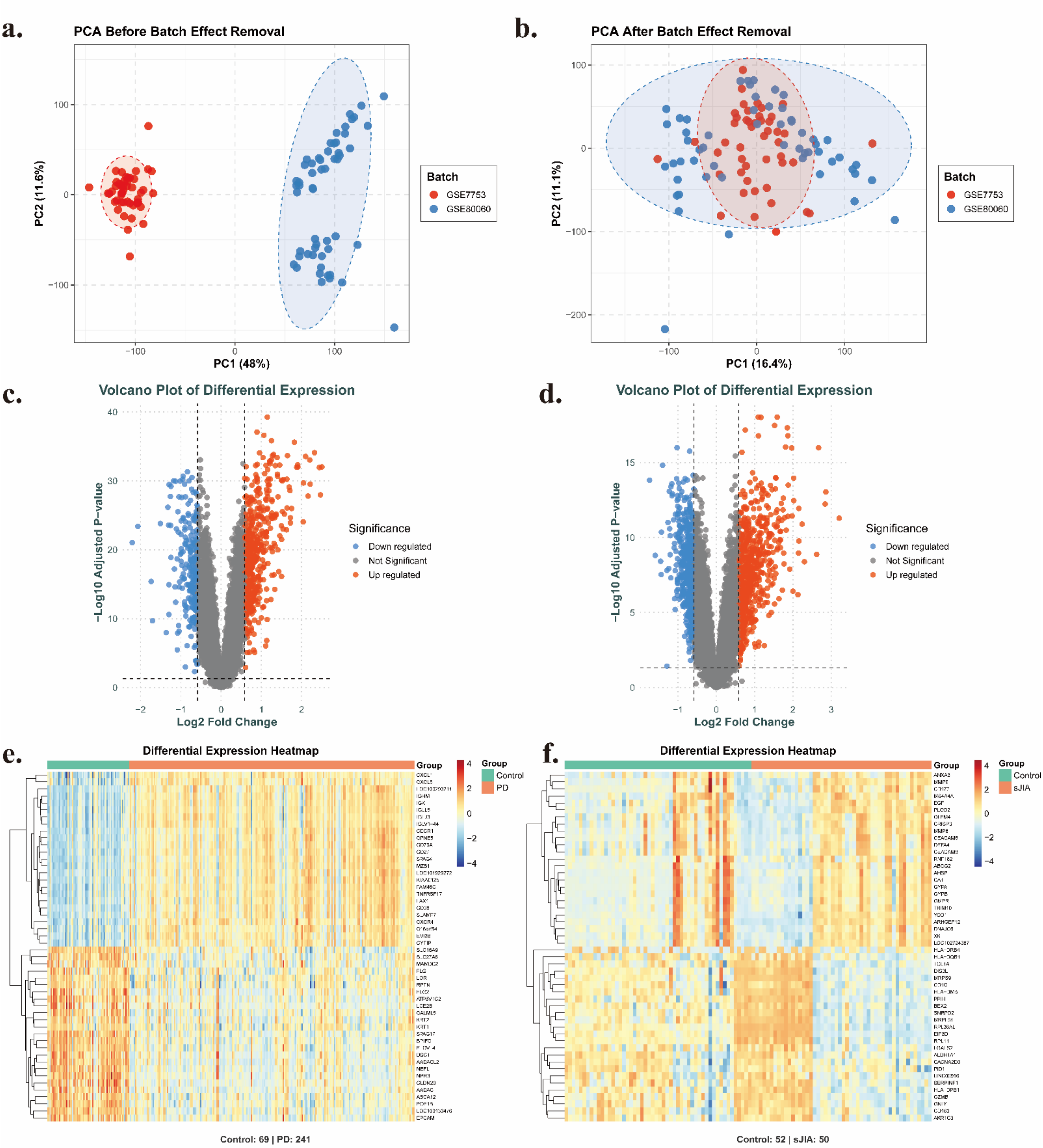

(a,b) PCA scatter plot shows distinct separation between GSE7753 and GSE80060 datasets before batch correction and after batch correction, indicating reduced batch effects.**(c,d)**Volcano plots depicting DEGs for datasets PD(GSE16134) and sJIA (GSE7753, GSE80060), respectively. Significantly upregulated genes were shown in red, significantly downregulated genes in blue, and non-significant genes in grey. **(e,f)** Heatmaps illustrating the expression patterns of representative significantly 25 upregulated and downregulated DEGs identified in PD (GSE16134) and sJIA (GSE7753,GSE80060) datasets.

3.2. Weighted gene coexpression network analysis of PD and sJIA

The WGCNA analysis was conducted on the integrated dataset consisting of PD and sJIA dataset. Initially, the optimal soft threshold value was determined to be 9 for PD and 13 for sJIA **(Fig3 a,b)**. Subsequently, modules with high similarity were merged, resulting in the identification of 11 modules in the PD dataset and 10 modules in the sJIA dataset **(Fig 3 c,d)**. Subsequently, the correlation between the modules and the traits must be determined. Within the PD framework, the turquoise modules demonstrate the strongest correlation with the traits ($r = 0.64$) **(Fig 3 e)**. In the case of sJIA, the modules that are red, yellowgreen, green, and black have a relatively higher degree of relatedness to sJIA than the other modules ($r > 0.6$) **(Fig 3 f)**. The modules' internal genes (GS) and modules' members (MM) have a strong relationship(the correlation coefficient of $r=0.987$ for the turquoise module of PD, $r=0.967$ for the red module, $r=0.815$ for the yellowgreen module and $r=0.841$ for the black module of sJIA).

Figure3. Weighted gene coexpression network analysis of PD and sJIA.

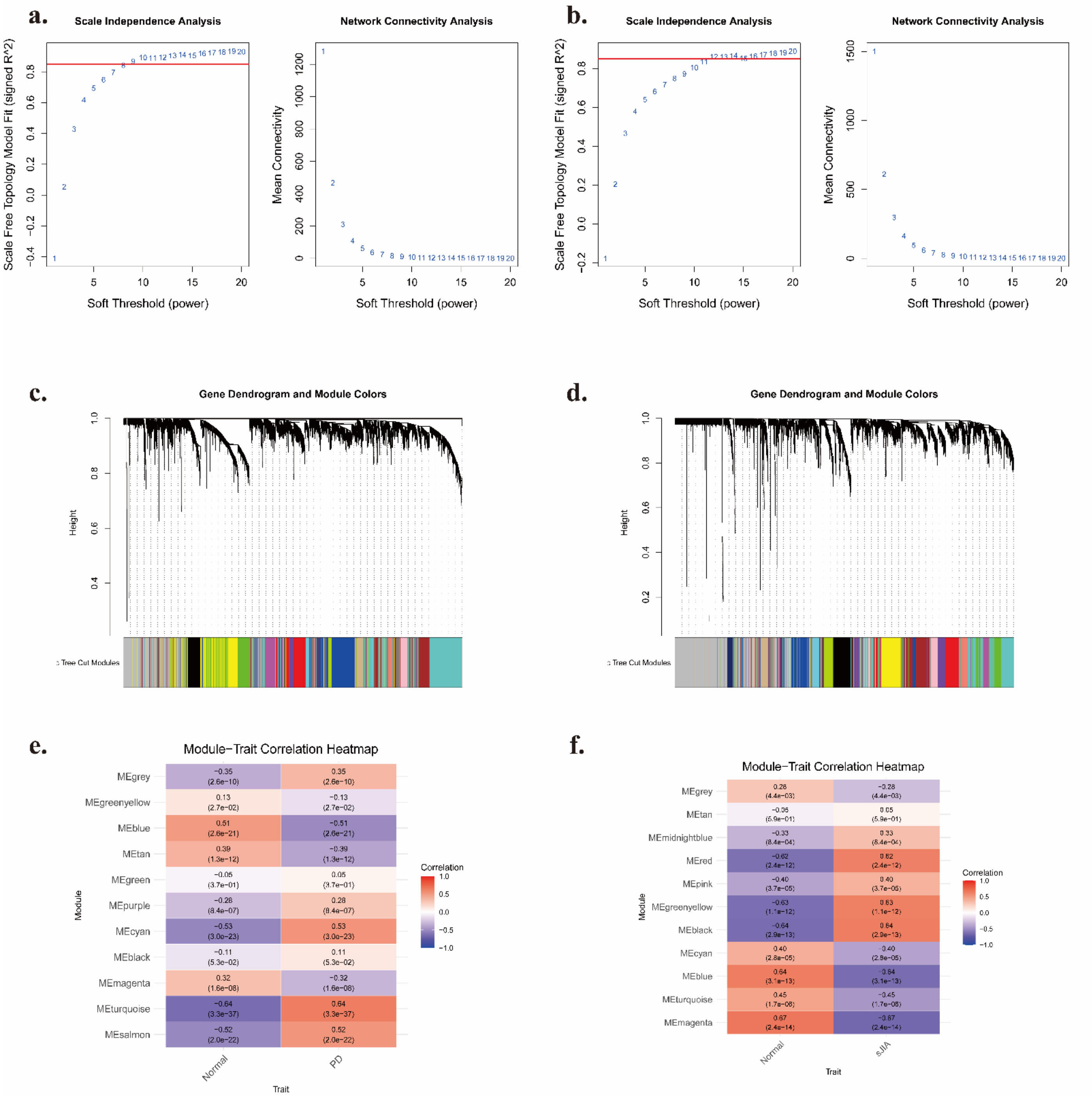

(a, b) Analysis of the scale-free fit index and mean connectivity to determine the best soft-thresholding power in PD and sJIA.**(c, d)** Clustering dendrograms of genes in PD and sJIA.**(e, f)** Heatmap of the correlation analysis of module eigengenes with clinical phenotypes in PD and sJIA. Red color represents positive correlation and blue color represents negative correlation.

3.3. Enrichment analysis of PD and sJIA shared genes

An analysis of the intersection of the DEGs from PD and sJIA, as well as those with a strong correlation, revealed 27 overlapping genes(**Fig 4 a)**. These genes are likely to play a pivotal role in the molecular

mechanisms of PD and sJIA.Consequently, we initially conducted Gene Ontology(GO) and Kyoto Encyclopedia of Genes and Genomes (KEGG) enrichment analysis on these genes. The outcomes of the study indicate that a significant proportion of the genes are concentrated in pathways related to lipid and atherosclerosis, NF-κB signaling, Coronavirus disease (Covid-19), IL−17 signaling pathway and Rheumatoid arthritis signaling transduction**(Fig 4 b,c)**. Subsequently, in order to identify additional genes with similar functions, 27 overlapping genes were entered into the String database**(Fig 4 d)**. Following the removal of independent genes, the CytoHubba MCC algorithm (Cytoscape software) was employed to identify the top 10 hub genes**(Fig 4 e)**.

Fig4. Functional enrichment and pathway enrichment of 20 shared genes.

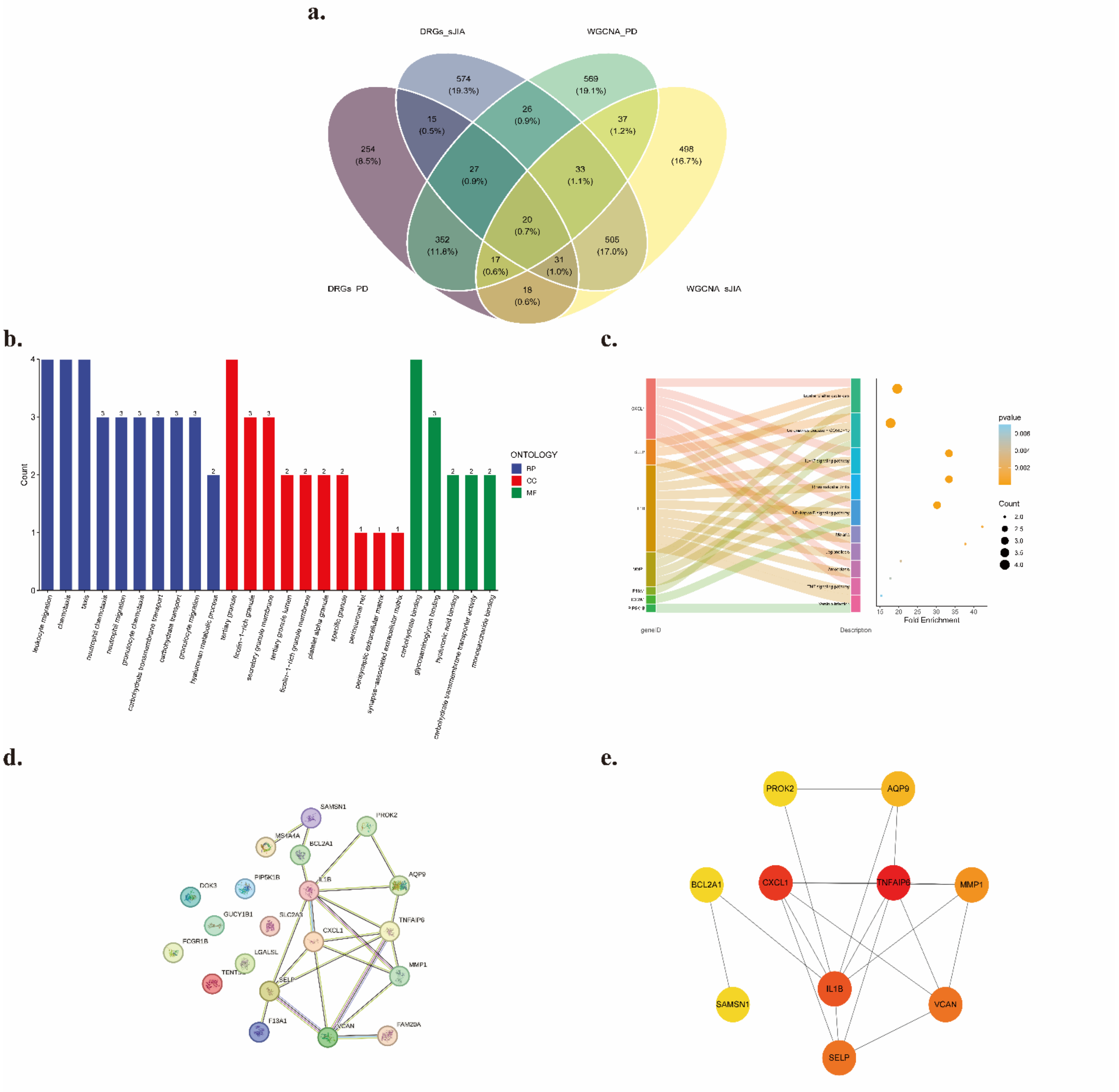

(a) Venn diagram of overlapping DEGs and gene module intersection between PD and sJIA.**(b)** Graph showing GO analysis of shared genes.**(c)** sankey+dot plot showing KEGG pathway analysis of shared genes.**(d)** PPI network of overlapping genes.**(e)** CytoHubba extracted the top 10 hub genes, ranked by MCC score, and these genes are highlighted in red with the MCC values indicated.

3.4. Dentification and validation of potential shared hub genes by and LASSO and SVM-RFE

The LASSO and SVM-REF machine learning algorithms were utilised in order to identify the genes with diagnostic value that were present in the 20 overlapping genes. The LASSO regression method was used to identify 13 genes from the PD dataset and 7 genes from the sJIA dataset**(Fig 5 a, b)**.. A total of 19 genes were selected from PD dataset using the SVM-REF method **(Fig 5 c)**. The sJIA dataset contained 14 genes **(Fig 5 d)**. The genes identified using these different methods were subsequently overlapped, resulting in the selection of five diagnostic biomarkers(*FAM46C, SELP, MS4A4A, BCL2A1,MMP1*) **(Fig 5 e)**.

Figure5. Identifying Potential Shared Diagnostic Genes Using LASSO Regression Models and SVM-REF Models.

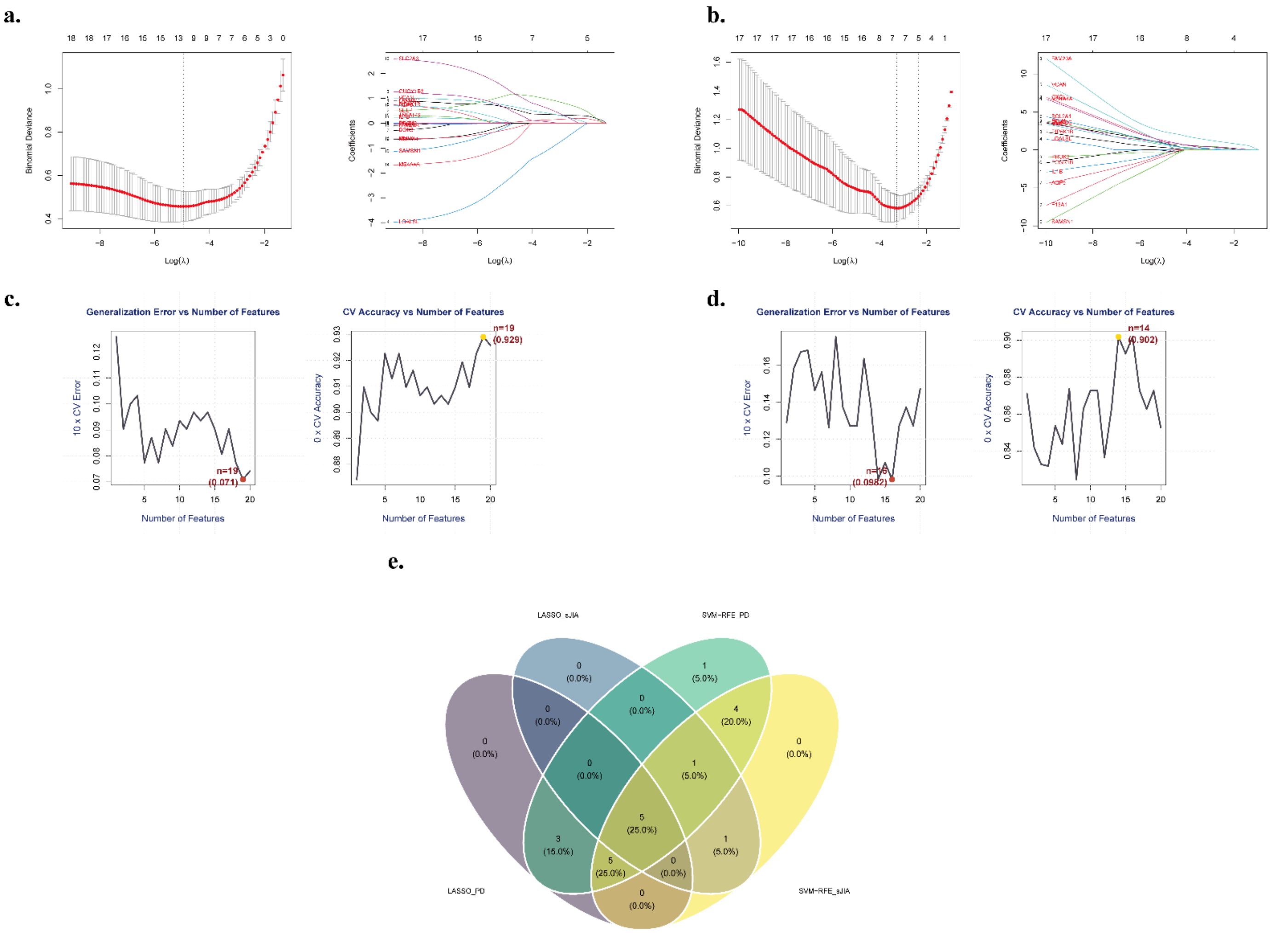

(a) Tenfold cross-validation to select the optimal tuning parameter log (lambda) and LASSO coefficient profiles of diagnostic genes in the PD dataset. **(b)**Tenfold cross-validation to select the optimal tuning

parameter log (lambda) and LASSO coefficient profiles of diagnostic genes in the sJIA dataset.**(c)** Effect of the number of features on the generalization performance of SVM-REF in the PD dataset.**(d)** Effect of the number of features on the generalization performance of SVM-REF in the sJIA dataset.**(e)** Venn diagram showing the optimal diagnostic biomarkers.

3.5. SHAP analysis and Diagnostic Model Validation

The following five machine learning models were utilized: RF, SVM, XGB, GBM, and KNN. The ROC curve demonstrates that, among the PD dataset, the RF model demonstrated the highest Area Under Curve (0.840)**(Fig 6 a)**. Among the sJIA dataset, the RF model demonstrated the highest Area Under Curve (0.969) **(Fig6 b)**. These two models were identified as the optimal selection for subsequent SHAP analysis. SHAP analysis revealed two five diagnostic genes in the global landscape **(Fig 6 c, d)**. In the PD dataset, SELP (SHAP value 0.135) and FAM46C (SHAP value 0.131) are considered the most influential risk factors, and BCL2A1 and MMP1 show dual regulatory effects**(Fig 6 e)**.while in the sJIA dataset, MS4A4A (SHAP value 0.178), SELP (SHAP value 0.090), BCL2A1(SHAP value 0.090) and MMP1(SHAP value 0.083) are the most influential factors. All genes exhibit positive regulation**(Fig 6 f)**.In the linear regression analysis, BCL2A1(6.55, Δ=-0.26) and MS4A4A (7.11, Δ= -0.00921) act as primary negative regulators, driving prediction values (f(x) = 0.376) below the benchmark expectation (E[f(x)] = 0.77) in PD dataset **(Fig 6 e)**. However, MS4A4A (6.17, Δ= +0.153), SELP (7.63, Δ= +0.139) and MMP1(3.64, Δ= +0.0.133) act as positive regulators, driving prediction values (f(x) = 0.93) exceed the benchmark expectation (E[f(x)] = 0.487) in sJIA dataset **(Fig 6 f)**.

371 **Fig6. Shap explanation analysis core diagnostic genes.**

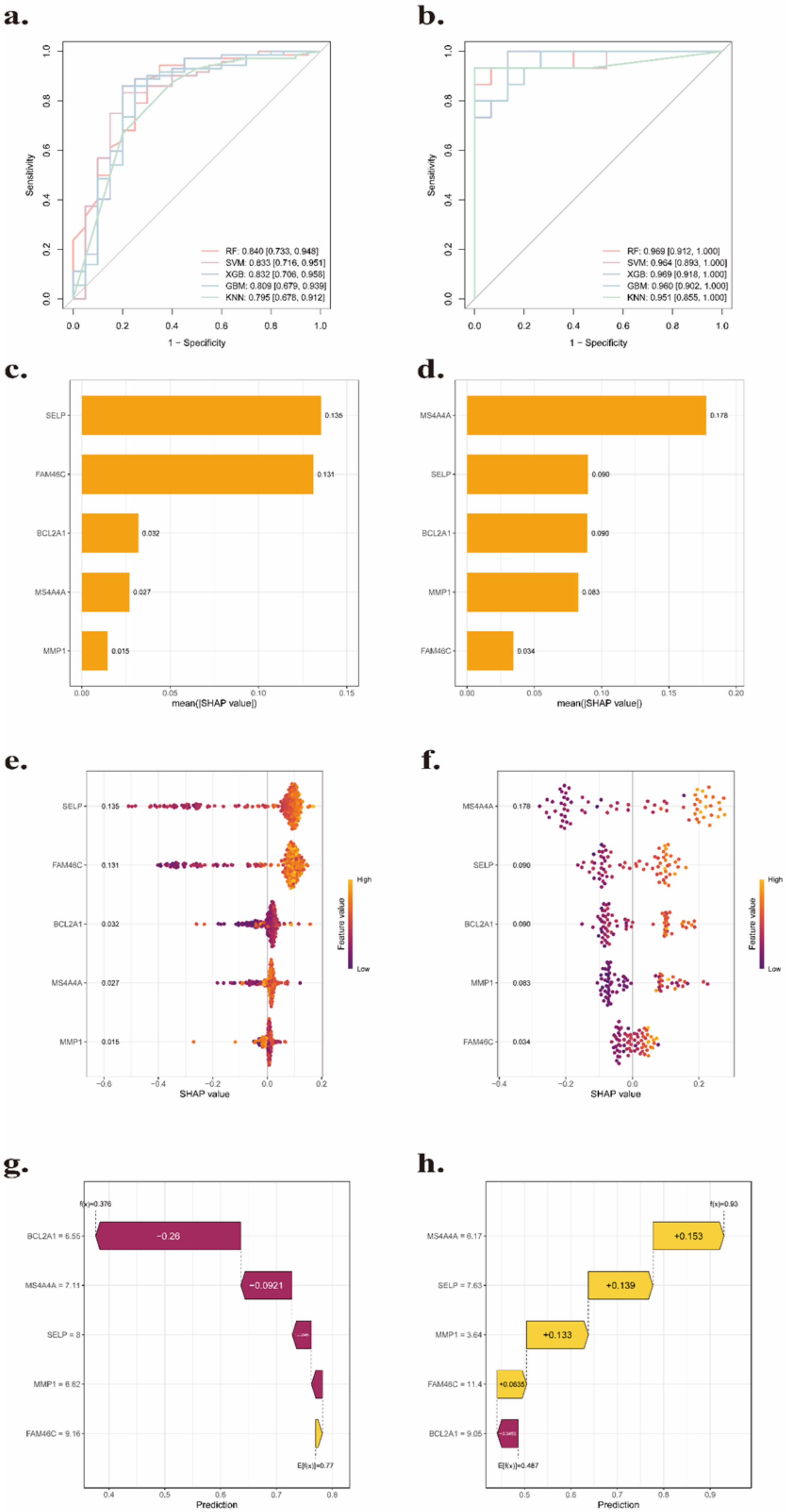

372

(a) Predicted area under the curve (AUC) values for each model in the PD dataset.**(b)** Predicted area under the curve (AUC) values for each model in the sJIA dataset.**(c)**Feature Importance Ranking in the PD dataset: A bar graph ranks top genes by feature importance(Larger bars = greater contribution to the mode).**(d)** Feature Importance Ranking in the sJIA dataset: A bar graph ranks top genes by feature importance.**(e)** Violin Plot in the PD dataset: violin plot shows gene expression distributions across conditions(Width = data density, colors = expression levels).**(f)** Violin Plot in the sJIA dataset: violin plot shows gene expression distributions across conditions.**(g)** SHAP Summary Plot in the PD dataset: SHAP summary plot shows gene contributions to predictions(Negative SHAP = lowering effect, positive = increasing effect).**(h)** SHAP Summary Plot in the sJIA dataset: SHAP summary plot shows gene contributions to predictions.

Subsequently, the results of the box-plot analysis were presented, indicating a notable increase in the five core genes of PD and sJIA training datasets in the disease group**(Fig 7 a, b)**. This finding was corroborated in the PD and SJIA validation datasets, wherein MMP1 gene expression exhibited no substantial variation in the sJIA test data sets between the disease and control groups**(Fig7 e, f)**. Concurrently, ROC curves are utilized to assess the diagnostic value of the core prediction genes across diverse datasets. In the context of the PD training dataset, it was observed that the area under the curve (AUC) values of SELP (AUC=0.907), FAM46C (AUC=0.902), MS4A4A(AUC=0.788), BCLA1(AUC=0.749) and MMP1(AUC=0.0.694) all exceeded 0.6 **(Fig 7 c)**.Similarly,*SELP*(AUC=0.910), *FAM46C*(AUC=0.851), *MS4A4A*(AUC=0.929), *BCLA1*(AUC=0.870) and *MMP1*(AUC=0.823) all exceeded 0.6**(Fig 7 d)**. In the PD validation dataset, t The AUC values for all five core genes exceed 0.6**(Fig 7 g)**. In the sJIA validation set, only *MMP1* (AUC=0.522) below 0.6, which is consistent with its differentially expressed results in the test set**(Fig 7 h)**.

Fig7. Expression pattern validation and diagnostic value.

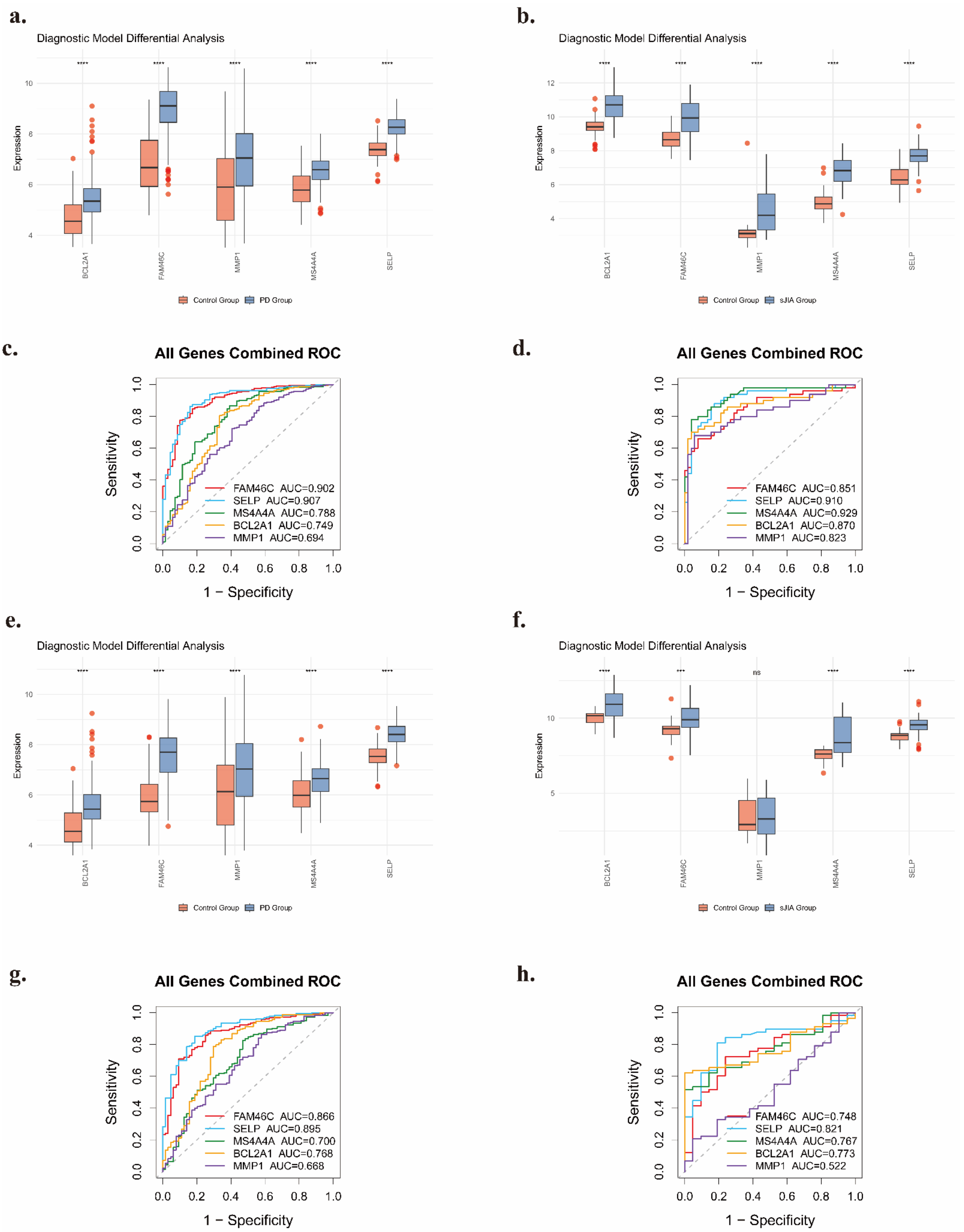

(a) Expression of *FAM46C, SELP, MS4A4A, BCL2A1* and *MMP1* in the PD training dataset. **(b)** Expression

of *FAM46C, SELP, MS4A4A, BCL2A1* and *MMP1* in the sJIA training dataset.**(c)** ROC curve of the shared diagnostic genes in the PD training dataset. **(c)** ROC curve of the shared diagnostic genes in the sJIA training dataset.**(e)** Expression of *FAM46C, SELP, MS4A4A, BCL2A1* and *MMP1* in the PD testing dataset.**(f)** Expression of *FAM46C, SELP, MS4A4A, BCL2A1* and *MMP1* in the sJIA testing dataset.**(g)** ROC curve of the shared diagnostic genes in the PD testing dataset.**(h)** ROC curve of the shared diagnostic genes in the sJIA testing dataset.

As demonstrated in the preceding analysis, *SELP* genes exhibit notable predictive value in the context of SHAP explanations. Specifically, the diagnostic efficacy of *SELP* genes is evident in both training and testing sets, surpassing that of other genes.Consequently, *SELP* genes have the potential to serve as a crucial diagnostic marker for PD and sJIA.

3.6. Immune cell infiltration and its correlation with shared hub genes

The present study employed the CIBERSORT method, which is based on 22 distinct immune cell types, to analyse the immune infiltration datasets of PD and sJIA. In the analysis of differences in expression, the PD and sJIA groups exhibited a consistent increase in neutrophil expression, while the T cell (CD8+) expression exhibited a consistent significant decrease**(Fig 8 a, b)**. These findings suggest that immune dysregulation and inflammatory responses may occur concurrently in PD and sJIA.

In addition to confirming the uniform distribution of immune cells, we also verified the association of the SELP diagnostic genetic element with the immune penetration of the lesion. Correlation analysis indicates **(Fig 8 c, d)** that SELP exhibits positive correlations with Neutrophil and Macrophages M0 in the PD dataset, while showing negative correlations with Mast cells resting and T cells CD8. A similar pattern of results was observed for sJIA data sets as well, suggesting the possibility of core genetic regulation through immune cell expression modulation.

Fig8. Analysis of immune infiltration.

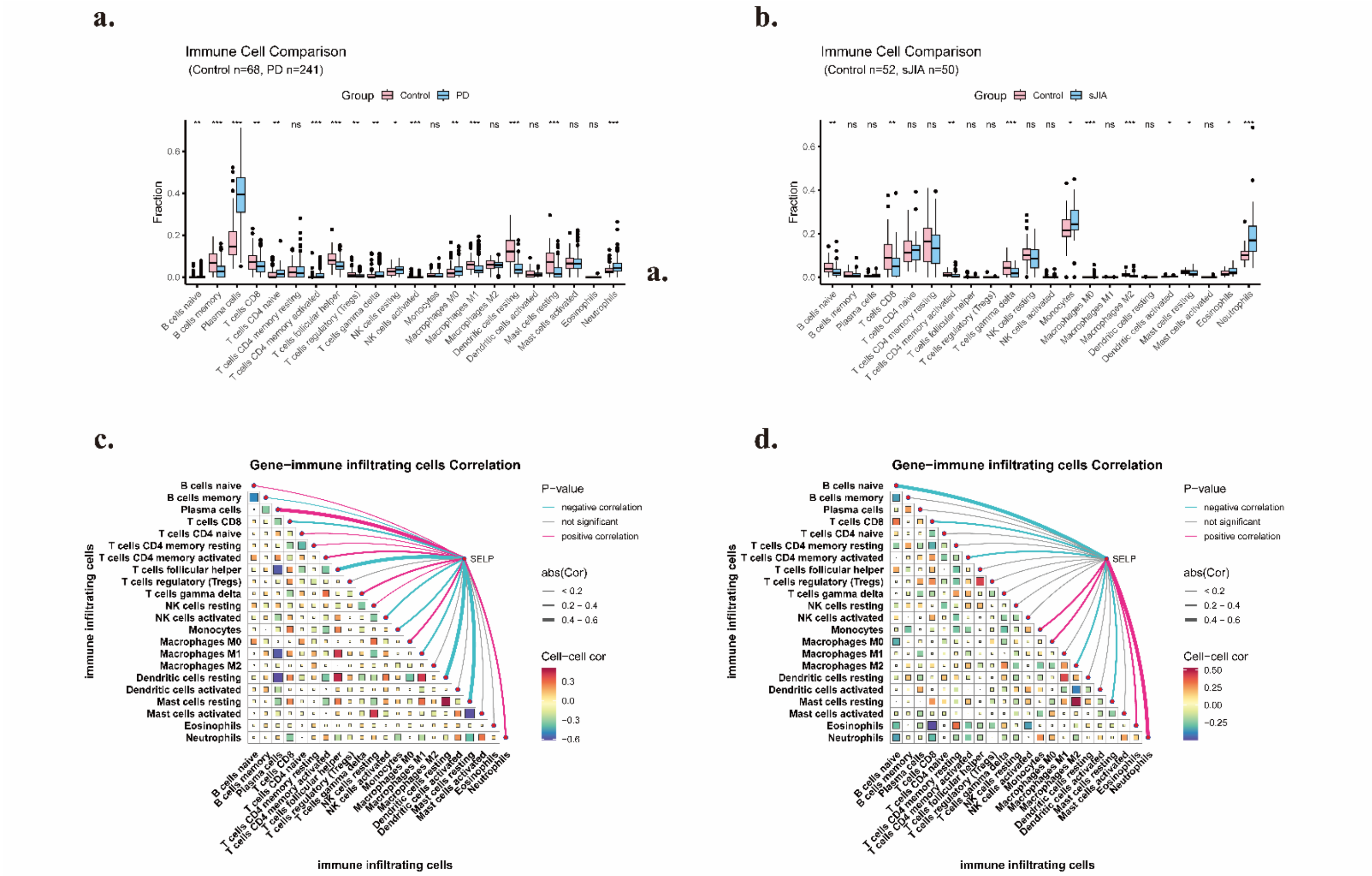

(a) showing the distribution of 22 immune cells in the PD training sample. **(b)** showing the distribution of 22 immune cells in the sJIA training sample. **(c)** Correlation between the *SELP* gene and immune cell infiltration in the PD training sample. **(d)** Correlation between the *SELP* gene and immune cell infiltration in the sJIA training sample.

3.7. Identification of candidate drugs based on hub genes and molecular docking analysis

We have developed a network of crucial target genes for traditional Chinese medicine (TCM) compounds by integrating the CTD and TCMSP databases(**Fig 9 a).** This network highlights the central role of the diagnostic gene MMP1 and the BCL2A1 gene in the regulation of this network.The target site of the compound senkirkine is the MMP1 gene, and the corresponding Chinese medicinal agent is Kuandonghua. The target site of the compounds eriodictyol, kaempferol and naringenin is the BCL2A1 gene, and the corresponding Chinese medicinal agents are Mahuanggen, Mahuang and Nvzhenzi, among others.

The determination of the core diagnostic gene MMP1 and its associated compounds, senkirkine, BCL2A1 and eriodictyol, kaempferol, and naringenin, was achieved through the use of molecular modeling. The molecular interaction between *MMP1*-specific substrate 3HSI and senkirkine has been calculated to be -6.8kcal/mol, whilst the binding affinity of *BCL2A1*-specific substrate 5WHI with eriodictyol, kaempferol, and naringenin has been determined to be -7.3, -6.7, and -7.1kcal/mol, respectively**(Fig 9 b, c, d, e)**. These values indicate the presence of significant binding affinity in all cases, suggesting a high level of specificity and stability.Compounds with binding constants below -5.0 kcal/mol are considered to have excellent binding affinity, while those with binding constants below -7.0 kcal/mol are considered to have strong binding affinity[18].It has been established that the binding affinity of eriodictyol and naringenin for *BCL2A1* is less than -7.0. This indicates that eriodictyol and naringenin exhibit stronger binding affinity for *BCL2A1* than for other target proteins.

Fig 9. Hub Gene-Based Reverse Prediction of Traditional Chinese Medicines.

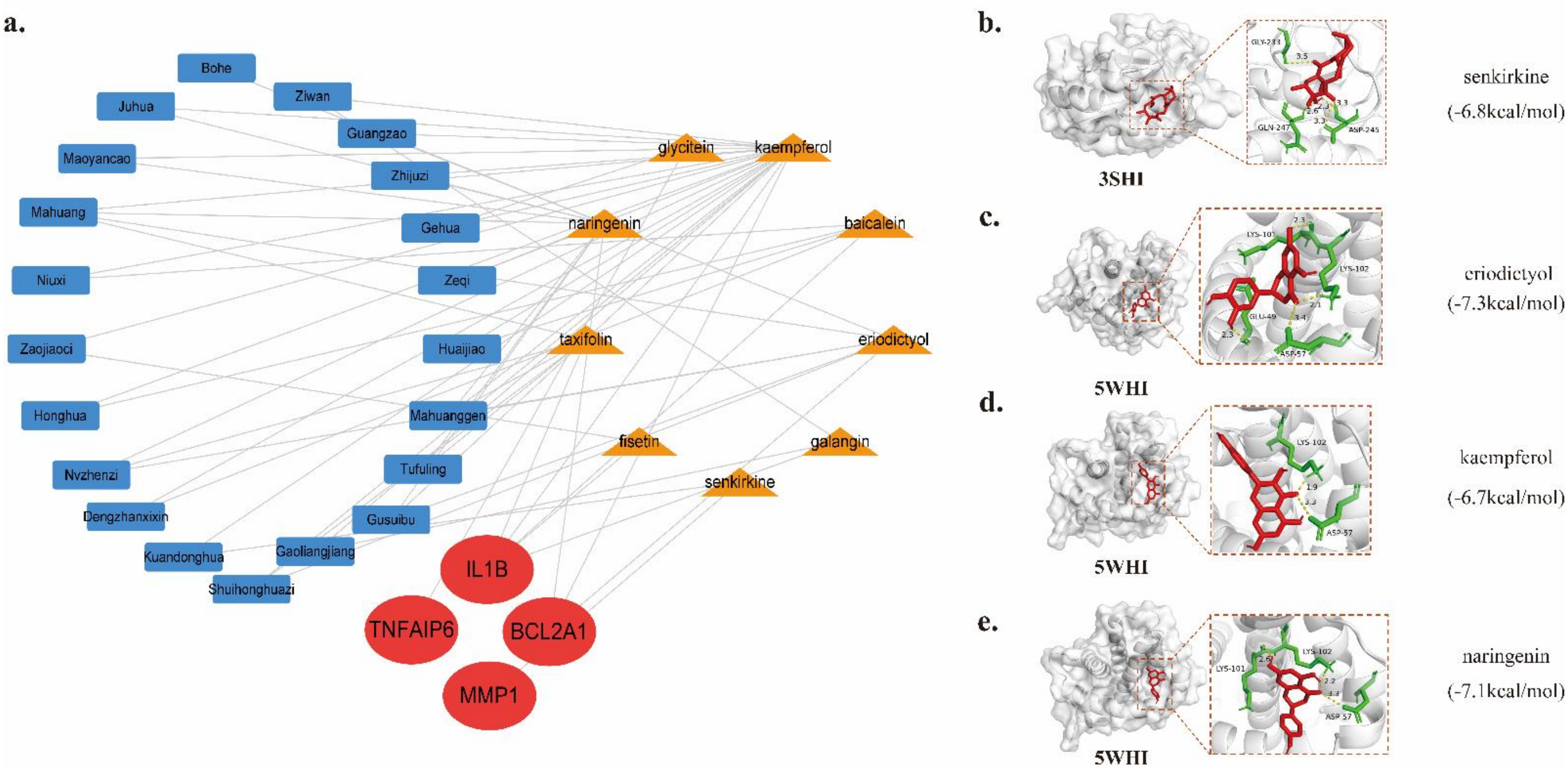

(a) Traditional Chinese Medicine-Compound-Target PPI Network Diagram. **(b)** Molecular Docking Diagram of 3SHI(*MMP1*) and senkirkine. **(c)** Molecular Docking Diagram of 5WHI(*BCL2A1*) and eriodictyol. **(d)** Molecular Docking Diagram of 5WHI(*BCL2A1*) and kaempferol. **(e)** Molecular Docking Diagram of 5WHI(*BCL2A1*) and naringenin.

4.Discussion

The precise mechanism underlying sJIA remains to be elucidated; it does not conform to the typical characteristics of an autoimmune disease and is frequently associated with systemic inflammatory manifestations [5]. Periodontal disease is a highly prevalent, multifactorial, chronic condition, and recent reports have indicated its association with a range of other autoimmune diseases [19,20,21]. The latent mechanisms underlying these diseases remain to be fully elucidated. In recent years, bioinformatics has emerged as a novel method to analyze and disclose the physiological mechanisms of various diseases. In this study, bioinformatics was used to discern the shared diagnostic biomarkers of PD and sJIA, offering novel approaches to prevent and treat these diseases.

In this study, we employed two analytical approaches, differential gene expression analysis and WGCNA, on GEO datasets for PD and sJIA to identify potential shared genes. An in-depth analysis of these shared genes has revealed their involvement in critical pathways, including Lipid and atherosclerosis, Coronavirus disease −COVID−19, the IL−17 signaling pathway, Rheumatoid arthritis, and the NF−kappa B signaling pathway. These pathways may play a pivotal role in the onset and progression of both diseases. Subsequent protein-protein interaction (PPI) analysis has determined the core hub genes that comprise the top ten. Subsequent to this, two algorithms for machine learning were employed in order to determine the genetic factors that underpin the co-morbid diagnoses *(FAM46C, SLEP, MS4A4A, BCL2A1, MMP1*). In the SHAP explanatory and external data verification process, the *SLEP* gene's evident diagnostic and predictive value for two diseases is demonstrated.In the immune infiltration analysis, both diseases exhibited higher neutrophil expression and lower CD8 T cell expression than the control group.These findings are consistent with SELP gene expression showing a positive correlation with neutrophils and a negative correlation with CD8+ T cells in both diseases.

SELP is a pivotal crossover gene between PD and SJIA, as it encodes the P-selectin protein (CD62P), which belongs to the selectin protein family. The molecule in question has been observed to be expressed on

stimulated endothelial cells and activated platelets. The interaction of the latter with P-selectin glycoprotein-1 (PSGL-1, CD162) has been shown to trigger a series of effects, including the rolling of leukocytes on the surface of stimulated endothelial cells and the aggregation of activated platelets on the surface of leukocytes, forming leukocyte aggregates [22]. In the course of immune infiltration analysis, it was observed that the immune responses in both diseases were positively correlated with the *SELP* gene in neutrophil function. In the course of immune infiltration analysis, it was observed that the immune responses in both diseases were positively correlated with the *SELP* gene in neutrophil function. *SELP* levels undergo a progressive increase during the progression of periodontitis, enhancing leukocyte recruitment to promote vascular injury, thereby exacerbating the severity of thrombotic and cardiovascular diseases [23]. Furthermore, *SELP* enrichment in the atherosclerosis pathway was observed during the KEGG enrichment analysis. A growing body of evidence suggests a close association between atherosclerosis and periodontitis, with the oral microbiota and humoral immune response potentially serving as key pathways linking the two conditions [24]. In addition, elevated levels of circulating P-selectin have been documented among cases of juvenile idiopathic arthritis (JIA) [25]. Research has demonstrated that PSGL-1, when stimulated by P-selectin, can induce the activation of integrins on the platelet surface. This, in turn, leads to the increased formation of intima-media thickening in the arteries [26,27]. Endothelial function is impaired at a very young age in JIA patients, and carotid intima-media thickness (IMT) is increased in sJIA patients [25]. This finding serves to further emphasise the heightened risk of atherosclerosis in patients diagnosed with sJIA. It can be seen that the *SELP* gene may link PD and sJIA through the atherosclerosis pathway.

We performed reverse engineering using the top ten hub genes to derive potential Chinese herbal compound components and determine the core diagnostic genes (*BCL2A1*, *MMP1*) and their corresponding compounds. We performed molecular docking and found that they exhibited good binding performance. We speculate that they may serve as a common treatment target for PD and sJIA.

BCL2A1 is a core diagnostic gene shared between periodontitis and systemic arthritis, and it is also one of the hub genes jointly identified in the protein-protein interaction (PPI) networks of both diseases. *BCL2A1*, a critical BCL-2 family anti-apoptosis protein, is dependent on NF-κB-mediated transcription, exhibiting dual functions of anti-apoptosis and inflammatory signal transduction. Evidence has demonstrated that *BCL2A1* plays a pivotal role in immune cell survival, inflammation, and tissue damage regulation [28]. According to the findings of preceding studies, *BCL2A1* is regarded as a pivotal gene in the context of periodontitis and is linked to mitochondrial immunity [29,30]. The function of *BCL2A1* in the regulation of cell survival and homeostasis is modulated by PI3K and JAK/STAT signals, thereby impacting inflammation [31]. In cases of sJIA, the excessive proliferation and activation of neutral cells may potentially compromise the integrity of the host organism [32].

MMP1, a member of the matrix metalloproteinase (MMP) family, plays a pivotal role in the degradation of the extracellular matrix and basement membranes during the initiation and progression of various diseases [33,34,35]. *MMP1* has been shown to degrade primary interstitial collagen fibres, including collagen types I and III, which are characterised by their resistance to the majority of proteases present within the periodontium[36]. Current research has confirmed its association with the pathogenesis of periodontitis [37,38].*MMP1* breaks down type II collagen in cartilage, and high levels of *MMP1* are found in arthritic tissue [39]. Heshuang Qu et al. found differences in *MMP1* expression between patients with active and/or inactive systemic juvenile idiopathic arthritis (sJIA) and healthy controls [40].

Conclusion

This study provides preliminary insights into the complex molecular interactions between PD and sJIA. It identifies *SELP* as the most significant potential key regulatory gene in both diseases. It also suggests potential therapeutic targets, such as *BCL2A1* and *MMP1*, and their binding to active components of traditional

Chinese medicine. Our findings deepened our understanding of the common immune mechanism and opened up new avenues for the development of targeted therapies, which in turn will improve the treatment of these chronic, inflammatory diseases.

Limitations

The present study is subject to certain limitations. Firstly, the analysis is based exclusively on publicly available transcription data sets. The potential for bias due to platform differences, population genetic variation and sample size limitations is a concern. Secondly, the validation of the reliability of the two key diagnostic genes has yet to be conducted through experimental verification in animals or cells. The future aim of the study is to collect patient samples to verify the expression of these genes in both diseases and to elucidate their function.

Acknowledgments

We would like to thank the researchers who made their microarray datasets publicly available, as well as my colleagues and advisor for their support.

References

1. Kwon T, Lamster IB, Levin L. Current Concepts in the Management of Periodontitis. Int Dent J. 2021 Dec;71(6):462-476. doi: 10.1111/idj.12630. Epub 2021 Feb 19.

2. Darveau RP. Periodontitis: a polymicrobial disruption of host homeostasis. Nat Rev Microbiol. 2010 Jul;8(7):481-90. doi: 10.1038/nrmicro2337.

3. Page RC, Schroeder HE. Pathogenesis of inflammatory periodontal disease. A summary of current work. Lab Invest. 1976 Mar;34(3):235-49.

4. Hajishengallis G, Chavakis T. Local and systemic mechanisms linking periodontal disease and inflammatory comorbidities. Nat Rev Immunol. 2021 Jul;21(7):426-440. doi: 10.1038/s41577-020-00488-6. Epub 2021 Jan 28.

5. Cimaz R. Systemic-onset juvenile idiopathic arthritis. Autoimmun Rev. 2016 Sep;15(9):931-4. doi: 10.1016/j.autrev.2016.07.004. Epub 2016 Jul 6.

6. Merle CL, Hoffmann R, Schmickler J, Rühlmann M, et al. Comprehensive Assessment of Orofacial Health and Disease Related Parameters in Adolescents with Juvenile Idiopathic Arthritis-A Cross-Sectional Study. J Clin Med. 2020 Feb 13;9(2):513. doi: 10.3390/jcm9020513.

7. Synodinos PN, Polyzois I. Oral health and orthodontic considerations in children with juvenile idiopathic arthritis: review of the literature and report of a case. J Ir Dent Assoc. 2008 Feb-Mar;54(1):29-36.

8. Kini V, Mohanty I, Telang G, Vyas N. Immunopathogenesis and distinct role of Th17 in periodontitis: A review. J Oral Biosci. 2022 Jun;64(2):193-201. doi: 10.1016/j.job.2022.04.005. Epub 2022 Apr 27.

9. Monasterio G, Castillo F, Rojas L, et al. Th1/Th17/Th22 immune response and their association with joint pain, imagenological bone loss, RANKL expression and osteoclast activity in temporomandibular joint osteoarthritis: A preliminary report. J Oral Rehabil. 2018 Aug;45(8):589-597. doi: 10.1111/joor.12649. Epub 2018 May 30.

10. Brasil-Oliveira R, Cruz ÁA, Sarmento VA, et al. Corticosteroid Use and Periodontal Disease: A Systematic Review. Eur J Dent. 2020 Jul;14(3):496-501. doi: 10.1055/s-0040-1713954. Epub 2020 Jul 1.

11. Langfelder P, Horvath S. WGCNA: an R package for weighted correlation network analysis. BMC Bioinformatics. 2008 Dec 29;9:559. doi: 10.1186/1471-2105-9-559.

12. Engebretsen S, Bohlin J. Statistical predictions with glmnet. Clin Epigenetics. 2019 Aug 23;11(1):123. doi: 10.1186/s13148-019-0730-1.

13. Xie Y, Shi H, Han B. Bioinformatic analysis of underlying mechanisms of Kawasaki disease via Weighted Gene Correlation Network Analysis (WGCNA) and the Least Absolute Shrinkage and Selection Operator method (LASSO) regression model. BMC Pediatr. 2023 Feb 24;23(1):90. doi: 10.1186/s12887-023-03896-4.

14. Valkenborg D, Rousseau AJ, Geubbelmans M, et al. Support vector machines. Am J Orthod Dentofacial Orthop. 2023 Nov;164(5):754-757. doi: 10.1016/j.ajodo.2023.08.003.

15. Newman AM, Liu CL, Green MR, et al. Robust enumeration of cell subsets from tissue expression profiles. Nat Methods. 2015 May;12(5):453-7. doi: 10.1038/nmeth.3337. Epub 2015 Mar 30.

16. Ru J, Li P, Wang J, et al. TCMSP: a database of systems pharmacology for drug discovery from herbal medicines. J Cheminform. 2014 Apr 16;6:13. doi: 10.1186/1758-2946-6-13.

17. Pinzi L, Rastelli G. Molecular Docking: Shifting Paradigms in Drug Discovery. Int J Mol Sci. 2019 Sep 4;20(18):4331. doi: 10.3390/ijms20184331.

18. Li C, Wen R, Liu D, Yan L, et al. Assessment of the Potential of Sarcandra glabra (Thunb.) Nakai. in Treating Ethanol-Induced Gastric Ulcer in Rats Based on Metabolomics and Network Analysis. Front Pharmacol. 2022 Jul 12;13:810344. doi: 10.3389/fphar.2022.810344.

19. Bolstad AI, Sehjpal P, Lie SA, Fevang BS. Periodontitis in patients with systemic lupus erythematosus: A nationwide study of 1,990 patients. J Periodontol. 2022 Mar;93(3):364-372. doi: 10.1002/JPER.21-0181. Epub 2021 Jun 16.

20. Kim JS, Lee IJ, Lee MG, et al. Periodontitis and risk of alopecia areata: A nationwide population-based cohort study in

Korea. J Eur Acad Dermatol Venereol. 2023 Mar;37(3):e397-e399. doi: 10.1111/jdv.18758. Epub 2022 Nov 25.

21. Dalmády S, Kemény L, Antal M, et al. Periodontitis: a newly identified comorbidity in psoriasis and psoriatic arthritis. Expert Rev Clin Immunol. 2020 Jan;16(1):101-108. doi: 10.1080/1744666X.2019.1700113. Epub 2019 Dec 11.

22. Chen M, Geng JG. P-selectin mediates adhesion of leukocytes, platelets, and cancer cells in inflammation, thrombosis, and cancer growth and metastasis. Arch Immunol Ther Exp (Warsz). 2006 Mar-Apr;54(2):75-84. doi: 10.1007/s00005-006-0010-6. Epub 2006 Mar 24.

23. Tonetti MS, D'Aiuto F, Nibali L,et al. Treatment of periodontitis and endothelial function. N Engl J Med. 2007 Mar 1;356(9):911-20. doi: 10.1056/NEJMoa063186.

24. Mendes R.T., Fernandes D. Endothelial dysfunction and periodontitis: The role of inflammatory serum biomarkers. Dent. Hypotheses. 2016;7:4. doi: 10.4103/2155-8213.177401.

25. Vlahos AP, Theocharis P, Bechlioulis A, et al. Changes in vascular function and structure in juvenile idiopathic arthritis. Arthritis Care Res (Hoboken). 2011 Dec;63(12):1736-44. doi: 10.1002/acr.20613.

26. Pircher J, Engelmann B, Massberg S, et al. Platelet-Neutrophil Crosstalk in Atherothrombosis. Thromb Haemost. 2019 Aug;119(8):1274-1282. doi: 10.1055/s-0039-1692983. Epub 2019 Jun 29.

27. Akhi R, Lavrinienko A, Hakula M, et al. Oral Microbiota Linking Humoral Response, Periodontitis and Atherosclerosis. J Clin Periodontol. 2025 Nov;52(11):1550-1559. doi: 10.1111/jcpe.70001. Epub 2025 Aug 12.

28. Silva-Gomes R, Mapelli SN, Boutet MA, et al. Differential expression and regulation of MS4A family members in myeloid cells in physiological and pathological conditions.J Leukoc Biol. 2022 Apr;111(4):817-836. doi: 10.1002/JLB.2A0421-200R. Epub 2021 Aug 4.

29. Lu H, Sun J, Sun J. Identification of potential crosstalk genes and mechanisms between periodontitis and diabetic nephropathy through bioinformatic analysis. Medicine (Baltimore). 2023 Dec 29;102(52):e36802. doi: 10.1097/MD.0000000000036802.

30. Sun X, Wu T, Yang Z, et al. Regulatory role of PDK1 via integrated gene analysis of mitochondria-immune response in periodontitis. Gene. 2024 Aug 5;918:148476. doi: 10.1016/j.gene.2024.148476. Epub 2024 Apr 22.

31. Vier J, Groth M, Sochalska M, et al. The anti-apoptotic Bcl-2 family protein A1/Bfl-1 regulates neutrophil survival and homeostasis and is controlled via PI3K and JAK/STAT signaling. Cell Death Dis. 2016 Feb 18;7(2):e2103. doi: 10.1038/cddis.2016.23.

32. Malengier-Devlies B, Metzemaekers M, Wouters C, et al. Neutrophil Homeostasis and Emergency Granulopoiesis: The Example of Systemic Juvenile Idiopathic Arthritis. Front Immunol. 2021 Dec 13;12:766620. doi: 10.3389/fimmu.2021.766620.

33. Ravanti L, Kähäri VM. Matrix metalloproteinases in wound repair (review). Int J Mol Med. 2000 Oct;6(4):391-407.

34. Nagase H, Woessner JF Jr. Matrix metalloproteinases. J Biol Chem. 1999 Jul 30;274(31):21491-4. doi: 10.1074/jbc.274.31.21491.

35. Birkedal-Hansen H, Moore WG, Bodden MK, et al. Matrix metalloproteinases: a review. Crit Rev Oral Biol Med. 1993;4(2):197-250. doi: 10.1177/10454411930040020401.

36. Visse R, Nagase H. Matrix metalloproteinases and tissue inhibitors of metalloproteinases: structure, function, and biochemistry. Circ Res. 2003 May 2;92(8):827-39. doi: 10.1161/01.RES.0000070112.80711.3D.

37. Lee W, Aitken S, Sodek J, et al. Evidence of a direct relationship between neutrophil collagenase activity and periodontal tissue destruction in vivo: role of active enzyme in human periodontitis. J Periodontal Res.1995 Jan;30(1):23-33. doi: 10.1111/j.1600-0765.1995.tb01249.x.

38. Sorsa T, Tjäderhane L, Salo T. Matrix metalloproteinases (MMPs) in oral diseases. Oral Dis. 2004 Nov;10(6):311-8. doi: 10.1111/j.1601-0825.2004.01038.x.

39. Vincenti MP, Brinckerhoff CE. Transcriptional regulation of collagenase(MMP-1, MMP-13) genes in arthritis: integration of complex signaling pathways for the recruitment of gene-specific transcription factors. Arthritis Res. 2002;4(3):157-64. doi: 10.1186/ar401. Epub 2001 Nov 23.

40. Qu H, Sundberg E, Aulin C, et al. Immunoprofiling of active and inactive systemic juvenile idiopathic arthritis reveals distinct biomarkers: a single-center study. Pediatr Rheumatol Online J. 2021 Dec 28;19(1):173. doi: 10.1186/s12969-021-00660-9.

Supporting information captions

S1 File. Traditional Chinese medicine – ingredients Information.

S2 File. Filter out the information of traditional Chinese medicine - ingredients- targets.

Click here to access/download
Supporting Information - Compressed/ZIP File Archive
Supporting information.rar
