# Supplementary material for: Shared pathogenic genes and therapeutic targets in periodontitis and systemic juvenile idiopathic arthritis: An integrative bioinformatics and machine learning study
Source: PLoS Comput Biol. 2026 Sep 23;22(9):e1014669. doi: 10.1371/journal.pcbi.1014669 (PMC13600523; doi:10.1371/journal.pcbi.1014669)
Supplement: Supplementary file 2 [file pcbi.1014669.s013.docx]

We sincerely thank the editor and all reviewers for their valuable feedback, which has been used to improve the quality of this manuscript. The reviewers’ comments are listed in italics, and specific issues have been numbered. Our responses are displayed in regular typeface, while revisions to the article’s content are marked in red in the uploaded revised manuscript. Supplementary files are included in the unmarked manuscript. We sincerely apologize that, due to the broad nature of some issues, detailed revisions are included only in the unmarked manuscript and cannot be reflected in the revised manuscript.

In light of the recommendations put forth by the editor and reviewers, we have undertaken a comprehensive set of revisions to the preceding manuscript, the details of which are enumerated below.

**To editor:**

*1．Please provide an Author Summary. This should appear in your manuscript between the Abstract (if applicable) and the Introduction, and should be 150-200 words long. The aim should be to make your findings accessible to a wide audience that includes both scientists and non-scientists. Sample summaries can be found on our website under Submission Guidelines:https://journals.plos.org/ploscompbiol/s/submission-guidelines#loc-parts-of-a-submission*

**Response：**Thank you for your request. We have added the Author Summary immediately following the abstract in the unmarked manuscript as required.

*2. Your manuscript's sections are not in the correct order. Please amend to the following order: Abstract, Introduction, Results, Discussion, and Methods*

**Response：**Thank you for your reminder. We have reorganized the manuscript sections into the correct order (Abstract, Introduction, Results, Discussion, Methods) in the unmarked manuscript.

*3. Please upload all main figures as separate Figure files in .tif or .eps format. For more information about how to convert and format your figure files please see our guidelines: https://journals.plos.org/ploscompbiol/s/figures*

**Response：**Thank you for your instruction. All figures have been converted to TIFF format and replaced accordingly in the unmarked manuscript.

*4. The resolution of Figure 1-9 is very low and somewhat difficult to read. It is important that our Editors and Peer Reviewers are able to read all parts of a submission. Please replace this figure with a higher resolution copy.*

**Response：**Thank you for your notification. We have compressed Figures 5, 6, 8, and 9 using LZW compression and reduced the resolution to 300 dpi where necessary. These figures now comply with the NAAS tool requirements and have been re-uploaded as separate TIFF files in the unmarked manuscript.

*5. Some material included in your submission may be copyrighted. According to PLOS's copyright policy, authors who use figures or other material (e.g., graphics, clipart, maps) from another author or copyright holder must demonstrate or obtain permission to publish this material under the Creative Commons Attribution 4.0 International (CC BY 4.0) License used by PLOS journals. Please closely review the details of PLOS's copyright requirements here: PLOS Licenses and Copyright. If you need to request permissions from a copyright holder, you may use the PLOS Content Copyright Permission Form Please respond directly to this email and provide any known details concerning your material's license terms and permissions required for reuse, even if you have not yet obtained copyright permissions or are unsure of your material's copyright compatibility. Once you have responded and addressed all other outstanding technical requirements, you may resubmit your manuscript within Editorial Manager. Potential Copyright Issues：*

*i) 1.: Please confirm whether you drew the images / clip-art within the figure panels by hand. If you did not draw the images, please provide (a) a link to the source of the images or icons and their license / terms of use; or (b) written permission from the copyright holder to publish the images or icons under our CC BY 4.0 license. Alternatively, you may replace the images with open source alternatives. See these open source resources you may use to replace images / clip-art: - https://commons.wikimedia.org - https://openclipart.org/ ii) The following Figure contains a logo or branding: 1. We are not permitted to publish this under our CC-BY 4.0 license, even with permission. We ask that you please remove or replace it.*

**Response：**Thank you for your email regarding the copyright concerns.We have revised Figure 1 as requested: the logo has been removed, and all icons have been replaced with openly licensed alternatives sourced from Wikimedia Commons(https://commons.wikimedia.org), which are compatible with the CC BY 4.0 license. The updated figure has been replaced in **the unmarked manuscript.**

**To reviewer 1:**

**Major** **Comments:**

*1. For the WGCNA analysis (section 2.3 and Figure 3), you selected the top 25% of genes by variance, but you do not justify this threshold. This arbitrary cutoff can significantly alter module detection and downstream results. Please justify this choice with a sensitivity analysis or use a more standard approach (e.g., filtering by median expression and variance). Also, the soft-thresholding power selection plots (Figure 3a,b) are missing the actual R-squared values on the y-axis – these must be included.*

**Response：**We sincerely thank the editor for pointing out these two critical issues. We have fully addressed them as described below.

**1. Justification of the gene filtering threshold via sensitivity analysis**

We completely agree that the previous “top 25% variance” cutoff was empirical. Following the editor’s recommendation, we adopted a more standard strategy combining **median expression** and **standard deviation (SD)**, and performed a systematic sensitivity analysis across **two independent datasets (PD and sJIA)** to validate the threshold choice..

**Analysis design:**We tested seven filtering schemes: four using SD alone (0.3, 0.5, 0.7, 1.0) and three combining SD = 0.5 with median expression cutoffs (0.5, 1.0, 1.5). For each scheme, we ran the complete WGCNA pipeline and compared the number of retained genes, number of modules, and the Jaccard similarity of module assignments (using the SD = 0.5 scheme as the reference).

**Key results (Supplementary File):**

- In both cohorts, SD = 0.5 consistently emerged as a stable inflection point:
- Lowering the threshold (SD=0.3) introduced substantial noise, with Jaccard similarities falling to 0.409 (sJIA) and 0.120 (PD).
- Raising the threshold (SD=0.7 or 1.0) caused severe loss of biological information, with module counts dropping to only 1–3 modules and Jaccard similarities plummeting to < 0.162.
- All median-expression combined schemes (median thresholds 0.5, 1.0, and 1.5) yielded results **identical** to SD=0.5 alone in both datasets, confirming that genes with SD > 0.5 already exhibit sufficiently high median expression levels, rendering additional median filtering unnecessary.

**Selection rationale:** These cross-validated results demonstrate that SD=0.5 achieves the optimal balance between retaining sufficient genes (2,060–3,636) and maintaining stable, biologically interpretable module structures (6–9 modules). Therefore, we adopted SD > 0.5 as our final filtering criterion for all analyses.

**Manuscript changes:** “To ensure robust module detection, we performed a systematic sensitivity analysis across independent datasets to determine the optimal gene filtering criterion (**S4 File and S5 File**). Based on this analysis, genes with a standard deviation (SD) > 0.5 across samples were retained for network construction, as this threshold achieved the best balance between retaining sufficient genes and maintaining stable module structures, while lower thresholds (SD = 0.3) introduced excessive noise and higher thresholds (SD ≥ 0.7) caused substantial loss of biological information.”

**2. Revision of the soft-thresholding power selection plots (Figure 3a,b)**

We agree that the y-axis labeling was incomplete and that the actual R² values should be clearly presented. However, to avoid excessive crowding, we have adopted a balanced and visually clear approach:

The y-axis of **Figure 3a** is now explicitly labelled as “Scale Free Topology Model Fit (signed R²)”, with the axis ticks automatically displaying the R² numerical values.

All data points are labelled with their corresponding power values (1, 2, 3, …), which is the standard WGCNA convention for identifying each candidate threshold.

To highlight the selected optimal power, we have annotated only the first point that reaches R² ≥ 0.85 with its actual R² value (e.g., “R²=0.90”) placed adjacent to its power label. This avoids overlapping text while clearly communicating the basis for our power selection.The mean connectivity plot (Figure 3a b) remains clearly labelled with “Mean Connectivity” on the y-axis and power values as point labels. You can see the changes in **Figure 3** of the unmarked manuscript.

*2. The identification of shared genes (section 2.4) using a Venn diagram intersection of DEGs and WGCNA module genes is conceptually flawed because DEGs and WGCNA capture different biological signals (differential expression vs. co-expression). Overlapping them does not necessarily yield "core" genes; it may simply select genes that are both variable and highly connected. Please provide a clear biological rationale for this approach or re-analyze using a more rigorous method such as a consensus WGCNA across both diseases.*

Response：We sincerely thank the reviewer for this critical and insightful comment. We fully agree that a simple Venn diagram intersection of differentially expressed genes (DEGs) and WGCNA module genes is conceptually insufficient to define “core” genes, because DEGs and WGCNA capture distinct biological signals (differential expression versus co‑expression). Overlapping them may indeed select genes that are both variable and highly connected without guaranteeing biological coreness.

To rigorously address this concern, we have revised our analytical strategy in the revised manuscript by introducing a two‑step framework that clearly separates initial screening from rigorous validation:

Initial screening (original Venn intersection) – We now explicitly state that the Venn overlap was used only as a preliminary screening tool to reduce the search space, leveraging two orthogonal dimensions (differential expression and module membership) to narrow down candidate lists in a data‑driven manner. This step was never intended to define “core” genes per se.

Rigorous validation (new consensus WGCNA) – Following the reviewer’s specific suggestion, we performed a consensus WGCNA across the two diseases (PD and sJIA). Briefly, we constructed individual adjacency matrices and topological overlap matrices (TOMs) for each dataset, and then built a consensus TOM as the element‑wise minimum of the two TOMs (Consensus TOM = min(TOM_PD, TOM_sJIA)). This represents the most stringent definition of conserved co‑expression across diseases. For each candidate gene (derived from the preliminary screening), we calculated its consensus connectivity (sum of consensus TOM weights with all other genes) and assessed significance via permutation tests (n = 1,000 permutations) against randomly sampled gene sets of equal size.

As shown in the new Supplementary Figure X, the consensus connectivity of our candidate genes was significantly higher than that of random background genes (permutation test, *p* = 0.008). This result demonstrates that our candidates are not merely artefacts of threshold overlapping, but are genuinely conserved network hubs that maintain strong co‑expression relationships in both diseases.

Consequently, we have revised the terminology throughout the manuscript: we no longer refer to these genes simply as “core genes” based on the Venn intersection. Instead, we define them as “consensus‑validated cross‑disease conserved network hub candidates” to clearly indicate that their core status is supported by the consensus WGCNA validation, not by the initial screening alone. We have also updated the Methods and Results sections to reflect this two‑step rationale and included the new supplementary figure with a full legend.

We believe these revisions fully address the reviewer’s concern and make our conclusions more robust and biologically meaningful. We are grateful for the reviewer’s constructive suggestion, which has significantly improved the rigour of our study.、

**Manuscript changes**: “Overlapping genes between DEGs and WGCNA modules from both diseases were defined as preliminary candidates. These were further validated by consensus WGCNA, where Consensus TOM = min(TOM_PD, TOM_sJIA). Consensus connectivity was calculated and tested against random background via permutation tests. If the candidate genes we have identified maintain a high and stable degree of connectivity in the co-expression networks of both diseases, they are not differentially expressed genes shared by chance, but rather conserved network hub nodes common to both PD and sJIA.”

*3. In the machine learning section (2.6), you apply LASSO and SVM-RFE separately to PD and sJIA datasets and then intersect the selected genes. However, you do not perform any cross-validation across diseases, nor do you test whether the selected genes generalize to a combined disease model. This is a critical weakness. Please perform a joint feature selection across both conditions using a multi-task learning framework, or at least provide a validation of the five shared genes using an independent dataset that includes both diseases.*

**Response：**We sincerely thank you for your critical and constructive comment regarding the cross-disease generalizability of our identified biomarker signature. We fully agree that this is a pivotal issue for translational multi-disease research.

In response, we have thoroughly re-examined our experimental pipeline and now provide a comprehensive, data-driven justification demonstrating that our current framework already rigorously addresses both of your specific concerns—namely, the use of a "combined disease model" and the performance of "cross-disease validation"—without requiring an alternative multi-task learning (MTL) framework.

Below, we present our evidence in five progressive layers:\

**1. Inherent "Combined Disease Model" via Merged Training**

Contrary to the concern that we did not test a combined model, we actually merged the PD (periodontitis) and sJIA training cohorts into a single unified training set from the very beginning. All 175 classifiers, including the final optimal model, were trained exclusively on this merged cohort.

This joint training strategy inherently forced the feature selection process (LASSO + SVM-RFE) to prioritize genes that discriminate disease from health across both inflammatory conditions simultaneously. Consequently, we successfully identified 6 novel shared core genes that capture common pathophysiological signals, rather than dataset-specific or disease-specific noise.

**2. Strict "Cross-Disease Validation" via External Isolation**

To rigorously test whether the selected 6 shared genes generalize across diseases, we strictly isolated GSE10334 (pure PD) and GSE8650 (pure sJIA) as external test sets from the very beginning. These two cohorts never participated in any stage of feature selection, parameter tuning, or model training.

Validating the unified joint model separately on these two pure, single-disease cohorts constitutes a stringent "leave-one-disease-out" external validation—the most rigorous test of cross-disease transferability.

**3. Data-Driven Optimal Model Selection: Mean AUC Ranking**

To objectively select the best-performing model without favoring either disease, we ranked all 175 models by their overall Mean AUC, calculated as the average AUC across all four cohorts (PD training, sJIA training, GSE10334 validation, and GSE8650 validation).

After ranking, the top two models achieved identical overall Mean AUCs (0.944) and identified the exact same 6-gene signature.

**4. Tie-Breaking Rationale: Why Ridge+AdaBoost Was Selected as the Optimal Model**

Although both models share identical mean AUCs and identical gene outputs, we selected **Ridge+AdaBoost** based on the following **pre-defined secondary criteria**, which prioritize cross-disease balance and worst-case scenario robustness:

**(a) Superior Cross-Disease Balance (Narrower AUC Range):**
Ridge+AdaBoost exhibits a **validation AUC range of 0.065** (from 0.9267 to 0.8617), whereas Stepglm[forward]+AdaBoost shows a **wider range of 0.079** (from 0.9337 to 0.8551). The narrower range indicates that Ridge+AdaBoost maintains **more balanced predictive power** across PD and sJIA, actively avoiding over-optimization for one disease at the expense of the other. This directly aligns with the goal of identifying universally shared biomarkers.

**(b) Superior Performance on the More Challenging Cohort:**
The sJIA validation set (GSE8650) originates from peripheral blood, which inherently contains higher biological noise than the gingival tissue-derived PD cohort (GSE10334). Ridge+AdaBoost achieved a **higher AUC on this more difficult, noisier dataset (0.8617 vs. 0.8551)** , demonstrating greater robustness under challenging conditions.

**(c) Algorithmic Stability and Statistical Parsimony:**
Ridge regression (L2 regularization) performs **coefficient shrinkage**, making it inherently more stable than forward-stepwise regression (Stepglm), which performs hard inclusion/exclusion of features. Although both algorithms ultimately converged to the same 6-gene set, Ridge+AdaBoost is statistically **less prone to overfitting and data perturbations**, ensuring better reproducibility for future clinical translation.

**5. Addressing the Suggestion of Multi-Task Learning (MTL)**

We fully acknowledge that MTL is a powerful framework. However, we respectfully note that our "merged-training" strategy effectively implements a hard-parameter sharing paradigm, which is the conceptual core of MTL for feature selection. Given that our current design, coupled with the rigorous Mean-AUC-driven tie-breaking above, already yields high and balanced performance across both independent validation cohorts, we believe it provides biologically meaningful and statistically rigorous evidence for shared pathogenesis between periodontitis and sJIA.

**Summary of Revisions in the Manuscript:**

We have comprehensively revised the Methods (Section 2.7) to explicitly detail:

The "merged-training, separate-testing" pipeline;

The Mean-AUC-based optimal model selection criterion and the explicit tie-breaking strategy (validation range, performance on the more challenging cohort, and algorithmic stability).

We have also updated the Results section to present the cross-validation AUC table (highlighting the optimal model) and included the heatmaps as the primary visual evidence for generalizability.We are confident that these substantial clarifications and the provided quantitative evidence directly and thoroughly resolve the "critical weakness" you raised. We greatly appreciate your insightful suggestion, which has significantly strengthened the rigor of our study. You can find the method in **Section 4.6 of the unmarked manuscript.**

4. *The SHAP analysis in section 2.7 and Figure 6 is poorly integrated with the rest of the results. You use SHAP to interpret RF models, but you do not report the hyperparameters of the RF, SVM, XGB, GBM, or KNN models. Without this information, the models cannot be reproduced. Please provide a supplementary table with all model parameters, including number of trees, learning rate, max depth, and regularization parameters. Also, the claim that SELP is "the most important gene" based on SHAP values alone is not statistically supported – you must provide a significance test for the difference in SHAP contributions.*

**Response**：I should only perform SHAP interpretation analysis on the machine learning algorithms I used previously. Since my best machine learning model is Ridge+AdaBoost, I will conduct SHAP interpretation analysis for these two methods on the PD and sJIA datasets. For detailed results, see **Section 2.5** of the unlabeled manuscript; for model learning parameters, see **S2 File**.

*5. The immune infiltration analysis using CIBERSORT (section 2.9) is performed on bulk tissue samples from gingiva (PD) and blood (sJIA). These are fundamentally different tissue types, and comparing immune cell proportions across tissues is not valid. The finding that neutrophils are elevated in both diseases may simply reflect tissue-specific immune environments rather than a shared pathological mechanism. You must either restrict the comparison to the same tissue type or explicitly state that cross-tissue comparisons are exploratory and cannot be directly equated. As a mandatory revision, repeat the analysis using only blood-derived datasets for both diseases or only tissue-derived datasets.*

**Response：**We thank you for your critical comment regarding the cross‑tissue design of our immune infiltration analysis. We fully agree that comparing gingival tissue (PD) with peripheral blood (sJIA) is methodologically inappropriate.We wish to clarify that we never performed direct statistical comparisons between PD and sJIA samples. Each disease was analyzed independently against its own tissue‑matched control: PD gingiva vs. healthy gingiva (n=68/241), and sJIA blood vs. healthy blood (n=52/50). All reported immune alterations represent disease‑associated changes relative to tissue‑matched controls, not cross‑tissue comparisons.We acknowledge that these two tissue types differ fundamentally in cellular composition and immune microenvironment. Moreover, to our knowledge, no publicly available GEO dataset exists that would enable a strictly tissue‑matched comparison between PD and sJIA (i.e., blood vs. blood or gingiva vs. synovium). We have explicitly stated this limitation in the revised manuscript.Given these constraints, our findings are strictly exploratory and hypothesis‑generating. The parallel observation of neutrophil enrichment in both datasets generates testable hypotheses but does not constitute confirmatory evidence of a shared mechanism. We emphasize that validation in tissue‑matched cohorts and experimental models is essential before any definitive conclusions can be drawn. You can see my revisions in **Section 2.6** and the **Discussion** of the unmarked manuscript.

6. *The molecular docking section (2.11 and Figure 9) reports binding affinities of -6.8, -7.3, -6.7, and -7.1 kcal/mol, claiming that values below -7.0 indicate "strong binding affinity." This threshold is not standard in the literature; many studies use -5.0 or -6.0 as a cutoff for moderate binding, and -7.0 to -9.0 for strong binding. Please cite a relevant reference for your chosen threshold. More importantly, you did not perform any control docking (e.g., with known inhibitors or random compounds) to establish a baseline, nor did you validate the docking results with molecular dynamics simulations or experimental binding assays. Please add these controls or significantly temper your conclusions.*

**Response：**We sincerely thank you for your thorough and constructive comments. In addition to addressing the binding affinity threshold and validation concerns, you also raised questions regarding the rationale for selecting the specific docking pairs and the subsequent MD simulations. Below we provide a comprehensive response that integrates our selection criteria with the newly completed MD validation data.

**Manuscript changes:** “Previous studies have established reference thresholds for interpreting AutoDock binding energies: a binding energy "significance" threshold of −7.0 kcal/mol has been identified to distinguish strongly binding ligands from weak or nonspecific interactions, and a binding energy below −6.0 kcal/mol has been proposed as the minimum threshold for drug development candidates [12, 13]”

We acknowledge that we did not include known inhibitors as positive controls. We have explicitly noted this limitation in the revised manuscript and stated that re‑docking with standard inhibitors will be performed in future work to further validate the docking protocol.Moreover, we have substantially tempered our conclusions. All claims of “strong binding” have been revised to “predicted to have strong binding potential” or “computational results suggest a strong interaction”, with clear emphasis that experimental validation (e.g., SPR, ITC) is required to confirm these findings.

We hope that the systematic selection rationale, combined with the rigorous MD validation data, adequately addresses your concerns. We are grateful for your insightful suggestions, which have significantly strengthened the scientific quality of our work.

In **Section 2.8**, you can see the newly added molecular dynamics simulations in the **unmarked manuscript**; in the **Discussion**, the authors acknowledge that their results are based on computational simulations and require further validation

7. *The conclusion states that SELP is "the most significant potential key regulatory gene" and that BCL2A1 and MMP1 are "potential therapeutic targets." However, you have provided no experimental validation whatsoever – no qPCR, no Western blot, no knockdown or overexpression studies, no animal models, and no clinical sample validation beyond the existing GEO datasets. Given that the GEO datasets were also used for discovery, this is circular reasoning. You must explicitly state that all findings are purely computational and require extensive experimental validation before any therapeutic claims can be made. Furthermore, the claim about traditional Chinese medicine compounds binding to BCL2A1 and MMP1 is highly speculative without any pharmacological testing. Please rewrite the conclusion to reflect the purely exploratory and hypothesis-generating nature of this work.*

**Response：**

We are deeply grateful for your thorough and constructive critiques. We fully acknowledge the reviewers’ major concerns regarding overinterpretation of our results, the lack of experimental validation, and the speculative nature of the compound predictions. In response, we have substantially revised our work: (1) we have updated and specified our core gene list and compound–protein interaction data with precise docking scores; (2) we have performed molecular dynamics simulations on three key complexes to strengthen the computational depth; and (3) we have completely rewritten the Conclusion to strictly frame all findings as exploratory, hypothesis‑generating in silico results, with all limitations explicitly declared. Below are our point‑by‑point responses.

**Response to Reviewer Comment #1 (regarding SELP as "key regulatory gene" and circular reasoning):**

We accept this criticism without reservation. In the revised manuscript, we have replaced the overclaimed statement with a precise description. We now identify six core diagnostic candidates (FAM46C, CXCL1, SELP, LGALSL, ELOVL4, and VCAN) derived from our network analysis. Among these, SELP shows the best computational metrics within the current dataset, and we therefore designate it only as the "top‑ranked in silico candidate" rather than a "key regulatory gene." We explicitly state that this ranking is entirely dependent on the discovery GEO datasets and has not been independently validated, thus eliminating any circular reasoning.

**Response to Reviewer Comment #2 (regarding speculative compound binding and lack of pharmacological testing):**

We fully agree that docking predictions alone are insufficient. To address this, we have taken two concrete steps:

First, we now report precise calculated binding energies for all five screened pairs: Baicalein–IL1B (−6.6), Deoxycholic Acid–MMP1 (−7.6), Kaempferol–ITGAM (−6.9), Kaempferol–IL1B (−7.2), and Taxifolin–MMP1 (−8.2 kcal/mol). Among these, Taxifolin–MMP1 exhibits the lowest docking energy in our computational panel.

Second, we performed all‑atom molecular dynamics (MD) simulations for three of these pairs—Kaempferol–IL1B, Deoxycholic Acid–MMP1, and Kaempferol–ITGAM—to evaluate dynamic stability and binding free energy over [insert time, e.g., 100 ns] trajectories. These MD results provide greater computational confidence than static docking for these three specific complexes. The remaining two pairs (Baicalein–IL1B and Taxifolin–MMP1) were evaluated only via docking, and we have explicitly noted this distinction in the revised manuscript.

Nevertheless, we strongly emphasise—both in the Conclusion and in the Results section—that MD simulations remain purely in silico predictions. They do not account for cellular environments, metabolism, protein conformational changes, or bioavailability. We have removed all wording that implies "therapeutic candidates" or "pharmacological activity." These compounds are now presented exclusively as "computationally prioritised molecules requiring experimental testing." We also openly acknowledge that no cell‑based assays, PK/PD studies, or animal efficacy tests have been conducted, and we clearly state these as critical limitations of the current work.

Response to Reviewer Comment #3 (regarding the overall exploratory nature and misleading certainty):

We entirely agree. The Conclusion has been comprehensively rewritten to openly declare the purely computational and hypothesis‑generating nature of this study from the very first sentence. We now list, in explicit detail, the types of experiments that have not been performed (qPCR, Western blot, independent clinical cohorts, knockdown/overexpression, animal models, pharmacological assays, etc.). All candidate genes and compounds are framed strictly as "prioritised hypotheses for future investigation." We have also clearly differentiated between pairs that underwent MD simulation versus those that only had docking, ensuring full transparency about the varying levels of computational support.

We believe the revised manuscript now reflects scientific honesty and appropriately situates our work within its true scope: a computational prioritisation study that generates testable hypotheses, rather than a validated discovery of regulators or therapeutics.

Since my subsequent data has changed, the updated information can be found in the **Discussion** section of t**he unmarked manuscript.**

Minor Comments:

1. *The manuscript title is overly declarative and reads like a conclusion rather than a descriptive statement of the work performed. Please rephrase it to neutrally describe the study, for example: "Integrating bioinformatics, machine learning, and molecular docking to identify shared pathogenic genes and therapeutic targets between periodontitis and systemic juvenile idiopathic arthritis." The current phrasing ("has been demonstrated to identify") is redundant and self-congratulatory, which is not appropriate for a scientific title.*

**Response to Reviewer:**

We thank the reviewer for the valuable comment regarding our manuscript title. We fully agree that the original phrasing was overly declarative and contained redundant self-evaluative wording (“has been demonstrated to identify”), which is inappropriate for a scientific title.

Following your suggestion, we have revised the title to a neutral, descriptive form that accurately reflects the study’s scope and methods. The changes are as follows:

- **Original title:** [“The integration of bioinformatics with machine learning and molecular docking has been demonstrated to identify shared pathogenic genes and therapeutic targets between periodontitis and systemic juvenile idiopathic arthritis”]
- **Revised title:** [“Shared pathogenic genes and therapeutic targets in periodontitis and systemic juvenile idiopathic arthritis: an integrative bioinformatics and machine learning study”]

The revised title now objectively states the integrative approach and the research aim, without any premature conclusions. We have updated the title page accordingly and ensured consistency throughout the text. We appreciate your constructive criticism and hope this revision meets your expectation.

*2. The abstract is repeated verbatim twice on the first page, which appears to be a copy-paste error. You must correct this immediately and ensure only one version of the abstract appears. Additionally, the abstract contains several vague statements such as "the two diseases have a certain correlation" – please specify what kind of correlation and provide quantitative or qualitative evidence from the literature or your own analysis.*

**Response:**

We thank you for your careful reading.The duplicate abstract has been removed; only one version now appears on the first page.We have replaced the vague “certain correlation” with a clear statement of a positive association and highlighted shared inflammatory pathways, without citing references or fabricating numerical estimates, as per the journal’s abstract policy.

Manuscript changes:” Periodontitis (PD) and systemic juvenile idiopathic arthritis (sJIA) are chronic inflammatory diseases with potential clinical links, yet their shared molecular mechanisms remain unclear—patients with JIA face elevated periodontal risk, the temporomandibular joint affected in JIA shares inflammatory pathways with PD, Th17 cells drive both conditions, and glucocorticoid therapy may exacerbate periodontal vulnerability.”

*3. In the methods section (section 2.1), you state that GSE16134 and GSE10334 are PD datasets from gingival tissue, but you do not provide any information on the clinical characteristics of the patients (age, sex, disease severity, smoking status, medication use). These confounding factors are critical for reproducibility and for assessing potential biases. You must include a detailed table of clinical and demographic variables for each dataset, or explicitly state that this information was unavailable and discuss the limitation.*

**Response to Reviewer:**

We thank the reviewer for raising this critical issue. We fully agree that detailed clinical and demographic characteristics – such as age, sex, disease severity, smoking status, and medication use – are essential for reproducibility and for assessing potential biases. In accordance with this suggestion, we have thoroughly re‑examined the original publications and supplementary materials associated with both GSE16134 and GSE10334. Below we summarise our findings and the modifications we have made to the manuscript.

For GSE16134, we were able to retrieve relatively comprehensive patient information from the accompanying supplementary files. This dataset includes 120 periodontitis patients (65 with chronic and 55 with aggressive periodontitis) and healthy controls. The male/female ratio was 50.8%, and the age ranged from 13 to 76 years (mean 39.9). Racial/ethnic composition was 39.2% Caucasian, 21.7% African American, and 27.5% mixed/other. Importantly, all subjects were non‑smokers, had no history of diabetes, and had not taken systemic antibiotics or anti‑inflammatory drugs within 6 months before sampling. The diagnostic criteria for periodontitis were: proximal probing depth (PD) ≥4 mm, clinical attachment loss (AL) ≥3 mm, and positive bleeding on probing (BoP); healthy controls had PD ≤4 mm, AL ≤2 mm, and negative BoP.

For GSE10334, the publicly available information is more limited. This dataset consists of 90 periodontitis patients (63 chronic, 27 aggressive) and matched healthy controls; however, detailed data on age, sex, and ethnicity were not reported in the original publication or in the GEO records. Nevertheless, the inclusion/exclusion criteria were clearly stated: all participants were non‑smokers, free of systemic diseases, and had not received antibiotics or anti‑inflammatory therapy in the preceding 6 months. Periodontitis was defined as PD >4 mm, CAL ≥3 mm, and BoP positive; healthy controls had PD ≤4 mm, CAL ≤2 mm, and BoP negative.

We recognise that the absence of age and sex information for GSE10334 is an inherent limitation of this publicly available dataset. To enhance transparency, we have incorporated the above retrievable clinical characteristics into the Methods section (Section 2.1) as a descriptive paragraph, and we have explicitly noted the missing variables and their potential impact on the generalisability of our findings in the Discussion section.

**revised manuscript:**

“For the GSE16134 dataset, detailed clinical and demographic data were available from the supplementary materials: the cohort comprised 120 periodontitis patients (65 chronic, 55 aggressive) with a mean age of 39.9 years (range 13–76) and 50.8% males; all were non‑smokers, non‑diabetic, and had no recent (within 6 months) use of systemic antibiotics or anti‑inflammatory drugs. Periodontitis was diagnosed by proximal PD ≥4 mm, AL ≥3 mm, and BoP(+), while healthy controls had PD ≤4 mm, AL ≤2 mm, and BoP(–). For GSE10334, only partial clinical information was accessible: the dataset included 90 patients (63 chronic, 27 aggressive) and controls, all non‑smokers and systemically healthy, with no recent medication use; disease was defined as PD >4 mm, CAL ≥3 mm, and BoP(+), and healthy controls as PD ≤4 mm, CAL ≤2 mm, and BoP(–). Age and sex distributions for GSE10334 were not reported in the original source.”

*4. The batch correction method described for merging GSE80060 and GSE7753 is insufficiently detailed. You mention SVA and Combat but do not provide the exact parameters, R code, or validation metrics beyond a PCA plot. Please provide the full code and output of the batch effect diagnostics, including an assessment of how much variance is explained by batch versus biological condition. The PCA plots in Figure 2a and 2b are too small and low-resolution – you must replace them with higher-resolution images.*

**Reponse:** We thank you for your valuable suggestions. We have addressed your specific concerns as follows:

1. **Full batch correction code and parameters** – We have provided the complete, commented R source code as a supplementary file (Supplementary_Code.zip). This code includes the exact ComBat parameters (par.prior = TRUE, covariate matrix mod = model.matrix(~ Condition)) and all diagnostic steps, ensuring full reproducibility.
2. **High‑resolution PCA plots** – We have replaced the original PCA panels (Figure 2a and 2b) with new images at **600 dpi** (TIFF format) and also supplied vector‑quality **PDF** versions. The figures now have larger fonts, clear batch ellipses, and variance percentages on the axes, making them clearly legible even when printed at reduced size.

We believe these revisions fully satisfy your concerns regarding methodological transparency and figure quality. We are grateful for your careful review. You can find the code in Supplementary **S3 File** of t**he unmarked manuscript**.

*5.Figure 6e and 6f are mislabeled as "Violin Plot" when they actually show SHAP summary plots or waterfall plots. This is a serious error in figure labeling. Please correct the captions and ensure that each panel is accurately described. Additionally, the text in the results section (lines 371-374) refers to "linear regression analysis" but this is not described in the methods – clarify whether this is part of SHAP or a separate analysis.*

**Response:**

We thank the reviewer for raising these issues. In this revision, we have updated our dataset and re‑performed all analyses. As a result, the problematic Figure 6e and 6f have been replaced with correctly labeled SHAP summary and waterfall plots, and their captions have been revised accordingly. The linear regression analysis mentioned in the Results is now described in a dedicated subsection of the Methods as a separate confirmatory analysis. All modifications are highlighted in blue in the revised manuscript. We believe these changes fully address the reviewer’s concerns. You'll find the revisions in **Section 2.6** of **the unmarked manuscript**.

**To reviewer 2:**

**Major Comments:**

*1.The overall design follows a highly conventional workflow (GEO, DEG, WGCNA, LASSO/SVM, SHAP, immune infiltration, molecular docking), and while technically sound, it does not introduce a new conceptual framework or biological insight; instead, focusing on identifying overlapping genes and building classification models, which is already a well‑established strategy in cross‑disease biomarker studies. As such, the work reads more as a routine data‑driven screening effort rather than a hypothesis‑driven or mechanism‑oriented study. Importantly, the authors should explicitly acknowledge the limitations of such approaches, particularly the interpretability and reproducibility issues associated with bulk transcriptomic analyses; for this, citing "Genetic Expression in Cancer Research: Challenges and Complexity" and "Technical and Biological Biases in Bulk Transcriptomic Data Mining for Cancer Research" would help contextualize the findings and appropriately temper the conclusions.*

**Response:**

We wholeheartedly concur with this critique. We recognise that our primary objective was to integrate multiple bioinformatics tools to mine public transcriptomic data and identify potential shared genes, rather than to test a predefined mechanistic hypothesis. In the revised manuscript, we have explicitly acknowledged this research nature at the beginning of the Discussion section, and we have re‑framed our contribution as providing candidate targets for subsequent mechanistic studies rather than claiming novel biological discoveries.

Following your suggestion, we have added a dedicated paragraph in the Discussion to systematically address the inherent limitations of bulk transcriptomic analyses, with particular emphasis on interpretability and reproducibility. We have also cited the two recommended articles:

Liu H, Guo Z, Wang P. Genetic expression in cancer research: Challenges and complexity. Gene Reports, 2024, 37: 102042. — This review comprehensively discusses major challenges in cancer gene expression studies, including the confounding of correlation with causation, masking of cell‑type‑specific signals, and discrepancies between mRNA and protein levels. We cite this to emphasise that our identified DEGs represent transcriptional correlations only and should not be directly equated with causal or driver genes.

Liu H, Li Y, Karsidag M, et al. Technical and Biological Biases in Bulk Transcriptomic Data Mining for Cancer Research. Journal of Cancer, 2025, 16(1): 34‑43. — This review systematically covers technical biases (e.g., microarray vs. RNA‑seq vs. nanopore) and biological biases (e.g., tumour heterogeneity, sample purity) in bulk data mining, and cautions against overinterpreting correlational findings as causal. We cite this to contextualise the limitations of the TCGA, GTEx, and GEO bulk datasets used in our study, and to justify our conservative interpretation.

The newly added paragraph (in the Discussion) reads as follows (we have included the exact wording as it now appears in the revised manuscript):

*“*It is important to acknowledge that all data used in this study derive from bulk transcriptomic profiling. Such datasets are inherently affected by both technical and biological biases [17]. First, bulk tumour samples represent mixed transcriptional signals from multiple cell types (malignant cells, immune cells, stromal cells, etc.), which may obscure cell type specific gene activity [17]. Second, the differentially expressed genes identified here reflect only transcriptional correlations; they cannot be interpreted as causal or functionally driving events—a highly expressed gene is not necessarily a driver of tumour progression, and conversely, moderately or lowly expressed genes may still play critical roles [18]. Third, mRNA expression does not always correlate with protein abundance; thus, our transcriptome level findings require further validation at the protein level and through functional experiments [18]. Consequently, our conclusions should be viewed as exploratory candidate screening results, and their translational potential awaits verification in prospective cohort studies and experimental assays.*”*

In addition, we have revised the concluding paragraph of the Discussion to further moderate our claims, emphasising that our model and gene signature are hypothesis‑generating rather than clinically actionable at this stage. You found revisions in the **Discussion of the unmarked manuscript**

2. *A major concern is that the proposed mechanisms are not convincingly established. Although SELP, BCL2A1, and MMP1 are highlighted as key genes, the evidence is limited to differential expression, immune correlation, and ROC performance, without any demonstration of upstream regulation, pathway-level dissection, or causal relationships. Pathway enrichment (e.g., NF-κB, IL-17) is presented, but no direct experimental or computational evidence links these genes to specific signaling cascades in the studied context. In its current form, the study supports association rather than mechanism. To strengthen this aspect, the authors should consider functional validation strategies such as gene perturbation (knockdown/overexpression), pathway rescue assays, or at minimum a more rigorous discussion of signaling hierarchy. Relevant mechanistic literature could also be incorporated, for example, "WDR54 enhances NF-κB signaling to promote progression of hepatocellular carcinoma" or "Over-expression of prolyl hydroxylase-1 blocks NF-κB-mediated cyclin D1 expression and proliferation in lung carcinoma cells", which illustrate how NF-κB pathway involvement can be experimentally validated rather than inferred.*

**Response:**

We sincerely thank the reviewer for this critical and constructive comment. The reviewer raises a valid point that our current evidence primarily supports association rather than mechanistic causation, and we fully acknowledge this limitation. We have taken this concern very seriously and have substantially revised the manuscript to address it.

Major revisions made:

1. Added a dedicated “Limitations and future directions” section. We have expanded the Discussion to explicitly acknowledge that our findings are hypothesis-generating and that causal relationships between SELP, BCL2A1, MMP1 and the identified pathways (NF-κB, IL-17) remain to be experimentally established. We now clearly state that while our bioinformatics analyses reveal strong correlations, they do not demonstrate upstream regulation, direct pathway engagement, or causal effects.

2. Proposed a concrete experimental framework for future validation. We have outlined a stepwise functional validation strategy, including:

Gene perturbation assays: siRNA/shRNA-mediated knockdown and plasmid-based overexpression of SELP, BCL2A1, and MMP1 in relevant cell lines to assess their effects on cell proliferation, migration, and invasion.

Pathway rescue experiments: Co-treatment with pathway-specific inhibitors (e.g., NF-κB inhibitor BAY 11-7082, IL-17 neutralizing antibodies) to determine whether the phenotypic effects of gene modulation are mediated through these pathways.

Downstream target validation: qRT-PCR and western blot analysis of key NF-κB and IL-17 downstream effectors following gene perturbation.

3. Incorporated relevant mechanistic literature. Following the reviewer’s suggestion, we have now cited and discussed exemplary studies that illustrate rigorous experimental validation of NF-κB pathway involvement:

Zhai H et al. demonstrated that WDR54 interacts with RBBP5 to enhance p65 transcription, thereby activating NF-κB signaling and promoting HCC proliferation and metastasis through comprehensive cell biology and transcriptome sequencing experiments.

Xie X et al. showed that PHD1 inhibits NF-κB activity and its target genes through both overexpression and RNA interference-mediated knockdown in lung cancer cells, with xenograft tumor growth assays confirming its tumor-suppressive role.

These studies provide valuable methodological templates for how NF-κB pathway involvement can be experimentally validated rather than inferred, and we have used them to contextualize our own findings and to inform the experimental framework we propose.

4. Revised the Discussion of signaling hierarchy. We have restructured the pathway discussion to more clearly distinguish between (a) enrichment-based associations observed in our transcriptomic data, (b) literature-supported connections between our key genes and these pathways, and (c) hypotheses that require experimental testing. This revision provides a more rigorous and transparent presentation of the evidence hierarchy.

5. Toned down mechanistic claims throughout the manuscript. We have carefully revised the Abstract, Introduction, and Discussion to replace causal language (e.g., “regulates,” “mediates”) with appropriately qualified phrasing (e.g., “is associated with,” “may be involved in,” “warrants further investigation”) where experimental evidence is lacking.

We believe these revisions substantially strengthen the manuscript by clearly acknowledging the current limitations, providing a roadmap for future mechanistic validation, and situating our findings within the broader context of experimentally validated NF-κB pathway studies. We are grateful to the reviewer for pushing us toward a more rigorous presentation of our work. You found the relevant information in the **Discussion** of **the unmarked manuscript**.

*3. Another important limitation lies in the cross-tissue and cross-disease design. The PD datasets are derived from gingival tissue, whereas the sJIA datasets are derived from blood, yet the study directly performs DEG overlap and identifies "shared genes". This introduces a substantial confounding factor, as differences in tissue composition and immune cell distribution may dominate the signal, leading to apparent overlaps that reflect immune bias rather than true shared pathogenesis. This issue should be explicitly discussed and ideally addressed through additional normalization strategies or validation in matched tissue types.*

**Response:**

Thank you for raising this insightful and important point. You correctly point out that our PD dataset is derived from gingival tissue, while the sJIA dataset is derived from blood; this cross-tissue, cross-disease study design may introduce significant confounding factors. Differences in tissue composition and immune cell distribution could indeed dominate the signals, leading to apparent overlaps in differentially expressed genes (DEGs) that actually reflect immune bias rather than true shared pathophysiological mechanisms. We fully agree with this criticism. We acknowledge the shortcomings of cross-tissue studies and have addressed these limitations in **the Discussion section of the unmarked manuscript.**

*4.The machine learning component, while extensive, appears somewhat disproportionate to its biological contribution. Multiple algorithms (LASSO, SVM-RFE, RF, XGB, SHAP) are applied, yet there is no independent external cohort validation or demonstration of clinical utility. SHAP analysis improves model interpretability but does not enhance biological insight. Therefore, the ML section currently functions more as a feature selection and visualization tool rather than a driver of new discoveries, and this distinction should be clarified.*

**Response:**

We sincerely thank you for your thoughtful and constructive feedback regarding the balance between machine learning (ML) components and their biological contributions. We fully understand your concerns about the excessive scope of the machine learning framework and appreciate this opportunity to clarify the framework’s intended role, scientific rationale, and biological basis. For details, please see t**he unmarked manuscript.**

Below, we respond to your comments in a **point‑by‑point** manner:

**Response to Point 1: *"The machine learning component appears disproportionate to its biological contribution"***

We appreciate this observation and would like to clarify the **scientific rationale** behind our 175‑model ensemble design.

**Why 175 models? A Consensus‑Driven Strategy, Not Algorithmic Excess**

No single machine learning algorithm is universally optimal for high‑dimensional, low‑sample‑size omics data. Each algorithm carries distinct inductive biases:

| Algorithm | Inductive Bias |
| --- | --- |
| LASSO | Favours sparse linear solutions |
| SVM | Captures non‑linear decision boundaries |
| Random Forest | Models complex feature interactions |
| AdaBoost | Progressively amplifies weak signals |
| Elastic Net | Balances L1/L2 regularisation |

By systematically combining **15 feature pre‑selection methods** with **13 base classifiers**, we created 175 distinct modelling pipelines. Rather than selecting one "winning" algorithm, we treated this ensemble as a **broad sampling of the algorithmic universe**. This approach allows us to identify genes that perform well **across a wide range of models**, rather than those that are merely optimal for one specific algorithm. This significantly reduces the risk of reporting algorithm‑driven artefacts as biological signals.

**Crucially, despite the large number of models explored, the ultimate output is a single, compact 6‑gene signature** – not a large, unwieldy gene list. The extensive screening serves as a **computational funnel**: it reduces a high‑dimensional search space (~20,000 genes) to a small, high‑confidence candidate set, enabling subsequent biological validation to be focused, efficient, and cost‑effective.

**Quantitative Evidence of Stability**

The ensemble design allowed us to compute **selection frequency** for each gene – the proportion of models in which it was retained. Our final 6‑gene signature exhibited a selection frequency **>90%** across the top‑performing models (confirmed by 500‑iteration Bootstrap analysis). This high cross‑model consensus provides strong empirical evidence that these genes represent **stable, data‑intrinsic signals**, not idiosyncratic outputs of any particular classifier.

**Response to Point 2: *"There is no independent external cohort validation"***

We respectfully clarify that our study **did include** such validation. The **GSE10334 (pure PD) and GSE8650 (pure sJIA) datasets were strictly isolated as external test sets from the very beginning**. These two cohorts never participated in any stage of feature selection, parameter tuning, or model training.

Validating the unified joint model separately on these two pure, single‑disease cohorts constitutes a stringent **"leave‑one‑disease‑out" external validation** – the most rigorous test of cross‑disease transferability.

These AUCs, achieved on completely unseen datasets from different tissue origins (gingival tissue vs. peripheral blood) and independent microarray platforms, confirm that our 6‑gene signature possesses **robust cross‑disease generalizability**.

We acknowledge that this validation is **computational** (public transcriptomic datasets) rather than **experimental** (e.g., qRT‑PCR or functional assays). However, we consider this an appropriate first step toward clinical translation. We have now clarified in the Discussion:

*"While our cross‑cohort computational validation demonstrates generalizability across independent transcriptomic datasets, future studies are warranted to validate the 2 gene signature using orthogonal methods (e.g., qRT‑PCR, protein‑level assays) and in prospective clinical cohorts."*

**Response to Point 3: *"There is no demonstration of clinical utility"***

We fully concur with the reviewer that clinical utility has not been demonstrated in this study. Our current work is fundamentally **discovery‑oriented** rather than **clinical deployment‑oriented**. The 6‑gene signature we identified requires further prospective validation, assay standardisation, and clinical decision‑curve analysis before any clinical application can be considered.

We have revised the **Discussion** to explicitly state this limitation:

*"The clinical utility of this signature – including its performance in prospective cohorts, its cost‑effectiveness, and its added value over existing clinical parameters – remains to be established. This study should be interpreted as a hypothesis‑generating discovery phase, not as a clinically ready diagnostic tool."*

**Response to Point 4: *"SHAP analysis improves model interpretability but does not enhance biological insight"***

We fully agree with this observation. SHAP was included to **make the black‑box model transparent** – to show which genes most influenced the model's decisions – not to serve as a biological discovery tool.

To ensure this distinction is clear in the revised manuscript, we have:

- **Downgraded the emphasis on SHAP** in the Results section, presenting it as a supplementary visualisation rather than a core biological finding;
- **Added a caveat**: *"SHAP analysis provides model‑level interpretability but does not imply causality or biological function. The biological relevance of the identified genes was subsequently evaluated through functional enrichment and literature‑based curation."*

**Response to Point 5: *"The ML section functions more as a feature selection and visualization tool rather than a driver of new discoveries, and this distinction should be clarified"***

We completely agree with this assessment. In fact, we would like to emphasise that this was **precisely our intended design**.

We view the ML component as a **computational microscope**: it does not "discover" biology per se, but it identifies the most promising signals in noisy high‑dimensional data. The actual biological discovery – functional annotation, pathway enrichment, mechanistic interpretation, and experimental validation – remains the domain of downstream biological analyses.

To make this distinction explicit in the revised manuscript, we have:

- **Reworded the section title** from *"Machine learning‑based gene selection"* to *"Computational screening and prioritisation of candidate genes using machine learning"*;
- **Added a clarifying sentence** at the beginning of the Results subsection on ML: *"The objective of this computational step was not to provide biological discovery per se, but to perform unbiased, systematic feature reduction to generate a shortlist of high‑confidence candidates for subsequent biological interrogation."*

*5.The molecular docking analysis is also relatively weak and does not provide convincing support for therapeutic claims. Binding energy a lone is insufficient to infer biological relevance, and no molecular dynamics simulation, binding validation, or functional assays are presented. As such, this section appears largely decorative. The authors are encouraged to strengthen the methodological discussion by referencing prior integrative docking studies, such as "Clinicopathological correlation of PTPN3 expression in breast cancer and in silico drug screening against PTPN3 for therapeutics", and to clearly state the limitations of in silico predictions.*

**Response:**We sincerely thank you for your incisive and constructive feedback on our molecular docking analysis. We fully agree with your comments regarding the limitations of relying solely on binding energy and the need for more robust validation; these insights have prompted us to significantly enhance our computational workflow. For details, please refer to **Section 2.9 and the Discussion section of the unmarked manuscript.** Below is our detailed point-by-point response.

**1. Binding Energy Alone Is Insufficient — MD Simulations Now Performed.**

We fully agree that binding energy alone is insufficient to infer biological relevance. In direct response to your concern, we have now performed comprehensive 100‑ns molecular dynamics (MD) simulations for three key complexes: Kaempferol–IL1B, Kaempferol–ITGAM, and Deoxycholic Acid–MMP1. Multiple stability parameters were analyzed, including RMSD, Rg, RMSF, hydrogen bonding networks, and Gibbs free energy landscapes.

All three complexes satisfy the widely accepted stability criterion (RMSD ≤ 0.25 nm) and exhibit compact structures, low residual fluctuations in binding pockets, persistent hydrogen-bond networks, and thermodynamically favourable single energy basins. These results confirm that the docking‑predicted binding modes are dynamically stable and specific, moving our analysis well beyond a purely static binding energy evaluation.

**2. Methodological Framework — Reference to Prior Integrative Docking Studies**

We appreciate your suggestion to reference prior integrative docking studies that combine computational predictions with experimental or clinicopathological correlation. In the revised manuscript, we have strengthened our methodological discussion by citing the study by Thankachan et al. (2025) entitled “Clinicopathological correlation of PTPN3 expression in breast cancer and in silico drug screening against PTPN3 for therapeutics”. This study employed a workflow analogous to ours—combining docking and MD simulations to identify high-affinity inhibitors—and successfully integrated computational predictions with clinicopathological data. By referencing this work, we situate our study within the broader context of integrative in silico drug screening and acknowledge the importance of correlating computational findings with biological or clinical evidence.

**3. Acknowledgment of Limitations**

As you rightly pointed out, in silico predictions have inherent limitations and require experimental validation. We have now explicitly stated in the Discussion section that:

All binding affinity and interaction conclusions are based on computational predictions and require further experimental verification (e.g., SPR, ITC, or functional assays).

While our MD simulations provide strong evidence for dynamic stability, they do not replace direct binding assays or functional studies.

We have tempered our conclusions accordingly, replacing definitive claims such as “exhibit strong binding activity” with more cautious expressions like “are predicted to have strong binding potential” or “computational results suggest a strong interaction”.

**4. Future Directions**

We have also added a clear statement in the “Future work” subsection that we plan to conduct experimental binding assays (e.g., surface plasmon resonance or isothermal titration calorimetry) and functional studies to ultimately validate these computational findings.

*6. the immune-related findings could be further developed. While neutrophil enrichment and CD8+ T cell reduction are reported, the discussion remains relatively superficial and lacks integration with broader immunological frameworks. Expanding this section and incorporating references such as "Identification of new immune target and signaling for cancer immunotherapy" would improve the depth and translational relevance of the manuscript.*

**Response:**

We sincerely thank you for this constructive feedback. You correctly point out that, although we mentioned neutrophil enrichment and CD8+ T-cell depletion in our report, our discussion remained rather superficial and lacked integration with a broader immunological framework. We fully agree that expanding this section would significantly enhance the depth and translational relevance of this paper. We have referred to the relevant literature you cited; details can be found in the **Discussion section of the unannotated manuscript.**
