# Supplementary material for: Shared pathogenic genes and therapeutic targets in periodontitis and systemic juvenile idiopathic arthritis: An integrative bioinformatics and machine learning study
Source: PLoS Comput Biol. 2026 Sep 23;22(9):e1014669. doi: 10.1371/journal.pcbi.1014669 (PMC13600523; doi:10.1371/journal.pcbi.1014669)
Supplement: Peer review history

# Peer review: Decision Letter 0

**DOI:** 10.1371/journal.pcbi.1014669.r001

**Authors:** Can Yang, Muhammad Naseem

30 Apr 2026

PCOMPBIOL-D-26-00673

The integration of bioinformatics with machine learning and molecular docking has been demonstrated to identify shared pathogenic genes and therapeutic targets between periodontitis and systemic juvenile idiopathic arthritis

PLOS Computational Biology

Dear Authors,

Thank you for submitting your manuscript to PLOS Computational Biology. After careful consideration, we feel that it has merit but does not fully meet PLOS Computational Biology’s publication criteria as it currently stands. Therefore, we invite you to submit a revised version of the manuscript that addresses the points raised during the review process.

Please submit your revised manuscript by 19/05/2026. If you will need more time than this to complete your revisions, please reply to this message or contact the journal office at ploscompbiol@plos.org. When you’re ready to submit your revision, log on to https://www.editorialmanager.com/pcompbiol/ and select the 'Submissions Needing Revision' folder to locate your manuscript file.

Please include the following items when submitting your revised manuscript:

* A letter that responds to each point raised by the editor and reviewer(s). You should upload this letter as a separate file labeled 'Response to Reviewers'. This file does not need to include responses to formatting updates and technical items listed in the 'Journal Requirements' section below.

* A marked-up copy of your manuscript that highlights changes made to the original version. You should upload this as a separate file labeled 'Revised Manuscript with Track Changes'.

* An unmarked version of your revised paper without tracked changes. You should upload this as a separate file labeled 'Manuscript'.

If you would like to make changes to your financial disclosure, competing interests statement, or data availability statement, please make these updates within the submission form at the time of resubmission. Guidelines for resubmitting your figure files are available below the reviewer comments at the end of this letter

We look forward to receiving your revised manuscript.

Kind regards,

Muhammad Naseem, PhD

Academic Editor

PLOS Computational Biology

Can Yang

Section Editor

PLOS Computational Biology

**Journal Requirements:**

If the reviewer comments include a recommendation to cite specific previously published works, please review and evaluate these publications to determine whether they are relevant and should be cited. There is no requirement to cite these works unless the editor has indicated otherwise.

1) Please provide an Author Summary. This should appear in your manuscript between the Abstract (if applicable) and the Introduction, and should be 150-200 words long. The aim should be to make your findings accessible to a wide audience that includes both scientists and non-scientists. Sample summaries can be found on our website under Submission Guidelines:

https://journals.plos.org/ploscompbiol/s/submission-guidelines#loc-parts-of-a-submission

2) Your manuscript's sections are not in the correct order.  Please amend to the following order: Abstract, Introduction, Results, Discussion, and Methods

3) Please upload all main figures as separate Figure files in .tif or .eps format. For more information about how to convert and format your figure files please see our guidelines:

https://journals.plos.org/ploscompbiol/s/figures

4) The resolution of Figure 1-9 is very low and somewhat difficult to read. It is important that our Editors and Peer Reviewers are able to read all parts of a submission. Please replace this figure with a higher resolution copy.

5) Some material included in your submission may be copyrighted. According to PLOS's copyright policy, authors who use figures or other material (e.g., graphics, clipart, maps) from another author or copyright holder must demonstrate or obtain permission to publish this material under the Creative Commons Attribution 4.0 International (CC BY 4.0) License used by PLOS journals. Please closely review the details of PLOS's copyright requirements here: PLOS Licenses and Copyright. If you need to request permissions from a copyright holder, you may use the PLOS Content Copyright Permission Form Please respond directly to this email and provide any known details concerning your material's license terms and permissions required for reuse, even if you have not yet obtained copyright permissions or are unsure of your material's copyright compatibility. Once you have responded and addressed all other outstanding technical requirements, you may resubmit your manuscript within Editorial Manager. Potential Copyright Issues:

i) 1.: Please confirm whether you drew the images / clip-art within the figure panels by hand. If you did not draw the images, please provide (a) a link to the source of the images or icons and their license / terms of use; or (b) written permission from the copyright holder to publish the images or icons under our CC BY 4.0 license. Alternatively, you may replace the images with open source alternatives. See these open source resources you may use to replace images / clip-art: - https://commons.wikimedia.org - https://openclipart.org/ ii) The following Figure contains a logo or branding: 1. We are not permitted to publish this under our CC-BY 4.0 license, even with permission. We ask that you please remove or replace it.

**Reviewers' comments:**

Reviewer's Responses to Questions

**Comments to the Authors:**

**Please note here if the review is uploaded as an attachment.**

Reviewer #1: 1. The manuscript title is overly declarative and reads like a conclusion rather than a descriptive statement of the work performed. Please rephrase it to neutrally describe the study, for example: "Integrating bioinformatics, machine learning, and molecular docking to identify shared pathogenic genes and therapeutic targets between periodontitis and systemic juvenile idiopathic arthritis." The current phrasing ("has been demonstrated to identify") is redundant and self-congratulatory, which is not appropriate for a scientific title.

2. The abstract is repeated verbatim twice on the first page, which appears to be a copy-paste error. You must correct this immediately and ensure only one version of the abstract appears. Additionally, the abstract contains several vague statements such as "the two diseases have a certain correlation" – please specify what kind of correlation and provide quantitative or qualitative evidence from the literature or your own analysis.

3. In the methods section (section 2.1), you state that GSE16134 and GSE10334 are PD datasets from gingival tissue, but you do not provide any information on the clinical characteristics of the patients (age, sex, disease severity, smoking status, medication use). These confounding factors are critical for reproducibility and for assessing potential biases. You must include a detailed table of clinical and demographic variables for each dataset, or explicitly state that this information was unavailable and discuss the limitation.

4. The batch correction method described for merging GSE80060 and GSE7753 is insufficiently detailed. You mention SVA and Combat but do not provide the exact parameters, R code, or validation metrics beyond a PCA plot. Please provide the full code and output of the batch effect diagnostics, including an assessment of how much variance is explained by batch versus biological condition. The PCA plots in Figure 2a and 2b are too small and low-resolution – you must replace them with higher-resolution images.

5. For the WGCNA analysis (section 2.3 and Figure 3), you selected the top 25% of genes by variance, but you do not justify this threshold. This arbitrary cutoff can significantly alter module detection and downstream results. Please justify this choice with a sensitivity analysis or use a more standard approach (e.g., filtering by median expression and variance). Also, the soft-thresholding power selection plots (Figure 3a,b) are missing the actual R-squared values on the y-axis – these must be included.

6. The identification of shared genes (section 2.4) using a Venn diagram intersection of DEGs and WGCNA module genes is conceptually flawed because DEGs and WGCNA capture different biological signals (differential expression vs. co-expression). Overlapping them does not necessarily yield "core" genes; it may simply select genes that are both variable and highly connected. Please provide a clear biological rationale for this approach or re-analyze using a more rigorous method such as a consensus WGCNA across both diseases.

7. In the machine learning section (2.6), you apply LASSO and SVM-RFE separately to PD and sJIA datasets and then intersect the selected genes. However, you do not perform any cross-validation across diseases, nor do you test whether the selected genes generalize to a combined disease model. This is a critical weakness. Please perform a joint feature selection across both conditions using a multi-task learning framework, or at least provide a validation of the five shared genes using an independent dataset that includes both diseases.

8. The SHAP analysis in section 2.7 and Figure 6 is poorly integrated with the rest of the results. You use SHAP to interpret RF models, but you do not report the hyperparameters of the RF, SVM, XGB, GBM, or KNN models. Without this information, the models cannot be reproduced. Please provide a supplementary table with all model parameters, including number of trees, learning rate, max depth, and regularization parameters. Also, the claim that SELP is "the most important gene" based on SHAP values alone is not statistically supported – you must provide a significance test for the difference in SHAP contributions.

9. Figure 6e and 6f are mislabeled as "Violin Plot" when they actually show SHAP summary plots or waterfall plots. This is a serious error in figure labeling. Please correct the captions and ensure that each panel is accurately described. Additionally, the text in the results section (lines 371-374) refers to "linear regression analysis" but this is not described in the methods – clarify whether this is part of SHAP or a separate analysis.

10. The immune infiltration analysis using CIBERSORT (section 2.9) is performed on bulk tissue samples from gingiva (PD) and blood (sJIA). These are fundamentally different tissue types, and comparing immune cell proportions across tissues is not valid. The finding that neutrophils are elevated in both diseases may simply reflect tissue-specific immune environments rather than a shared pathological mechanism. You must either restrict the comparison to the same tissue type or explicitly state that cross-tissue comparisons are exploratory and cannot be directly equated. As a mandatory revision, repeat the analysis using only blood-derived datasets for both diseases or only tissue-derived datasets.

11. The molecular docking section (2.11 and Figure 9) reports binding affinities of -6.8, -7.3, -6.7, and -7.1 kcal/mol, claiming that values below -7.0 indicate "strong binding affinity." This threshold is not standard in the literature; many studies use -5.0 or -6.0 as a cutoff for moderate binding, and -7.0 to -9.0 for strong binding. Please cite a relevant reference for your chosen threshold. More importantly, you did not perform any control docking (e.g., with known inhibitors or random compounds) to establish a baseline, nor did you validate the docking results with molecular dynamics simulations or experimental binding assays. Please add these controls or significantly temper your conclusions.

12. The conclusion states that SELP is "the most significant potential key regulatory gene" and that BCL2A1 and MMP1 are "potential therapeutic targets." However, you have provided no experimental validation whatsoever – no qPCR, no Western blot, no knockdown or overexpression studies, no animal models, and no clinical sample validation beyond the existing GEO datasets. Given that the GEO datasets were also used for discovery, this is circular reasoning. You must explicitly state that all findings are purely computational and require extensive experimental validation before any therapeutic claims can be made. Furthermore, the claim about traditional Chinese medicine compounds binding to BCL2A1 and MMP1 is highly speculative without any pharmacological testing. Please rewrite the conclusion to reflect the purely exploratory and hypothesis-generating nature of this work.

Reviewer #2: This manuscript applies an integrated bioinformatics and machine learning framework to identify shared genes and potential therapeutic targets between periodontitis and systemic juvenile idiopathic arthritis, however the study largely remains descriptive and does not provide a mechanistic or conceptual advancement beyond a standard analytical pipeline.

The overall design follows a highly conventional workflow (GEO， DEG, WGCNA, LASSO/SVM, SHAP, immune infiltration, molecular docking), and while technically sound, it does not introduce a new conceptual framework or biological insight; instead, focusing on identifying overlapping genes and building classification models, which is already a well-established strategy in cross-disease biomarker studies. As such, the work reads more as a routine data-driven screening effort rather than a hypothesis-driven or mechanism-oriented study. Importantly, the authors should explicitly acknowledge the limitations of such approaches, particularly the interpretability and reproducibility issues associated with bulk transcriptomic analyses; for this, citing "Genetic Expression in Cancer Research: Challenges and Complexity" and "Technical and Biological Biases in Bulk Transcriptomic Data Mining for Cancer Research" would help contextualize the findings and appropriately temper the conclusions.

A major concern is that the proposed mechanisms are not convincingly established. Although SELP, BCL2A1, and MMP1 are highlighted as key genes, the evidence is limited to differential expression, immune correlation, and ROC performance, without any demonstration of upstream regulation, pathway-level dissection, or causal relationships. Pathway enrichment (e.g., NF-κB, IL-17) is presented, but no direct experimental or computational evidence links these genes to specific signaling cascades in the studied context. In its current form, the study supports association rather than mechanism. To strengthen this aspect, the authors should consider functional validation strategies such as gene perturbation (knockdown/overexpression), pathway rescue assays, or at minimum a more rigorous discussion of signaling hierarchy. Relevant mechanistic literature could also be incorporated, for example, "WDR54 enhances NF-κB signaling to promote progression of hepatocellular carcinoma" or "Over-expression of prolyl hydroxylase-1 blocks NF-κB-mediated cyclin D1 expression and proliferation in lung carcinoma cells", which illustrate how NF-κB pathway involvement can be experimentally validated rather than inferred.

Another important limitation lies in the cross-tissue and cross-disease design. The PD datasets are derived from gingival tissue, whereas the sJIA datasets are derived from blood, yet the study directly performs DEG overlap and identifies "shared genes". This introduces a substantial confounding factor, as differences in tissue composition and immune cell distribution may dominate the signal, leading to apparent overlaps that reflect immune bias rather than true shared pathogenesis. This issue should be explicitly discussed and ideally addressed through additional normalization strategies or validation in matched tissue types.

The machine learning component, while extensive, appears somewhat disproportionate to its biological contribution. Multiple algorithms (LASSO, SVM-RFE, RF, XGB, SHAP) are applied, yet there is no independent external cohort validation or demonstration of clinical utility. SHAP analysis improves model interpretability but does not enhance biological insight. Therefore, the ML section currently functions more as a feature selection and visualization tool rather than a driver of new discoveries, and this distinction should be clarified.

The molecular docking analysis is also relatively weak and does not provide convincing support for therapeutic claims. Binding energy a lone is insufficient to infer biological relevance, and no molecular dynamics simulation, binding validation, or functional assays are presented. As such, this section appears largely decorative. The authors are encouraged to strengthen the methodological discussion by referencing prior integrative docking studies, such as "Clinicopathological correlation of PTPN3 expression in breast cancer and in silico drug screening against PTPN3 for therapeutics", and to clearly state the limitations of in silico predictions.

Finally, the immune-related findings could be further developed. While neutrophil enrichment and CD8+ T cell reduction are reported, the discussion remains relatively superficial and lacks integration with broader immunological frameworks. Expanding this section and incorporating references such as "Identification of new immune target and signaling for cancer immunotherapy" would improve the depth and translational relevance of the manuscript.

Reviewer #3: This is a well presented study that lays a good foundation for further in vitro investigation.

**********

**Have the authors made all data and (if applicable) computational code underlying the findings in their manuscript fully available?**

The PLOS Data policy requires authors to make all data and code underlying the findings described in their manuscript fully available without restriction, with rare exception (please refer to the Data Availability Statement in the manuscript PDF file). The data and code should be provided as part of the manuscript or its supporting information, or deposited to a public repository. For example, in addition to summary statistics, the data points behind means, medians and variance measures should be available. If there are restrictions on publicly sharing data or code —e.g. participant privacy or use of data from a third party—those must be specified.

Reviewer #1: None

Reviewer #2: **No:**  Data sets are publicly available via the listed/shared GEO accession numbers, but the analysis code has not been shared in a public repository as required by PLOS policy

Reviewer #3: Yes

**********

PLOS authors have the option to publish the peer review history of their article (what does this mean?). If published, this will include your full peer review and any attached files.

If you choose “no”, your identity will remain anonymous but your review may still be made public.

**Do you want your identity to be public for this peer review?**  For information about this choice, including consent withdrawal, please see our Privacy Policy.

Reviewer #1: **Yes:**  Mohammad Hossein Alizadeh Roknabadi

Reviewer #2: **Yes:**  Walter Odur

Reviewer #3: No

[NOTE: If reviewer comments were submitted as an attachment file, they will be attached to this email and accessible via the submission site. Please log into your account, locate the manuscript record, and check for the action link "View Attachments". If this link does not appear, there are no attachment files.]

**Figure resubmission:**

While revising your submission, we strongly recommend that you use PLOS’s NAAS tool (https://ngplosjournals.pagemajik.ai/artanalysis) to test your figure files. NAAS can convert your figure files to the TIFF file type and meet basic requirements (such as print size, resolution), or provide you with a report on issues that do not meet our requirements and that NAAS cannot fix.

After uploading your figures to PLOS’s NAAS tool - https://ngplosjournals.pagemajik.ai/artanalysis, NAAS will process the files provided and display the results in the "Uploaded Files" section of the page as the processing is complete. If the uploaded figures meet our requirements (or NAAS is able to fix the files to meet our requirements), the figure will be marked as "fixed" above. If NAAS is unable to fix the files, a red "failed" label will appear above. When NAAS has confirmed that the figure files meet our requirements, please download the file via the download option, and include these NAAS processed figure files when submitting your revised manuscript.

**Reproducibility:**

To enhance the reproducibility of your results, we recommend that authors of applicable studies deposit laboratory protocols in protocols.io, where a protocol can be assigned its own identifier (DOI) such that it can be cited independently in the future. Additionally, PLOS ONE offers an option to publish peer-reviewed clinical study protocols. Read more information on sharing protocols at https://plos.org/protocols?utm_medium=editorial-email&utm_source=authorletters&utm_campaign=protocols

Attachment Submitted filename: PCOMPBIOL-D-26-00673_reviewed.pdf

# Author comment: Author response to Decision Letter 1

**DOI:** 10.1371/journal.pcbi.1014669.r002

3 Jul 2026

Attachment Submitted filename: Point-by-point response to reviewers .docx

# Peer review: Decision Letter 1

**DOI:** 10.1371/journal.pcbi.1014669.r003

**Authors:** Can Yang, Muhammad Naseem

5 Aug 2026

Dear Dr Qi,

We are pleased to inform you that your manuscript 'Shared pathogenic genes and therapeutic targets in periodontitis and systemic juvenile idiopathic arthritis: an integrative bioinformatics and machine learning study' has been provisionally accepted for publication in PLOS Computational Biology.

Before your manuscript can be formally accepted you will need to complete some formatting changes, which you will receive in a follow up email. A member of our team will be in touch with a set of requests.

Please note that your manuscript will not be scheduled for publication until you have made the required changes, so a swift response is appreciated.

IMPORTANT: The editorial review process is now complete. PLOS will only permit corrections to spelling, formatting or significant scientific errors from this point onwards. Requests for major changes, or any which affect the scientific understanding of your work, will cause delays to the publication date of your manuscript.

Should you, your institution's press office or the journal office choose to press release your paper, you will automatically be opted out of early publication. We ask that you notify us now if you or your institution is planning to press release the article. All press must be co-ordinated with PLOS.

Thank you again for supporting Open Access publishing; we are looking forward to publishing your work in PLOS Computational Biology.

Best regards,

Muhammad Naseem, PhD

Academic Editor

PLOS Computational Biology

Can Yang

Section Editor

PLOS Computational Biology

***********************************************************

Dear Authors,

Thank you for submitting the revised version of your manuscript, which has now been reviewed again by our esteemed reviewers. Based on their comments and my own assessment, I am pleased to recommend your submitted manuscript for publication in PLOS Computational Biology.

Congratulations on this achievement, and thank you for choosing PLOS Computational Biology for your work.

Reviewer's Responses to Questions

**Comments to the Authors:**

**Please note here if the review is uploaded as an attachment.**

Reviewer #1: Accepted

Reviewer #2: The authors have engaged seriously with the concerns raised previously, and most points have been substantively addressed. However, I noticed some few concerns that must be addressed before the manuscript can be accepted, which are described below;

1. Gene naming inconsistency. The sixth gene in the new panel is called LGALS4 (HGNC:6565, chromosome 19q13.2, galectin-4) in the abstract and Section 2.4, LGALSL (HGNC:25012, chromosome 2p14, galectin-like protein) in the SHAP results in Section 2.5, and LGALS1 (NCBI Gene 3956, galectin-1) in the immune infiltration section. These are three independently catalogued genes. LGALSL deviates at 5 of 7 residues required for carbohydrate binding in galectins and may not bind carbohydrates at all, so it is not functionally interchangeable with the other two. If three different genes were used across different analysis steps, the panel is internally inconsistent. If one gene was named incorrectly, the correct symbol must be applied uniformly throughout the manuscript including the abstract.

2. Figure 8 caption. Panel E is captioned "Molecular Docking Diagram 1NA5 for 5WHI (ITGAM) and kaempferol." PDB 5WHI is the crystal structure of BCL2A1, not ITGAM, and appears to be carried over from the previous version where BCL2A1 was a docking target. The correct PDB code for ITGAM should be used and the 5WHI reference removed

**********

**Have the authors made all data and (if applicable) computational code underlying the findings in their manuscript fully available?**

The PLOS Data policy requires authors to make all data and code underlying the findings described in their manuscript fully available without restriction, with rare exception (please refer to the Data Availability Statement in the manuscript PDF file). The data and code should be provided as part of the manuscript or its supporting information, or deposited to a public repository. For example, in addition to summary statistics, the data points behind means, medians and variance measures should be available. If there are restrictions on publicly sharing data or code —e.g. participant privacy or use of data from a third party—those must be specified.

Reviewer #1: None

Reviewer #2: **No:**  GEO accession numbers are provided. Of the supporting files, only S3 (Batch Correction.R) is clearly analysis code. The core pipeline code (WGCNA, ML, SHAP, docking, MD) is not identifiable among the named files.

**********

PLOS authors have the option to publish the peer review history of their article (what does this mean?). If published, this will include your full peer review and any attached files.

If you choose “no”, your identity will remain anonymous but your review may still be made public.

**Do you want your identity to be public for this peer review?**  For information about this choice, including consent withdrawal, please see our Privacy Policy.

Reviewer #1: **Yes:**  Mohammad Hossein Alizadeh Roknabadi

Reviewer #2: **Yes:**  Walter Odur

# Editor report: Acceptance letter

**DOI:** 10.1371/journal.pcbi.1014669.r004

**Authors:** Can Yang, Muhammad Naseem

PCOMPBIOL-D-26-00673R1

Shared pathogenic genes and therapeutic targets in periodontitis and systemic juvenile idiopathic arthritis: an integrative bioinformatics and machine learning study

Dear Dr Qi,

I am pleased to inform you that your manuscript has been formally accepted for publication in PLOS Computational Biology. Your manuscript is now with our production department and you will be notified of the publication date in due course.

The corresponding author will soon be receiving a typeset proof for review, to ensure errors have not been introduced during production. Please review the PDF proof of your manuscript carefully, as this is the last chance to correct any errors. Please note that major changes, or those which affect the scientific understanding of the work, will likely cause delays to the publication date of your manuscript.

Soon after your final files are uploaded, unless you have opted out, the early version of your manuscript will be published online. The date of the early version will be your article's publication date. The final article will be published to the same URL, and all versions of the paper will be accessible to readers.

For Research, Software, and Methods articles, you will receive an invoice from PLOS for your publication fee after your manuscript has reached the completed accept phase. If you receive an email requesting payment before acceptance or for any other service, this may be a phishing scheme. Learn how to identify phishing emails and protect your accounts at https://explore.plos.org/phishing.

Thank you again for supporting PLOS Computational Biology and open-access publishing. We are looking forward to publishing your work!

With kind regards,

Sharmila Kamatchi

PLOS Computational Biology | Carlyle House, Carlyle Road, Cambridge CB4 3DN | United Kingdom ploscompbiol@plos.org | Phone +44 (0) 1223-442824 | ploscompbiol.org | @PLOSCompBiol
